# *In-situ* measurements of rare earth elements in deep sea sediments using nuclear methods

**DOI:** 10.1038/s41598-018-23148-1

**Published:** 2018-03-21

**Authors:** Jasmina Obhođaš, Davorin Sudac, Ilker Meric, Helge E. S. Pettersen, Milivoj Uroić, Karlo Nađ, Vlado Valković

**Affiliations:** 1 Ruđer Bošković Institute, Bijenička c. 54, Zagreb, Croatia; 2 Department of Electrical Engineering, Western Norway University of Applied Sciences, Inndalsveien 28, Bergen, Norway; 3 Consortium for Engineering Applications of Radioisotopes (CEAR), North Carolina State University, Raleigh, NC 27695 – 7909 USA; 4 Department of Oncology and Medical Physics, Haukeland University Hospital, Jonas Lies vei 65, 5021 Bergen, Norway; 5 Department of Physics and Technology, University of Bergen, Allégaten 55, Bergen, Norway

## Abstract

The prospecting activities for finding new rare earth elements (REE) sources have increased greatly in recent years. One of the main discoveries was announced in 2011 by Japanese researchers who found large quantities of REE on the ocean seafloor at the sea depths greater than 4,000 m. The classic approach to investigate REE in deep sea sediments is to obtain sediment samples by drilling that is followed by laborious laboratory analysis. This is very expensive, time consuming and not appropriate for exploring vast areas. In order to efficiently explore the ocean floor for REE deposits, the further development of affordable sensors is needed. Here, we propose two nuclear techniques for exploring REE in surface deep sea sediments: i) Passive measurement of lutetium-176 radioactivity, appropriate if long-term *in-situ* measurements are possible, and ii) The use of the neutron sensor attached to a remotely operated vehicle for rapid *in-situ* measurement of gadolinium by thermal neutron-capture. Since concentrations of lutetium and gadolinium show strong linear correlation to the total REE concentrations in deep sea sediments, it is possible to deduce the total REE content by measuring Lu or Gd concentrations only.

## Introduction

REE include 15 lanthanide elements, scandium (Sc) and yttrium (Y) which often occur together in same ore deposits and exhibit similar chemical characteristics. In the narrow sense and in our work, REE refer to lanthanides which are usually divided in Light REE (LREE): La, Ce, Pr, Nd, Pm and Sm, and Heavy REE (HREE): Eu, Gd, Tb, Dy, Ho, Er, Tm, Yb and Lu.

The development of modern technologies created ever growing demand for the REE, frequently causing shortage in supply and their skyrocketing pricing. The geostrategic importance of REE supplies came into focus in 2009 when China as a dominant producer started restricting REE export^1^. This clearly indicated the need for diversification of supply channels by development of new REE sources. The very promising new resources of REE are deep sea surface sediments as shown in the study by Kato *et al*.^2^. They have found concentrations up to 1,051 μg/g of total REE in surface deep-sea sediments of eastern South and central North Pacific. Authors presumed that ocean REE reserves are 1,000 times larger than the world current land reserves. At least as important, the ocean REE-rich sediments are enriched in HREE, the critical elements from the perspective of geostrategic importance and economic value^2^. In addition, REE-rich deep sea sediments do not contain Th and U that are usually associated with land REE deposits causing serious problems of radioactive waste and increasing the price of REE production^2^. The discovery of REE-rich deep sea sediments was followed by the Japanese announcement that they have found deep sea REE resources in their exclusive economic zone in the Pacific, and their intention and ability to exploit it^3^.

While there are many strategies for the land geochemical prospecting of REE^4^, currently the only way to explore vast areas of deep sea sediments for occurrence of REE is by taking samples obtained by drilling, piston corer or sediment grabber to the laboratory analysis. Conventional laboratory techniques for REE analysis include x-ray fluorescence, instrumental nuclear activation and inductively coupled plasma mass spectroscopy. We report here the great potential of two nuclear methods for *in-situ* prospecting of REE in surface deep sea sediments which may help researchers to more efficiently explore these potentially promising new REE resources and to understand better the global distribution of REE. The first method is based on passive measurement of naturally occurring radioactive isotope ^176^Lu, and the second is based on thermal neutron-capture of Gd by using a pulsed 14 MeV neutron generator.

## Methods

### Radiometric measurement of ^176^Lu

The isotope ^176^Lu has a natural abundance of 2.59% in natural Lu. Its half-life is 3.73 × 10^10^ years. ^176^Lu decays by β-decay producing stable isotope ^176^Hf with release of two intense characteristic gamma rays at 201.8 keV and 306.8 keV. Figure 1 of Supplementary information shows the experimental set-up for passive measurement of ^176^Lu radioactivity consisting of standard electrode coaxial Ge-detector with 76 mm radius and relative efficiency of 20%, placed within the shield made of iron and lead to reduce the background noise. The background counting frequency of the shielded detector was < 2 Hz. Sample of 99.9% grade lutetium (III) oxide obtained from the Metall Rare Earth Ltd, Shenzhen, China, was weighed within a plastic bag and put in the middle of a sample container containing 160 g of the sea sediment collected in the eastern Adriatic Sea. The minimum detection limit (MDL) for ^176^Lu was obtained experimentally as the smallest fraction of Lu within sea sediment that was possible to analyze with a precision of 3 σ.

### Gd thermal neutron-capture

Gd has the highest cross section for thermal neutron capture of naturally occurring chemical elements (49,000 barns). In order to investigate the expected response in prompt gamma-ray intensities of low Gd concentrations in deep sea sediments, we prepared the Monte Carlo (MC) simulation setup using the general purpose MC code, MCNP6.1^5^. The neutron source was modeled as a point source operated in pulsed mode, contained within a volume of air, and emitting 2 × 10^8^ 14 MeV neutrons towards the seabed in a narrow cone. The 3″ × 3″ NaI(Tl) detector was placed behind a 5 mm thick aluminum layer. The 30 cm long detector shielding made of iron was placed between detector and the neutron source. The pulse width was set to 10 μs and the pulse frequency to 10 kHz. In order to test the impact of seawater as a moderator of fast neutrons, the simulations were performed using both a 5 cm and a 10 cm thick layer of seawater between the setup and the seabed. Figure 2 of Supplementary information shows the MC geometry considered in this study. The energy spectra of prompt gamma-rays incident on the detector were scored for the time period when the neutron pulse was “OFF”. The simulated spectra were then used in the source definition of the next step (for both pure SiO_2_ and SiO_2_ containing Gd) where the pulse-height spectrum of the 3″ × 3″ NaI(Tl) detector was generated using the so-called pulse-height tally along with a Gaussian energy broadening function (GEB) to take into account the finite detector resolution. Example input files for the MC model are given as Supplementary material_1. The MC code MCNP6.1 can be obtained through the Radiation Safety Information Computational Center (RSICC), Oak Ridge, Tennessee, USA^6^. The MDL for Gd was estimated from the calibration line as $MDL=\frac{3.29-C\sqrt{{N}_{b}+3{\sigma }_{{N}_{b}}}}{{N}_{p}}$, where *C* is the known concentration of the element of interest, *N*_*b*_ is the counts from background and *N*_*p*_ is the number of counts under the characteristic prompt gamma-ray peak, or window, of the element of interest. The *N*_p_/*C* ratio, i.e. the sensitivity, can be found using the slope of the linear fit. *N*_b_ can be determined by the *y* intercept and *σ*_*Nb*_ is the standard deviation of the intercept^7^. The error was calculated as the error of the slope coefficient. For benchmarking purposes, Fig. 3 in the supplementary information provides a comparison between the double differential prompt gamma-ray production cross-sections in ^157^Gd obtained from the ENDF/B-VII.1 database^8^ and MCNP6.1 predicted prompt gamma-ray production in ^157^Gd due to thermal neutron capture.

## Results and Discussion

### Radiometric measurement of ^176^Lu

**Figure 1:**
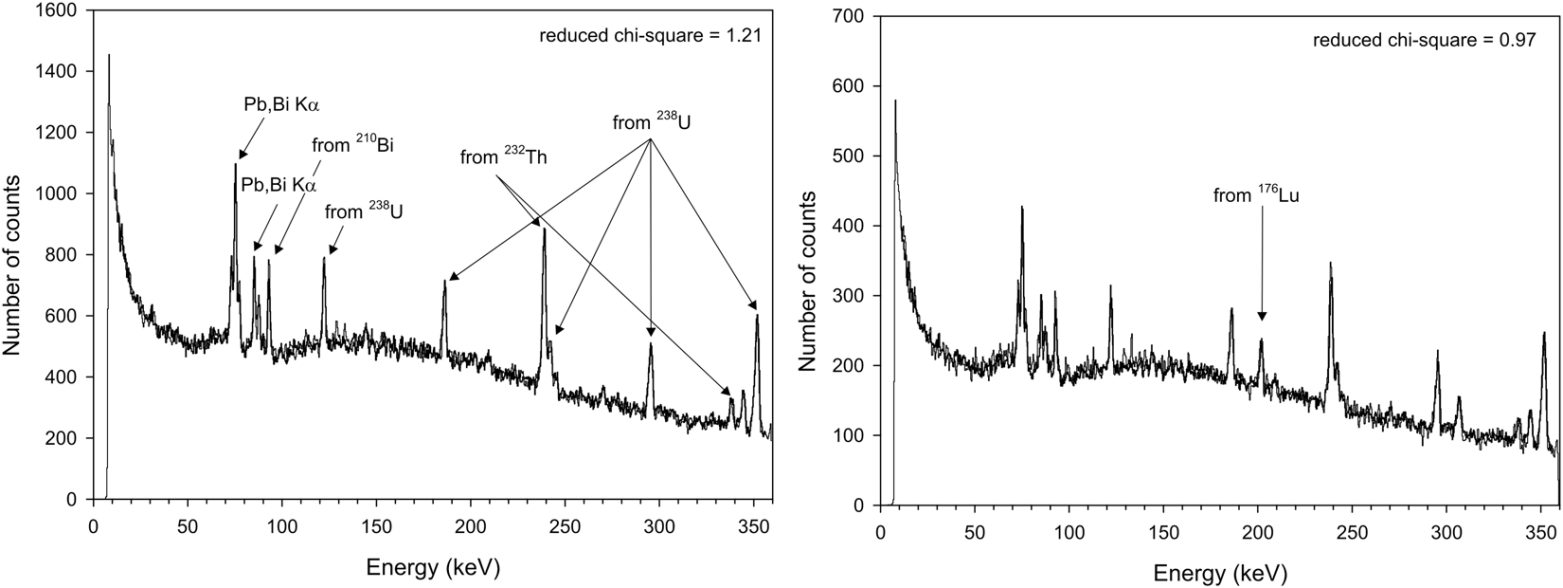
The left gamma ray spectrum is obtained from the pure sediment taken from the Adriatic Sea, weighing 160 g and the spectrum on the right is obtained from the same sample with addition of 6.62 mg (41.3 ppm) of Lu_2_O_3_.

Seven different concentrations of Lu were measured in order to obtain the calibration line. It was assumed that spectra were designated by four distinct contributors: background, Lu, Th and U. Each spectrum was fitted by eq. (1) where *a, b, c* and *d* are fitting parameters and *ch* stands for energy channel. For illustration, the fitting parameters obtained by the least squares method are presented in Table 1. Table 2 shows the dependence of the parameter *b*, measuring concentration value and precision, as a function of the integration time. These results imply that the integration time should be 65 h in order to obtain precision of 3 σ at very low concentrations. Figure 1 shows the spectra obtained from pure sediment and sediment containing Lu. The fitting parameter *b* determines the Lu content. The calibration line obtained for Lu in sea sediment is presented in Fig. 4 of Supplementary information.

**Table 1:** Fitting parameters.

Lu_2_O_3_ (mg/ppm)	a (background)	b (Lu)	c (U)	d (Th)	Measuring Time (h)
0	0.930 ± 0.003	0 ± 0.0005	0.060 ± 0.002	0.0069 ± 0.0002	60
0.41/2.56	0.926 ± 0.002	0.0016 ± 0.0004	0.06 ± 0.015	0.0071 ± 0.0001	131
1.18/7.37	0.921 ± 0.003	0.0029 ± 0.0005	0.066 ± 0.002	0.0070 ± 0.0002	62.5
3.42/21.37	0.909 ± 0.005	0.006 ± 0.001	0.073 ± 0.004	0.0065 ± 0.0004	20
5.44/34	0.914 ± 0.005	0.01 ± 0.001	0.062 ± 0.004	0.0067 ± 0.0004	17
6.62/41.37	0.897 ± 0.0045	0.013 ± 0.001	0.079 ± 0.004	0.0063 ± 0.0004	23
20/125	0.884 ± 0.004	0.043 ± 0.001	0.062 ± 0.0035	0.0059 ± 0.0003	24

**Table 2:** Fitting parameter b in dependence of different measuring time.

Measuring Time (h)	19	23.5	65	131
b (Lu)	0.002 ± 0.001	0.0015 ± 0.0009	0.0016 ± 0.0005	0.0016 ± 0.0004

The MDL for Lu obtained within this research was 2.56 ppm. Since Lu (III) oxide was not homogeneously distributed within the sediment, it can be expected that actual samples with homogeneous distribution of Lu will give slightly different results. In addition, it should be noted that the measurements were taken in the underground laboratory of the Ruđer Bošković Institute, which shows somewhat increased levels of natural radioactivity. A better shielding design would reduce the background radiation, the detection limit and the required measurement time. According to Kato *et al*.^2^ the Th and U contents in deep sea sediments are a small fraction of average crustal abundances, thus the deep sea environment would probably yield much better results.

1 $$\begin{array}{l}\begin{array}{c}{\chi }^{2}=\frac{sum{m}^{2}}{c{h}_{\max }-c{h}_{\min }-4+1}\\ \quad \quad \times \,\sum _{ch=c{h}_{\min }}^{c{h}_{\max }}\frac{{(a\times bac(ch)+b\times Lu(ch)+c\times U(ch)+d\times Th(ch)-\frac{Sample(ch)}{summ})}^{2}}{Sample(ch)}\end{array}\\ \begin{array}{c}\sum _{ch=c{h}_{\min }}^{c{h}_{\max }}bac(ch)=1,\,\sum _{ch=c{h}_{\min }}^{c{h}_{\max }}Lu(ch)=1,\,\sum _{ch=c{h}_{\min }}^{c{h}_{\max }}U(ch)=1,\,\sum _{ch=c{h}_{\min }}^{c{h}_{\max }}Th(ch)=1\\ \quad \quad \quad \quad \quad \quad \quad \times \,\sum _{ch=c{h}_{\min }}^{c{h}_{\max }}Sample(ch)=summ\end{array}\end{array}$$

### Gd thermal neutron-capture

**Figure 2:**
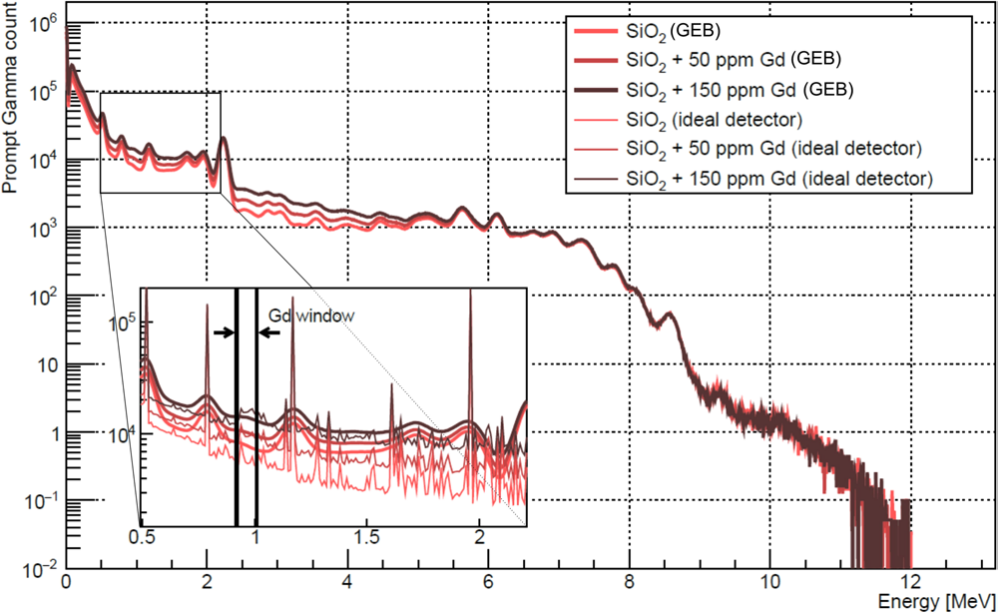
MC simulated prompt gamma-ray pulse-height spectra for the seabed considered as pure SiO_2_ or as a homogeneous mixture of SiO_2_ with 50 ppm and 150 ppm of Gd, excited by 14 MeV neutrons in pulsed mode (pulse width 10 μs and pulse frequency 10 kHz). Neutron sensor was positioned 5 cm above the seabed. Spectra were obtained for the pulse OFF mode. The spectra labeled as “GEB” refer to spectral data calculated using a Gaussian energy broadening function that takes into account the finite resolution of the 3″ × 3″ NaI(Tl) detector.

The MC simulations were run for pure SiO_2_ and for homogeneous mixtures of SiO_2_ and Gd with the neutron sensor positioned 5 cm or 10 cm above the seabed. Better results in terms of sensitivity and MDL were obtained for the position 5 cm above the seabed. Figure 2 shows the simulated pulse-height spectra of seabed containing different concentrations of Gd and 5 cm thick layer of seawater when the neutron pulse is OFF. The inset of Fig. 2 shows the window around 960 keV where Gd has at least 3 prominent gamma-ray lines. It can be seen that 50 ppm of Gd can be detected easily in the sea sediment. However, spectra prior to gaussian energy broadening indicate that a high-purity germanium or other detectors with better energy resolution than NaI(Tl) would be a better choice for Gd measurements. There is no difference between spectra of SiO_2_ and SiO_2_ with Gd when pulse is ON as shown in Fig. 5 of Supplementary information. Figure 6 of Supplementary information gives the calibration line obtained by MC simulations for the neutron sensor 5 cm distant from the seabed. The MDL for Gd calculated from this calibration line was 2.43 ± 0.17 ppm. The spectra and calibration line for the distance of neutron sensor 10 cm above the seabed are shown in Figs 7 and 8 of Supplementary Information, respectively. The MDL obtained for the sea layer of 10 cm was 4.87 ± 0.50 ppm.

**Figure 3:**
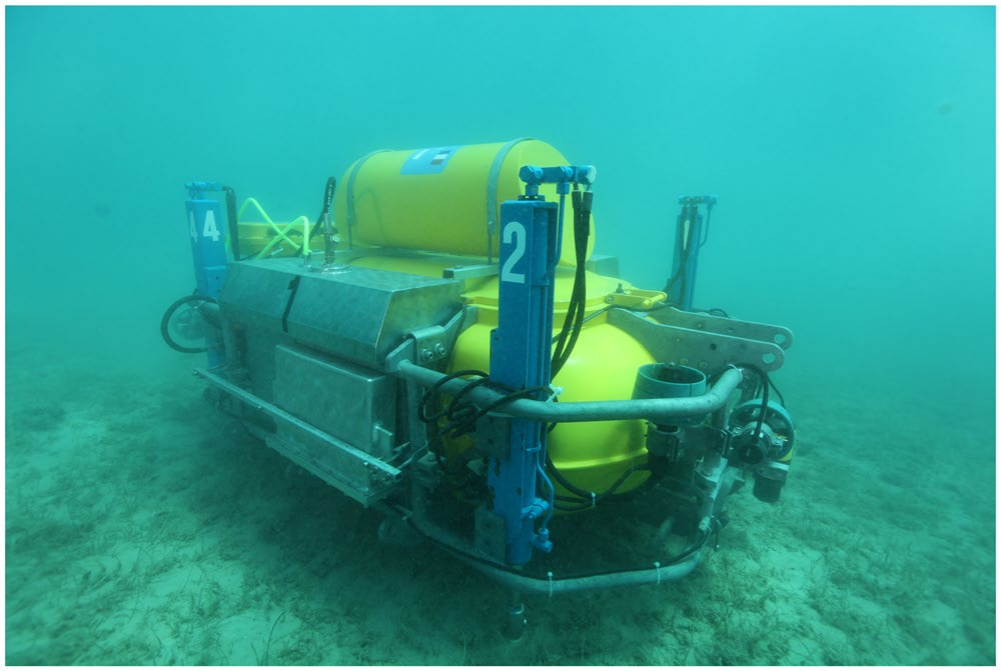
UNCOSS ROV carrying neutron sensor during the field test, Punat, island of Krk, Croatia, 06. May 2012^9^.

Based on these results a neutron sensor could be constructed and installed into the ROV similar to the one presented in Fig. 3. The ROV shown in Fig. 3 was developed under the EU FP7 UNCOSS project and it was designed for depths up to 50 m. The purpose of the ROV was to detect objects containing threat materials such as explosives and chemical warfare agents dumped at the seabed after the World War I and II^9^. This project proved that the neutron sensor can be used for underwater chemical analysis of objects and their contents, as well as for the chemical characterization of the seafloor in the background. The ROV and the neutron sensor can be adjusted for the purpose of the deep sea sediments prospecting. Concentrations of REE in undersurface sediments can be analyzed by usage of a corer attached to ROV. Such corer would be constructed to take sediment profiles 1–2 m long to be subsequently analysed *in-situ* by a sensor attached to ROV. Alternatively, a logging probe can be constructed and attached to ROV in order to analyse sediments directly up to several meters depth. Similar logging probes have been successfully applied in conjunction with underwater hydrocarbon explorations using both radioisotopes and electronic accelerator neutron sources^10–14^.

### Correlation of Gd and Lu with total REE in deep sea sediments

Rare-earth element contents data from Supplementary material of Kato *et al*.^2^ and Yasukawa *et al*.^15^ reveal that both Gd and Lu concentrations are strongly linearly correlated with the total REE content in deep sea surface sediments of the Pacific and the Indian Ocean, respectively. As shown in Kato *et al*.^2^, the deposition of REE in deep sea sediments of eastern South and central North Pacific Ocean is related to the mid-ocean ridge hydrothermal activity, reflecting the interplay among hydrothermal Fe-oxyhydroxide particulates and currents dispersing these particulates. The most enriched REE mud have occurred in pelagic deep-sea regions more than 2,000 km from mid-ocean ridges, in depths greater than 4,000 m, where high-REE materials have been deposited slowly (<0.5 cm kyr^−1^) without being significantly diluted by biogenous carbonate or silica, or by offshore terrigenous material^2^. Deng *et al*.^16^ identified redox potential and early diagenesis as important factors in formation of REE-rich sediments in addition to hydrothermal activities, absorption effects and low sedimentation rate. It has been shown by Yasukawa *et al*.^17^ that hydrothermal component does not play a role in formation of REE rich sediments in the Indian Ocean. The key factor for REE enrichment in the Indian Ocean identified by authors^17^ was the low sedimentation rate allowing both fish debris apatite and hydrogenous Fe-Mn oxyhydroxides to accumulate in the surface sediments.

**Figure 4:**
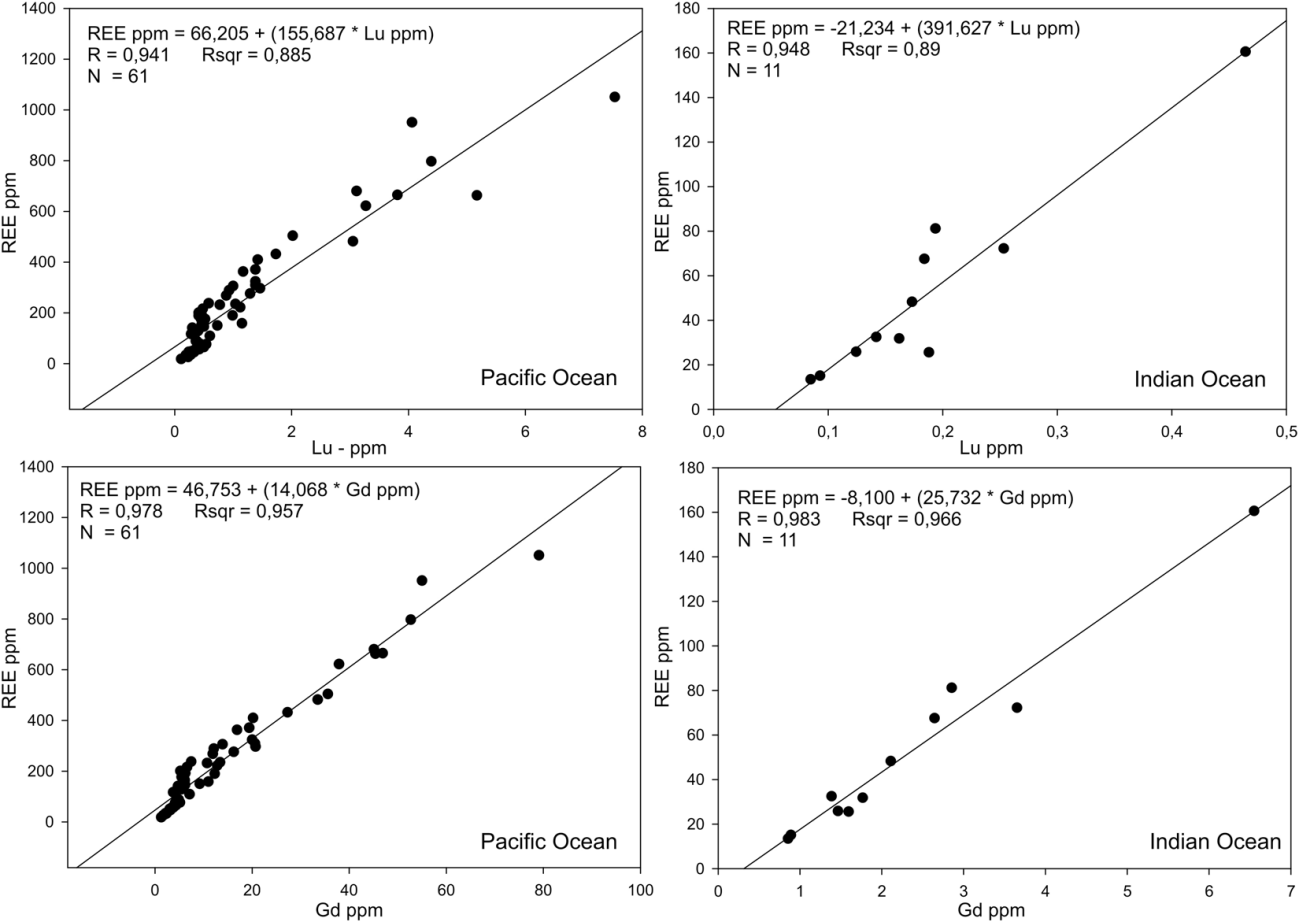
Correlation between Lu (up), Gd (down) and total REE content in deep sea surface sediments (<0,35 m depths) of the Pacific and the Indian Ocean. Data for Pacific Ocean were taken from supplementary material of Kato *et al*.^2^ and for the Indian Ocean from Yasukawa *et al*.^15^. Total REE content includes sum of La, Ce, Pr, Nd, Pm, Sm, Eu, Gd, Tb, Dy, Ho, Er, Tm, Yb and Lu concentrations.

Figure 4 presents correlations between concentrations of Lu and Gd, and the total REE content in surface deep sea sediments of the Pacific and the Indian Ocean (down to 35 cm depth). Lu concentration of 6.0 ± 0.3 ppm and Gd concentration of 68 ± 2 ppm indicates REE rich deposits, i.e. those exceeding 1,000 ppm of total REE, in deep sea sediments of the Pacific Ocean. According to data from Curtois and Clauer^18^, the Lu concentration of 7.6 ± 0.8 ppm indicates sediments of the Pacific Ocean enriched in REE. However, this study showing results from southeastern Pacific Ocean, reports only 8 measurements and fewer number of REE were analyzed. Figure 9 of the Supplementary information shows the correlation obtained for Lu and total REE based on data taken from Curtois and Clauer^18^. According to data taken from Yasukawa *et al*.^15^, Lu concentrations of 2.5 ± 0.3 ppm and Gd concentration of 38.9 ± 2.4 ppm indicates REE rich deposits in deep sea sediments of the Indian Ocean. It has to be noted that the number of samples considered, as well as the range of REE concentrations measured in the Indian Ocean was substantially smaller compared to those from the Pacific Ocean. However, the HREE/REE ratios in both data sets were similar, with averages of 0.2151 ± 0.0628 and 0.1972 ± 0.0526 for the Pacific and the Indian Ocean, respectively.

**Figure 5:**
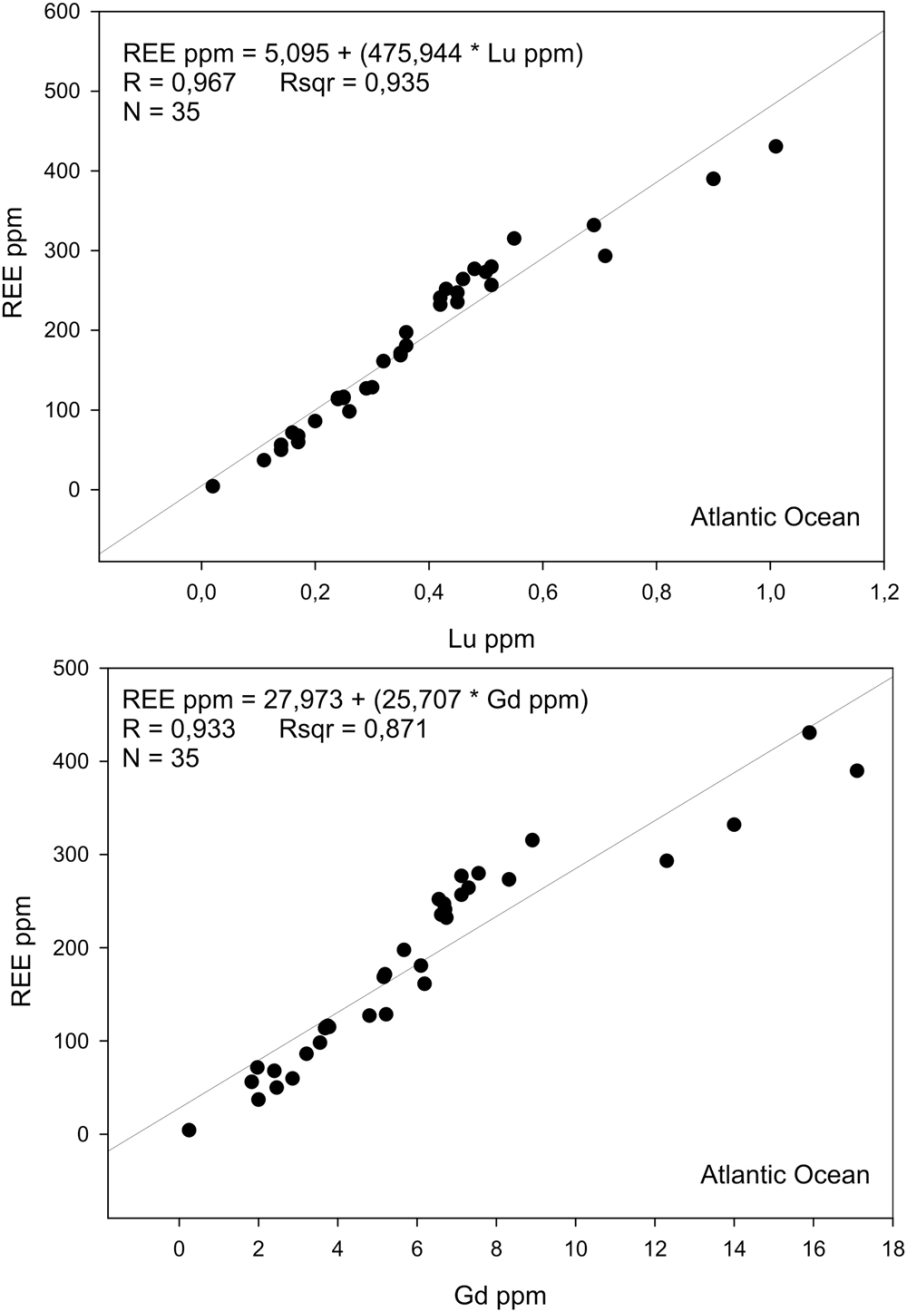
Correlation between Lu (up), Gd (down) and total REE content in deep sea sediments (<8.82 m depths) of the Atlantic Ocean. Data were taken from Menendez *et al*.^19^. Total REE content includes sum of La, Ce, Pr, Nd, Pm, Sm, Eu, Gd, Tb, Dy, Ho, Er, Tm, Yb and Lu concentrations.

The recent study published by Menendez *et al*.^19^ shows REY concentrations (lanthanides and Y) in 35 deep sea sediment samples taken in the North Atlantic Ocean from surface down to 8.82 m. According to their data^19^ Lu concentrations of 2.1 ± 0.1 and Gd concentrations of 38.9 ± 1.7 indicate REE rich sediments (Fig. 5). The HREE/REE ratio was 0,1375 ± 0.0395, which was lower compared to those in Pacific and Indian oceans.

## Conclusion

In this work we have shown that the total REE content in deep sea sediments can be to a first approximation deduced by measuring concentrations of Lu and Gd, by using passive analysis of radioactive ^176^Lu or by analysis of prompt gamma rays from thermal neutron capture of Gd. The described methods have been proposed for the first time for the *in-situ* prospecting of REE in the deep sea sediments. The MDL for Lu obtained with 3σ precision by laboratory measurements of ^176^Lu radioactivity was 2.56 ppm. The method is very simple and quite affordable, although a disadvantage might be the long measurement time. A larger number of detectors and expected lower background in deep sea sediments, due to low concentrations of uranium and thorium that cause interferences, can significantly lower the minimum detection limit and the measurement time. As it has been shown by the Monte Carlo modeling, low concentrations of Gd can be detected in the sea sediments by using the prompt gamma-ray neutron activation analysis technique. The MDL for Gd obtained by this study was 2.43 ± 0.17 ppm. The measurements can be carried out rapidly, however the cost of the neutron sensor and ROV construction is initially higher compared to construction of the set-up for the measurement of ^176^Lu. According to data on REE content in deep sea surface sediments from Kato *et al*.^2^, Lu concentration of 6.0 ± 0.3 ppm and Gd concentration of 68 ± 2 ppm indicates rich REE deposits (REE > 1,000 ppm) in the Pacific Ocean. For the Indian Ocean and according to data from Yasukawa *et al*.^15^, Lu concentration of 2.5 ± 0.3 ppm and Gd concentration of 38.9 ± 2.4 indicates REE rich deposits. Very similar values have been obtained for the Atlantic Ocean as it can been seen from data published by Menendez *et al*.^19^. According to their data Lu concentration of 2.1 ± 0.1 ppm and Gd concentration of 38.9 ± 1.7 ppm indicates rich REE deposits in the Atlantic Ocean.

## Electronic supplementary material

Supplementary Information 1

Supplementary Information 2

Supplementary Dataset

## References

[1] Hurst, C. China’s Rare Earth Elements Industry: What Can the West Learn? Institute for the Analysis of Global Security (IAGS), http://www.iags.org/rareearth0310hurst.pdf [Accessed on February 20, 2018] (2010).

[2] Kato YF (2011). Deep-sea mud in Pacific Ocean as potential resources of rare-earth elements. Nat.Geosci..

[3] Stratfor-Worldview. Deep-Sea mining as a Political Tool https://worldview.stratfor.com/analysis/deep-sea-mining-political-tool [Accessed on February 20, 2018] (2012).

[4] Castor, B. S. & Hedrick, J. B. Rare Earth Elements, in *Industrial Minerals and Rocks: Commodities, Markets, and Uses*, 2nd ed. J. E. Kogel, Ed. Society for Mining, Mettalurgy, and Exploration, Inc. (SME), Littleton Colorado, USA, 769–792 (2006).

[5] Goorley T (2012). Initial MCNP6 Release Overview. Nuclear Technology.

[6] Monte Carlo Methods, Codes, & Application Group. A General Monte Carlo N-Particle (MCNP) Transport Code. Los Alamos National Laboratory. https://mcnp.lanl.gov/ [Accessed on February 20, 2018].

[7] Chang HP (2017). Development of a method for on-line determination of chlorine impurity in crude oil by using fast neutrons. Fuel.

[8] Chadwick MB (2011). ENDF/B-VII.1: Nuclear Data for Science and Technology: Cross Sections, Covariances, Fission Product Yields and Decay Data. Nucl. Data Sheets.

[9] Valkovic V (2013). The Use of Alpha Particle Tagged Neutrons for the Inspection of Objects on the Sea Floor for the Presence of Explosives. NIM A.

[10] Peeples CR, Mickael M, Gardner RP (2010). On replacing Am–Be neutron sources in compensated porosity logging tools. Appl. Radiat. Isotopes.

[11] Chen AX, Antolak AJ, Leung K-N (2012). Electronic neutron sources for compensated porosity well logging. NIM A.

[12] Frankle CM, Dale GE (2013). Unconventional neutron sources for oil well logging. NIM A.

[13] Liu J (2016). A method to improve the sensitivity of neutron porosity measurement based on D-T source. J. Nat. Gas Sci. Eng..

[14] Al-Yaarubi, A. H. *et al*. Field Applications of the New Multidetector Slim Pulsed Neutron Logging Tool: Oman Case Studies. Abu Dhabi International Petroleum Exhibition & Conference, 7–10 November, Abu Dhabi, UAE, SPE-183548-MS, *Society of Petroleum Engineers*. 10.2118/183548-MS (2016).

[15] Yasukawa K (2016). Tracking the spatiotemporal variations of statistically independent components involving enrichment of rare-earth elements in deep-sea sediments. Sci. Rep..

[16] Deng Y (2017). Rare earth element geochemistry characteristics of seawater and porewater from deep sea in western Pacific. Sci. Rep..

[17] Yasukawa K (2015). Rare-earth, major, and trace element geochemistry of deep-sea sediments in the Indian Ocean: Implications for the potential distribution of REY-rich mud in the Indian Ocean. Geochem.J..

[18] Curtois C, Clauer N (1980). Rare earth elements and strontium isotopes in polimetallic noduls from southeastern Pacific Ocean. Sedimentology.

[19] Menendez A (2017). Controls on the distribution of rare earth elements in deep-sea sediments in the North Atlantic Ocean. Ore Geology Reviews..

