# Supplementary material for: In-situ measurements of rare earth elements in deep sea sediments using nuclear methods
Source: Sci Rep. 2018 Mar 21;8:4925. doi: 10.1038/s41598-018-23148-1 (PMC5862897; doi:10.1038/s41598-018-23148-1)
Supplement: Supplementary file 1 — Supplementary Information 1 [file 41598_2018_23148_MOESM1_ESM.pdf]

Supplementary Information 1: Additional figures

In-situ measurements of rare earth elements in deep sea sediments using nuclear methods

Jasmina Obhođaš, Davorin Sudac, Ilker Meric, Helge E. S. Pettersen,
Milivoj Uroić, Karlo Nađ, Vladivoj Valković

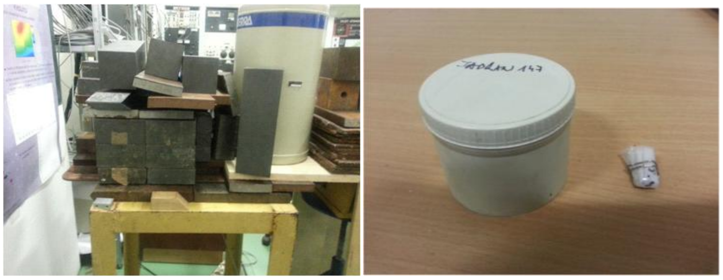

Fig. 1. Left: Experimental set-up for radiometric measurements of ^{176}Lu consisting of Ge detector and iron and lead shielding. Right: Plastic container of Φ=6 cm x 3.5 cm volume containing 160 g of the Adriatic sea sediment and folded plastic bag with natural lutetium (III) oxide.

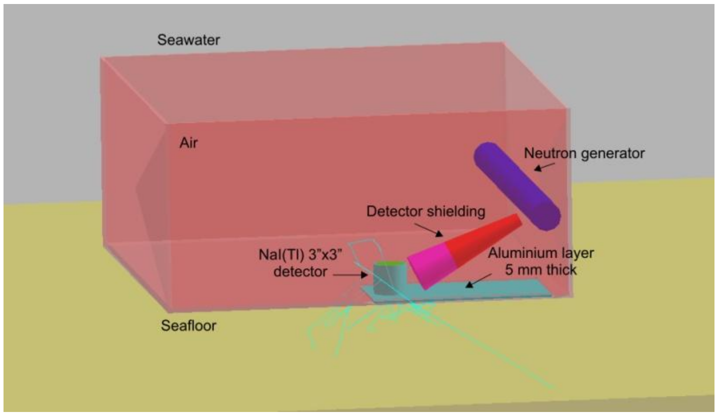

Fig.2. Three dimensional representation of the MC geometry along with few gamma-ray tracks used in simulations of Gd thermal neutron-capture in seabed sediments.

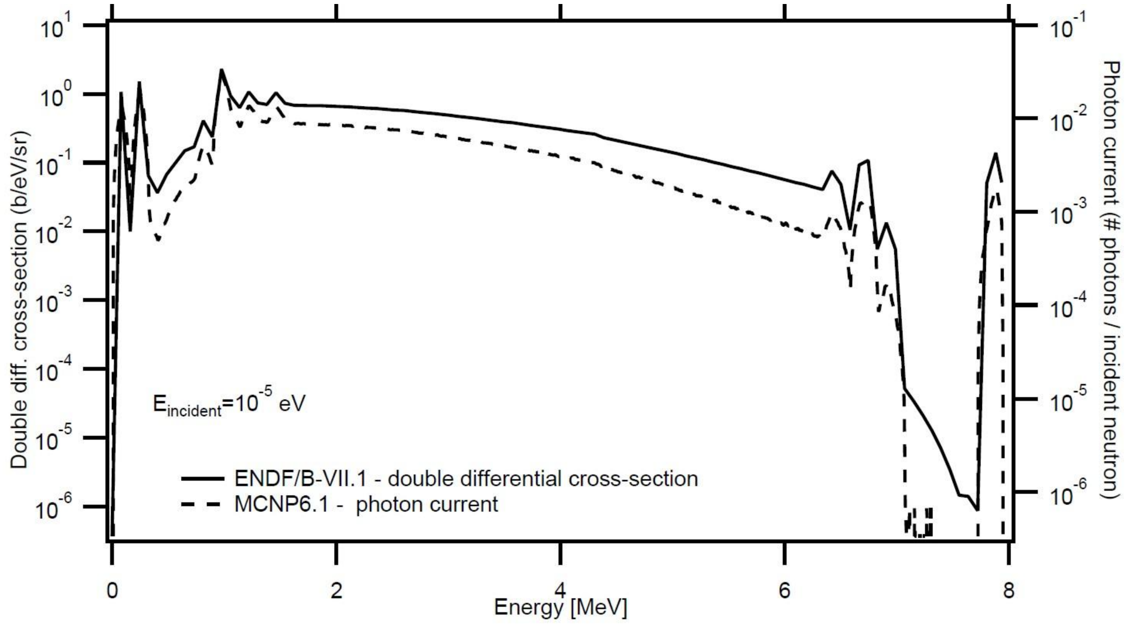

Fig.3. Comparison between the double differential prompt gamma-ray production cross-sections in ^{157}Gd obtained from the ENDF/B-VII.1 database and MCNP6.1 predicted prompt gamma-ray production in ^{157}Gd due to thermal neutron capture. MCNP6.1 data were generated through a simple simulation where a point-like neutron source with energy 10^{-5} eV was placed in the middle of a spherical volume of ^{157}Gd. All source particles were forced to collide immediately and all progeny escape without further interactions. The current of photons was scored on the surface of the spherical volume giving rise to a so-called surface current tally with the units of # photons/incident neutron. This was used in conjunction with energy binning to obtain the photon current as a function of energy. On the other hand, the ENDF database provides double differential cross-sections for (n,γ) reaction as a function of the neutron energy, including 10^{-5} eV. These two quantities are not directly comparable; however, it is important that the distribution of MCNP6.1 calculated current of photons follows closely that of the double different cross-section as a function of the prompt gamma-ray energy. The scale of the y-axes is logarithmic.

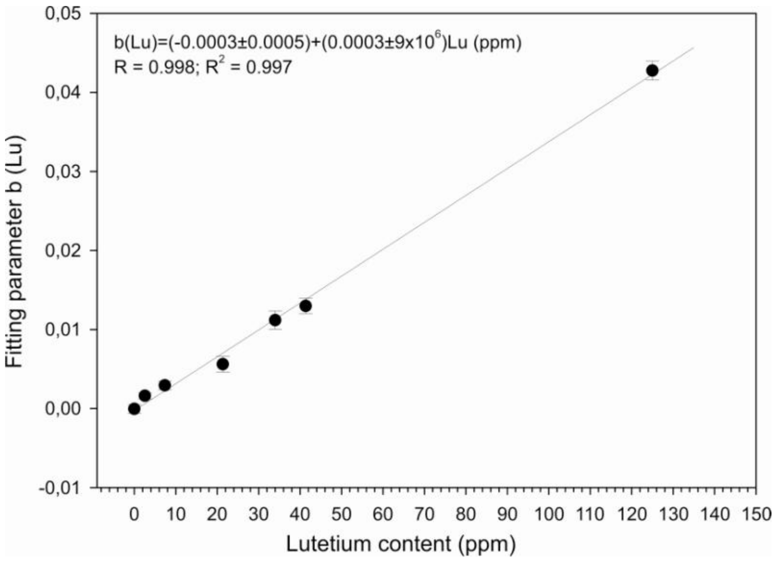

Fig. 4. Linear dependence of fitting parameter "b'' on Lu content. Calibration line for determination of ^{176}Lu in seabed sediments.

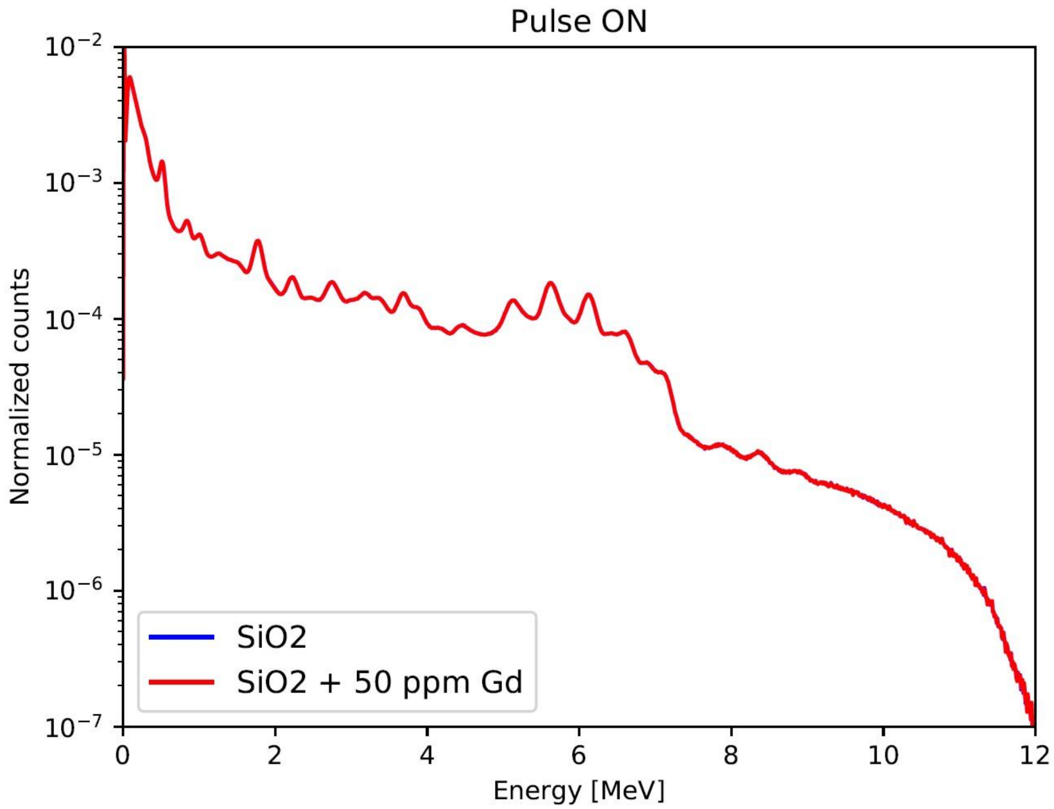

Fig 5. MC simulated prompt gamma-ray pulse-height spectra for the seabed considered as pure SiO_2 or as a homogeneous mixture of SiO_2 and 50 ppm of Gd, excited by 14 MeV neutrons in pulsed mode (pulse width 10 μs and pulse frequency 10 kHz). Neutron sensor was positioned 5 cm above the seabed. Spectra were obtained for the pulse ON mode. There is no difference between these two spectra.

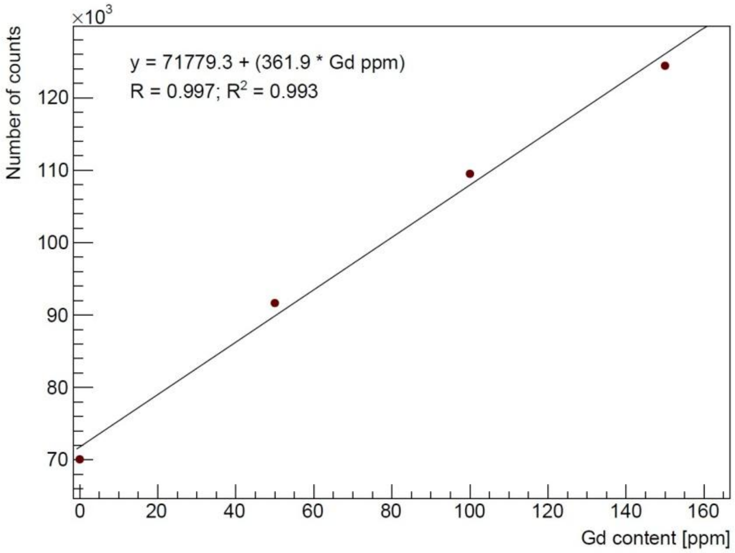

Fig. 6. Calibration line for Gd in seabed obtained by MC simulations for the sea layer 5 cm thick. The inherent statistical error at each data point follows from Poisson statistics and is less than 0.4% for all data points at 0 ppm, 50 ppm, 100 ppm and 150 ppm Gd concentrations.

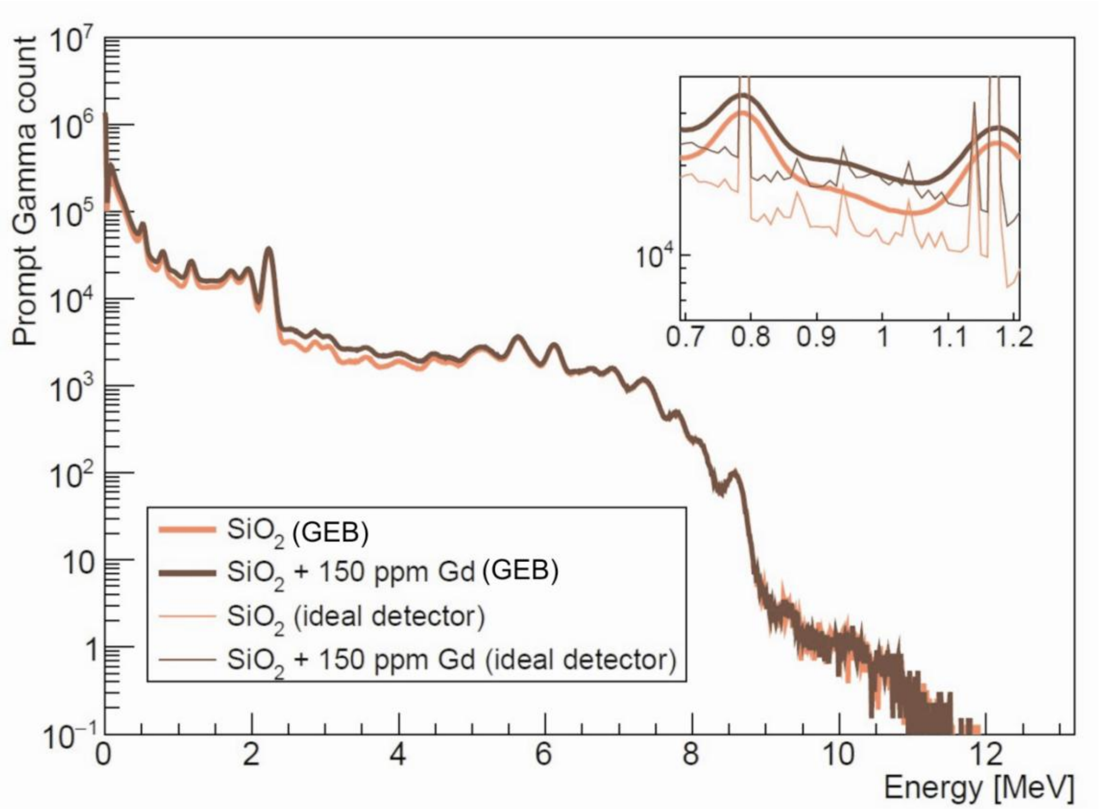

Fig. 7. MC simulated prompt gamma-ray pulse-height spectra for the seabed considered as pure SiO_2 or as a homogeneous mixture of SiO_2 and 150 ppm of Gd, excited by 14 MeV neutrons in pulsed mode (pulse width 10 μs and pulse frequency 10 kHz). Neutron sensor was positioned 10 cm above the seabed. Spectra were obtained for the pulse OFF mode. The spectra labeled as "GEB" refer to spectral data calculated using a Gaussian energy broadening function that takes into account the finite resolution of the 3"x3" NaI(Tl) detector. The inset shows the Gd window around 960 keV.

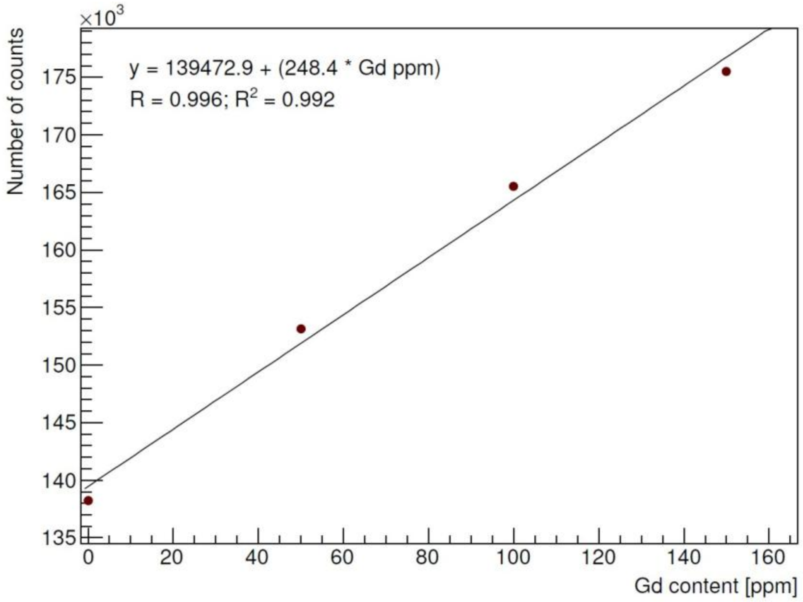

Fig.8. Calibration line for Gd in seabed obtained by MC simulations for the sea layer 10 cm thick. The inherent statistical error at each data point follows from Poisson statistics and is less than 0.4% for all data points at 0 ppm, 50 ppm, 100 ppm and 150 ppm Gd concentrations.

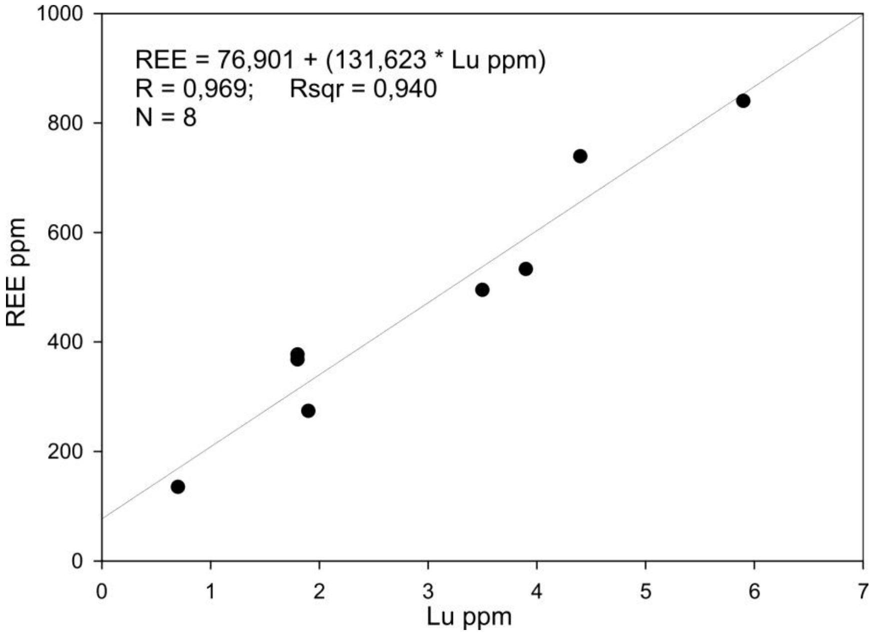

Fig.9. Correlation between Lu and total REE content in the deep sea surface sediments of southeastern Pacific Ocean. Data were taken from Curtois and Clauer[2]. Total REE content includes sum of La, Ce, Nd, Sm, Eu, Tb, Yb and Lu.

Supplementary references

1. M.B. Chadwick et.al., ENDF/B-VII.1: Nuclear Data for Science and Technology: Cross Sections, Covariances, Fission Product Yields and Decay Data, *Nucl. Data Sheets* **112,** 2887 (2011).

2. Curtois C. and Clauer N. Rare earth elements and strontium isotopes in polimetallic noduls from southeastern Pacific Ocean. *Sedimentology* **27**, 687-695 (1980).
