# Supplementary material for: In-situ measurements of rare earth elements in deep sea sediments using nuclear methods
Source: Sci Rep. 2018 Mar 21;8:4925. doi: 10.1038/s41598-018-23148-1 (PMC5862897; doi:10.1038/s41598-018-23148-1)
Supplement: Supplementary file 2 — Supplementary Information 2 [file 41598_2018_23148_MOESM2_ESM.pdf]

Supplementary Information 2: Monte Carlo files used for calculation of Gd in seabed

In-situ measurements of rare earth elements in deep sea sediments using nuclear methods

Jasmina Obhodas, Davorin Sudac, Ilker Meric, Helge E. S. Pettersen
Milivoj Uroic, Karlo Nad, Vladivoj Valkovic

Content

1. Pure SiO_2 / 5 cm seawater layer 2

2. SiO_2 + 150 ppm Gd / 5 cm seawater layer 7

3. Pure SiO_2 / GEB / 5 cm seawater layer 12

4. SiO_2 + 150 ppm Gd / GEB / 5 cm seawater layer 58

5. Pure SiO_2 / 10 cm seawater layer 104

6. SiO_2 + 150 ppm Gd / 10 cm seawater layer 109

7. Pure SiO_2 / GEB / 10 cm seawater layer 114

8. SiO_2 + 150 ppm Gd / GEB / 10 cm seawater layer 160

1. Pure SiO2 / 5 cm seawater layer

```
1   3  -7.9   -1  2                          IMP:N=1 IMP:P=1 $ neutron
generator
2   0         -2                             IMP:N=1 IMP:P=1 $ inner
generator
3   7  -3.67  -3                             IMP:N=1 IMP:P=0 $ NaI
4   8  -2.7    3 -4                          IMP:N=1 IMP:P=1 $ Al ring
6   8  -2.7   -6                             IMP:N=1 IMP:P=1 $ Al slab
7   5  -7.9  -20                             IMP:N=1 IMP:P=1 $ shadow
bar (Iron)
8  10 -11.35 -21                             IMP:N=1 IMP:P=1 $ shadow
bar (lead)
11 12  -1.43 -11:-12:-13:-14:-15:-16:-17:-18  IMP:N=1 IMP:P=1 $ Vessel
12 12  -1.43 -10                             imp:n=1 imp:p=1 $ Vessel
top
40  4  -1.225e-3  -101 #1 #2 #3 #4 #6 #7 #8 #11 #12 &
                                             IMP:N=1 IMP:P=1 $ Vessel
inside (air)
41  1  -1.025 -100 101                       IMP:N=1 IMP:P=1 $ Seawater
42  2  -2.196   -30                          imp:n=1 imp:p=1 $ Seabed
99  0       100 #42                        IMP:N=0 IMP:P=0  $ external
void

C ----------------------------------------------------------------------
-
C                    (((((      surfaces      )))))
C ----------------------------------------------------------------------
-
1    RCC   0  -5     0   0  100     0      3.81          $ neutron
generator
2    RCC   0  -4.6   0   0   99.2   0      3.41          $ neutron
generator
3    RCC -40   0   -19   0    0     7.62   3.81          $ 3"x3" NaI
cristal
4    RCC -40   0   -19   0    0     7.62   4.3           $ Al ring
C
6    RPP  -46     0  -13    13    -19.5  -19             $ Al slab
C
C    Vessel : polyester/kevlar=6/4 - half down part : 30kg
C
10   RPP -100   5  -16  96   29.5  30.5                  $ Top
11   RPP -100 -99  -16  96  -18.5  29.5                  $ left side
with Top
12   RPP    4   5  -16  96  -18.5  29.5                  $ right side
with Top
13   RPP -100 -46  -16  96  -19.5 -18.5                  $ left side of
the floor
14   RPP    0   5  -16  96  -19.5 -18.5                  $ right side of
the floor
15   RPP  -46   0  -16 -13  -19.5 -18.5                  $ front side of
the floor
16   RPP  -46   0   13  96  -19.5 -18.5                  $ back side of
the floor
```

```
17   RPP -100   5   95  96  -18.5  29.5                  $ front side
with Top
18   RPP -100   5  -16 -15  -18.5  29.5                  $ back side
with Top
C
20   1 TRC  -5     0  0  -19.99  0   0  1.75  3.92        $ Shadow Bar Fe
21   1 TRC -24.99  0  0  -10     0   0  3.92  5           $ Shadow Bar Pb
C
30   RPP -210  150  -150 200  -150  -24.5                $ seabed (SiO2
+ Gd)
100  RPP -210  150  -150 200  -24.5  150                    $ seawater
101  RPP -100.0001 5.0001 -16.0001 96.0001 -19.5001 30.5001 $ Vessel
inside

C ----------------------------------------------------------------------
-
C                 ((((      Transformation      )))))
C
C ----------------------------------------------------------------------
-
C    X' Y' Z' xx' yx' zx'  xy' yy' zy'  xz' yz' zz'
C
C  26 degrees rotation
C
*tr1 -1.8 0 1 26 90 64 90 0 90 116 90 26
C
C ----------------------------------------------------------------------
-
C
C ----------------------------------------------------------------------
-
C                ((((      Source definition      )))))
C ----------------------------------------------------------------------
-
C
MODE N P
c
C Neutron source definition, E=14 MeV, pulsed neutron source
C
sdef  erg=14  pos=0. 0.1 0.1  vec=-0.77715 0 -0.62932 par=1 dir=d1 tme=d2
si1  -1.0  0.993  1.0
sp1   0.0  0.0    1.0
si2 h 0 1000
sp2 d 0 1
lca 8j 1 1
C ----------------------------------------------------------------------
-
C                       ((((      Tally      )))))
C ----------------------------------------------------------------------
-
C
f11:p (3.1 3.2 3.3)  $ Surface current tally, scoring incident photon
energies
e11 0.01 1198i 12.0  $ Energy binning
```

```
t11: 1000 10000 15000000 $ Time binning
c
C ----------------------------------------------------------------------
-
C                     (((((      Materials     )))))
C ----------------------------------------------------------------------
-
C
c =========================== Sea water
=================================
C
M1        8016.80c -0.858142 &
          8017.80c -0.000347 &
          1001.80c -0.108186 &
          1002.80c -0.000025 &
         17035.80c -0.014503 &
         17037.80c -0.004899 &
         11023.80c -0.010801 &
         12024.80c -0.001007 &
         12025.80c -0.000133 &
         12026.80c -0.000152 &
         16032.80c -0.000861 &
         16033.80c -0.000007 &
         16034.80c -0.000041 &
         19039.80c -0.000372 &
         19041.80c -0.000028 &
         20040.80c -0.000387 &
         20042.80c -0.000003 &
         20043.80c -0.000001 &
         20044.80c -0.000009 &
         20048.80c -0.000001 &
         35079.80c -0.000034 &
         35081.80c -0.000033 &
          6000.80c -0.000028
C
c
c ============================ SiO2 ========================
=============
C
m2   14028.80c   -0.431005842210386 &
     14029.80c   -0.021971013648469 &
     14030.80c   -0.014491519640479 &
      8016.80c   -0.531253548601864 &
      8017.80c   -0.001278075898802
C
c
c ============================ Inox ========================
=============
C
M3       26054.80c -3.94109804245289E-02 &
         26056.80c -6.40977517311647E-01 &
         26057.80c -1.50755011378944E-02 &
         26058.80c -2.02600112593001E-03 &
         24050.80c -7.59652441069589E-03 &
```

```
         24052.80c -1.52167870331914E-01 &
         24053.80c -1.75848008017023E-02 &
         24054.80c -4.45080445568747E-03 &
         28058.80c -6.82087476171617E-02 &
         28060.80c -2.71743778762732E-02 &
         28061.80c -1.20121432630459E-03 &
         28062.80c -3.8875236702706E-03 &
         28064.80c -1.02813650998988E-03 &
         25055.80c -1.38E-02 &
         14028.80c -4.59367138104534E-03 &
         14029.80c -2.40907566547312E-04 &
         14030.80c -1.65421052407344E-04 &
         15031.80c -2.6E-04 &
          6000.80c -1.5E-04
c
C =========================== air WEIGHT CHEMICAL FORMULA
================
C
M4        7014.80c 0.8  &
          8016.80c 0.2
c
C =========================== Iron
=======================================
C
M5       26054.80c 0.058   &
         26056.80c 0.9172  &
         26057.80c 0.022   &
         26058.80c 0.0028
c
C =========================== NaI CHEMICAL FORMULA
=======================
C
M7       11023.80c  1 &
         53127.80c  1
c
C =========================== Aluminium
==================================
C
M8       13027.80c  1
c
C =========================== Lead
=======================================
C
M10     82204.80c -0.013781  82206.80c -0.239555  82207.80c -0.220743
        82208.80c -0.525921
C
C =========================== polyester/kevlar
===========================
C
C --> polyester/kevlar = 6/4
C
M12      1001.80c -0.056987   1002.80c -0.000013   6000.80c -0.635300
         7014.80c -0.051098   7015.80c -0.000202   8016.80c -0.256296
         8017.80c -0.000104
```

```
c
c ----------------------------------------------------------------------
-
nps 2.0e+8      $ Number of histories to be simulated
cut:n j 0       $ Neutron cut-off energy = 0.0 eV
cut:p j 0.001   $ Photon cut-off energy = 1.0 keV
```

2. SiO2 + 150 ppm Gd / 5 cm seawater layer

```
1   3  -7.9   -1  2                            IMP:N=1 IMP:P=1 $ neutron
generator
2   0         -2                               IMP:N=1 IMP:P=1 $ inner
generator
3   7  -3.67  -3                               IMP:N=1 IMP:P=0 $ NaI
4   8  -2.7    3 -4                            IMP:N=1 IMP:P=1 $ Al ring
6   8  -2.7   -6                               IMP:N=1 IMP:P=1 $ Al slab
7   5  -7.9  -20                               IMP:N=1 IMP:P=1 $ shadow
bar (Iron)
8  10 -11.35 -21                               IMP:N=1 IMP:P=1 $ shadow
bar (lead)
11 12  -1.43 -11:-12:-13:-14:-15:-16:-17:-18  IMP:N=1 IMP:P=1 $ Vessel
12 12  -1.43 -10                               imp:n=1 imp:p=1 $ Vessel
top
40  4  -1.225e-3  -101 #1 #2 #3 #4 #6 #7 #8 #11 #12 &
                                               IMP:N=1 IMP:P=1 $ Vessel
inside (air)
41  1  -1.025 -100 101                         IMP:N=1 IMP:P=1 $ Seawater
42  2  -2.19608 -30                            imp:n=1 imp:p=1 $ Seabed
99  0       100 #42                          IMP:N=0 IMP:P=0  $ external
void

C -------------------------------------------------------------------------
-
C                    (((((      surfaces      )))))
C -------------------------------------------------------------------------
-
1    RCC   0  -5     0   0  100     0      3.81           $ neutron
generator
2    RCC   0  -4.6   0   0   99.2   0      3.41           $ neutron
generator
3    RCC -40   0   -19   0    0     7.62   3.81           $ 3"x3" NaI
cristal
4    RCC -40   0   -19   0    0     7.62   4.3            $ Al ring
C
6    RPP  -46     0  -13    13    -19.5  -19              $ Al slab
C
C    Vessel : polyester/kevlar=6/4 - half down part : 30kg
C
10   RPP -100   5  -16  96   29.5  30.5                   $ Top
11   RPP -100 -99  -16  96  -18.5  29.5                   $ left side
with Top
12   RPP    4   5  -16  96  -18.5  29.5                   $ right side
with Top
13   RPP -100 -46  -16  96  -19.5 -18.5                   $ left side of
the floor
14   RPP    0   5  -16  96  -19.5 -18.5                   $ right side of
the floor
15   RPP  -46   0  -16 -13  -19.5 -18.5                   $ front side of
the floor
16   RPP  -46   0   13  96  -19.5 -18.5                   $ back side of
the floor
```

```
17   RPP -100   5   95  96  -18.5  29.5                  $ front side
with Top
18   RPP -100   5  -16 -15  -18.5  29.5                  $ back side
with Top
C
20   1 TRC  -5     0  0  -19.99  0   0  1.75  3.92        $ Shadow Bar Fe
21   1 TRC -24.99  0  0  -10     0   0  3.92  5           $ Shadow Bar Pb
C
30   RPP -210  150  -150 200  -150  -24.5                $ seabed (SiO2
+ Gd)
100  RPP -210  150  -150 200  -24.5  150                    $ seawater
101  RPP -100.0001 5.0001 -16.0001 96.0001 -19.5001 30.5001 $ Vessel
inside

C -----------------------------------------------------------------------
-
C                 (((((      Transformation      )))))
C
C -----------------------------------------------------------------------
-
C    X' Y' Z' xx' yx' zx'  xy' yy' zy'  xz' yz' zz'
C
C  26 degrees rotation
C
*tr1 -1.8 0 1 26 90 64 90 0 90 116 90 26
C
C -----------------------------------------------------------------------
-
C
C -----------------------------------------------------------------------
-
C                (((((      Source definition      )))))
C -----------------------------------------------------------------------
-
C
MODE N P
C
C Neutron source definition, E=14 MeV, pulsed neutron source
C
sdef  erg=14  pos=0. 0.1 0.1  vec=-0.77715 0 -0.62932 par=n dir=d1 tme=d2
si1  -1.0  0.993  1.0
sp1   0.0  0.0    1.0
si2 h 0 1000
sp2 d 0 1
lca 8j 1 1
C -----------------------------------------------------------------------
-
C                       (((((      Tally      )))))
C -----------------------------------------------------------------------
-
C
f11:p (3.1 3.2 3.3)  $ Surface current tally, scoring incident photon
energies
e11 0.01 1198i 12.0  $ Energy binning
```

```
t11: 1000 10000 15000000 $ Time binning
c
C ----------------------------------------------------------------------
-
C                    (((((     Materials     )))))
C ----------------------------------------------------------------------
-
C
c =========================== Sea water
=================================
C
M1         8016.80c -0.858142 &
           8017.80c -0.000347 &
           1001.80c -0.108186 &
           1002.80c -0.000025 &
          17035.80c -0.014503 &
          17037.80c -0.004899 &
          11023.80c -0.010801 &
          12024.80c -0.001007 &
          12025.80c -0.000133 &
          12026.80c -0.000152 &
          16032.80c -0.000861 &
          16033.80c -0.000007 &
          16034.80c -0.000041 &
          19039.80c -0.000372 &
          19041.80c -0.000028 &
          20040.80c -0.000387 &
          20042.80c -0.000003 &
          20043.80c -0.000001 &
          20044.80c -0.000009 &
          20048.80c -0.000001 &
          35079.80c -0.000034 &
          35081.80c -0.000033 &
           6000.80c -0.000028
C
c
c ============================ SiO2 + 150 ppm Gd========================
=============
C
m2   14028.80c   -0.430941191334055&
     14029.80c   -0.021967717996421&
     14030.80c   -0.014489345912533&
      8016.80c   -0.531173860569574&
      8017.80c   -0.001277884187417&
     64152.80c   -3.000000E-07&
     64154.80c   -3.270000E-06&
     64155.80c   -2.220000E-05&
     64156.80c   -3.070500E-05&
     64157.80c   -2.347500E-05&
     64158.80c   -3.726000E-05&
     64160.80c   -3.279000E-05
C
c
```

```
c =========================== Inox =======================
=============
C
M3       26054.80c -3.94109804245289E-02 &
         26056.80c -6.40977517311647E-01 &
         26057.80c -1.50755011378944E-02 &
         26058.80c -2.02600112593001E-03 &
         24050.80c -7.59652441069589E-03 &
         24052.80c -1.52167870331914E-01 &
         24053.80c -1.75848008017023E-02 &
         24054.80c -4.45080445568747E-03 &
         28058.80c -6.82087476171617E-02 &
         28060.80c -2.71743778762732E-02 &
         28061.80c -1.20121432630459E-03 &
         28062.80c -3.8875236702706E-03 &
         28064.80c -1.02813650998988E-03 &
         25055.80c -1.38E-02 &
         14028.80c -4.59367138104534E-03 &
         14029.80c -2.40907566547312E-04 &
         14030.80c -1.65421052407344E-04 &
         15031.80c -2.6E-04 &
          6000.80c -1.5E-04
c
C =========================== air WEIGHT CHEMICAL FORMULA
================
C
M4        7014.80c 0.8  &
          8016.80c 0.2
c
C =========================== Iron
=======================================
C
M5       26054.80c 0.058   &
         26056.80c 0.9172  &
         26057.80c 0.022   &
         26058.80c 0.0028
c
C =========================== NaI CHEMICAL FORMULA
======================
C
M7       11023.80c  1 &
         53127.80c  1
c
C =========================== Aluminium
==================================
C
M8       13027.80c  1
c
C =========================== Lead
=======================================
C
M10     82204.80c -0.013781  82206.80c -0.239555  82207.80c -0.220743
        82208.80c -0.525921
C
```

```
C ============================ polyester/kevlar
===========================
C
C --> polyester/kevlar = 6/4
C
M12      1001.80c -0.056987   1002.80c -0.000013   6000.80c -0.635300
         7014.80c -0.051098   7015.80c -0.000202   8016.80c -0.256296
         8017.80c -0.000104
C
c ---------------------------------------------------------------------
-
nps 2.0e+8     $ Number of histories to be simulated
cut:n j 0      $ Neutron cut-off energy = 0.0 eV
cut:p j 0.001  $ Photon cut-off energy = 1.0 keV
```

3.Pure SiO2 / GEB / 5 cm seawater layer

```
c     SiO2 - Pulse 'OFF'
c     Using re-normalized f1 data
c     Particle weight determined from the total of f1 data
c
c
c   Problem Cells
c
1  1 -3.67      1  -2  -3    imp:p=1     $ crystal NaI
2  2 -1.22e-3  -4  #1        imp:p=1     $ Inner world (air)
3  0            4            imp:p=0     $ Outer world (void)

c
c   Problem Surfaces
c
1  pz  0.0
2  pz  7.62
3  cz  3.81
4  so  100.0

c
c   Problem data cards
c
mode p                  $ photon only problem
c
c  NaI
c
m1   11023.80c   1
     53127.80c   1
c
c  Air
c
m2    7014.80c   0.8
      8016.80c   0.2
c
c   Source definition -- energy is now a prob. dist.
c
sdef erg=d2  rad=d1  pos=0.0 0.0 -3.0  vec=0 0 1  dir=1  ext=0
     axs=0 0 1 wgt=0.0347858
si1   0  3.81    $ radial sampling range: 0 to 3.81
sp1  -21 1       $ uniform sampling over the disk area
si2 h 0.000e+00  $ source energy binning / histogram
      1.000e-02
      2.000e-02
      3.000e-02
      4.000e-02
      5.000e-02
      6.000e-02
      7.000e-02
      8.000e-02
      9.000e-02
      1.000e-01
      1.100e-01
      1.200e-01
```

```
1.300e-01
1.400e-01
1.500e-01
1.600e-01
1.700e-01
1.800e-01
1.900e-01
2.000e-01
2.100e-01
2.200e-01
2.300e-01
2.400e-01
2.500e-01
2.600e-01
2.700e-01
2.800e-01
2.900e-01
3.000e-01
3.100e-01
3.200e-01
3.300e-01
3.400e-01
3.500e-01
3.600e-01
3.700e-01
3.800e-01
3.900e-01
4.000e-01
4.100e-01
4.200e-01
4.300e-01
4.400e-01
4.500e-01
4.600e-01
4.700e-01
4.800e-01
4.900e-01
5.000e-01
5.100e-01
5.200e-01
5.300e-01
5.400e-01
5.500e-01
5.600e-01
5.700e-01
5.800e-01
5.900e-01
6.000e-01
6.100e-01
6.200e-01
6.300e-01
6.400e-01
6.500e-01
6.600e-01
```

6.700e-01
6.800e-01
6.900e-01
7.000e-01
7.100e-01
7.200e-01
7.300e-01
7.400e-01
7.500e-01
7.600e-01
7.700e-01
7.800e-01
7.900e-01
8.000e-01
8.100e-01
8.200e-01
8.300e-01
8.400e-01
8.500e-01
8.600e-01
8.700e-01
8.800e-01
8.900e-01
9.000e-01
9.100e-01
9.200e-01
9.300e-01
9.400e-01
9.500e-01
9.600e-01
9.700e-01
9.800e-01
9.900e-01
1.000e+00
1.010e+00
1.020e+00
1.030e+00
1.040e+00
1.050e+00
1.060e+00
1.070e+00
1.080e+00
1.090e+00
1.100e+00
1.110e+00
1.120e+00
1.130e+00
1.140e+00
1.150e+00
1.160e+00
1.170e+00
1.180e+00
1.190e+00
1.200e+00

1.210e+00
1.220e+00
1.230e+00
1.240e+00
1.250e+00
1.260e+00
1.270e+00
1.280e+00
1.290e+00
1.300e+00
1.310e+00
1.320e+00
1.330e+00
1.340e+00
1.350e+00
1.360e+00
1.370e+00
1.380e+00
1.390e+00
1.400e+00
1.410e+00
1.420e+00
1.430e+00
1.440e+00
1.450e+00
1.460e+00
1.470e+00
1.480e+00
1.490e+00
1.500e+00
1.510e+00
1.520e+00
1.530e+00
1.540e+00
1.550e+00
1.560e+00
1.570e+00
1.580e+00
1.590e+00
1.600e+00
1.610e+00
1.620e+00
1.630e+00
1.640e+00
1.650e+00
1.660e+00
1.670e+00
1.680e+00
1.690e+00
1.700e+00
1.710e+00
1.720e+00
1.730e+00
1.740e+00

1.750e+00
1.760e+00
1.770e+00
1.780e+00
1.790e+00
1.800e+00
1.810e+00
1.820e+00
1.830e+00
1.840e+00
1.850e+00
1.860e+00
1.870e+00
1.880e+00
1.890e+00
1.900e+00
1.910e+00
1.920e+00
1.930e+00
1.940e+00
1.950e+00
1.960e+00
1.970e+00
1.980e+00
1.990e+00
2.000e+00
2.010e+00
2.020e+00
2.030e+00
2.040e+00
2.050e+00
2.060e+00
2.070e+00
2.080e+00
2.090e+00
2.100e+00
2.110e+00
2.120e+00
2.130e+00
2.140e+00
2.150e+00
2.160e+00
2.170e+00
2.180e+00
2.190e+00
2.200e+00
2.210e+00
2.220e+00
2.230e+00
2.240e+00
2.250e+00
2.260e+00
2.270e+00
2.280e+00

2.290e+00
2.300e+00
2.310e+00
2.320e+00
2.330e+00
2.340e+00
2.350e+00
2.360e+00
2.370e+00
2.380e+00
2.390e+00
2.400e+00
2.410e+00
2.420e+00
2.430e+00
2.440e+00
2.450e+00
2.460e+00
2.470e+00
2.480e+00
2.490e+00
2.500e+00
2.510e+00
2.520e+00
2.530e+00
2.540e+00
2.550e+00
2.560e+00
2.570e+00
2.580e+00
2.590e+00
2.600e+00
2.610e+00
2.620e+00
2.630e+00
2.640e+00
2.650e+00
2.660e+00
2.670e+00
2.680e+00
2.690e+00
2.700e+00
2.710e+00
2.720e+00
2.730e+00
2.740e+00
2.750e+00
2.760e+00
2.770e+00
2.780e+00
2.790e+00
2.800e+00
2.810e+00
2.820e+00

2.830e+00
2.840e+00
2.850e+00
2.860e+00
2.870e+00
2.880e+00
2.890e+00
2.900e+00
2.910e+00
2.920e+00
2.930e+00
2.940e+00
2.950e+00
2.960e+00
2.970e+00
2.980e+00
2.990e+00
3.000e+00
3.010e+00
3.020e+00
3.030e+00
3.040e+00
3.050e+00
3.060e+00
3.070e+00
3.080e+00
3.090e+00
3.100e+00
3.110e+00
3.120e+00
3.130e+00
3.140e+00
3.150e+00
3.160e+00
3.170e+00
3.180e+00
3.190e+00
3.200e+00
3.210e+00
3.220e+00
3.230e+00
3.240e+00
3.250e+00
3.260e+00
3.270e+00
3.280e+00
3.290e+00
3.300e+00
3.310e+00
3.320e+00
3.330e+00
3.340e+00
3.350e+00
3.360e+00

3.370e+00
3.380e+00
3.390e+00
3.400e+00
3.410e+00
3.420e+00
3.430e+00
3.440e+00
3.450e+00
3.460e+00
3.470e+00
3.480e+00
3.490e+00
3.500e+00
3.510e+00
3.520e+00
3.530e+00
3.540e+00
3.550e+00
3.560e+00
3.570e+00
3.580e+00
3.590e+00
3.600e+00
3.610e+00
3.620e+00
3.630e+00
3.640e+00
3.650e+00
3.660e+00
3.670e+00
3.680e+00
3.690e+00
3.700e+00
3.710e+00
3.720e+00
3.730e+00
3.740e+00
3.750e+00
3.760e+00
3.770e+00
3.780e+00
3.790e+00
3.800e+00
3.810e+00
3.820e+00
3.830e+00
3.840e+00
3.850e+00
3.860e+00
3.870e+00
3.880e+00
3.890e+00
3.900e+00

3.910e+00
3.920e+00
3.930e+00
3.940e+00
3.950e+00
3.960e+00
3.970e+00
3.980e+00
3.990e+00
4.000e+00
4.010e+00
4.020e+00
4.030e+00
4.040e+00
4.050e+00
4.060e+00
4.070e+00
4.080e+00
4.090e+00
4.100e+00
4.110e+00
4.120e+00
4.130e+00
4.140e+00
4.150e+00
4.160e+00
4.170e+00
4.180e+00
4.190e+00
4.200e+00
4.210e+00
4.220e+00
4.230e+00
4.240e+00
4.250e+00
4.260e+00
4.270e+00
4.280e+00
4.290e+00
4.300e+00
4.310e+00
4.320e+00
4.330e+00
4.340e+00
4.350e+00
4.360e+00
4.370e+00
4.380e+00
4.390e+00
4.400e+00
4.410e+00
4.420e+00
4.430e+00
4.440e+00

4.450e+00
4.460e+00
4.470e+00
4.480e+00
4.490e+00
4.500e+00
4.510e+00
4.520e+00
4.530e+00
4.540e+00
4.550e+00
4.560e+00
4.570e+00
4.580e+00
4.590e+00
4.600e+00
4.610e+00
4.620e+00
4.630e+00
4.640e+00
4.650e+00
4.660e+00
4.670e+00
4.680e+00
4.690e+00
4.700e+00
4.710e+00
4.720e+00
4.730e+00
4.740e+00
4.750e+00
4.760e+00
4.770e+00
4.780e+00
4.790e+00
4.800e+00
4.810e+00
4.820e+00
4.830e+00
4.840e+00
4.850e+00
4.860e+00
4.870e+00
4.880e+00
4.890e+00
4.900e+00
4.910e+00
4.920e+00
4.930e+00
4.940e+00
4.950e+00
4.960e+00
4.970e+00
4.980e+00

4.990e+00
5.000e+00
5.010e+00
5.020e+00
5.030e+00
5.040e+00
5.050e+00
5.060e+00
5.070e+00
5.080e+00
5.090e+00
5.100e+00
5.110e+00
5.120e+00
5.130e+00
5.140e+00
5.150e+00
5.160e+00
5.170e+00
5.180e+00
5.190e+00
5.200e+00
5.210e+00
5.220e+00
5.230e+00
5.240e+00
5.250e+00
5.260e+00
5.270e+00
5.280e+00
5.290e+00
5.300e+00
5.310e+00
5.320e+00
5.330e+00
5.340e+00
5.350e+00
5.360e+00
5.370e+00
5.380e+00
5.390e+00
5.400e+00
5.410e+00
5.420e+00
5.430e+00
5.440e+00
5.450e+00
5.460e+00
5.470e+00
5.480e+00
5.490e+00
5.500e+00
5.510e+00
5.520e+00

5.530e+00
5.540e+00
5.550e+00
5.560e+00
5.570e+00
5.580e+00
5.590e+00
5.600e+00
5.610e+00
5.620e+00
5.630e+00
5.640e+00
5.650e+00
5.660e+00
5.670e+00
5.680e+00
5.690e+00
5.700e+00
5.710e+00
5.720e+00
5.730e+00
5.740e+00
5.750e+00
5.760e+00
5.770e+00
5.780e+00
5.790e+00
5.800e+00
5.810e+00
5.820e+00
5.830e+00
5.840e+00
5.850e+00
5.860e+00
5.870e+00
5.880e+00
5.890e+00
5.900e+00
5.910e+00
5.920e+00
5.930e+00
5.940e+00
5.950e+00
5.960e+00
5.970e+00
5.980e+00
5.990e+00
6.000e+00
6.010e+00
6.020e+00
6.030e+00
6.040e+00
6.050e+00
6.060e+00

6.070e+00
6.080e+00
6.090e+00
6.100e+00
6.110e+00
6.120e+00
6.130e+00
6.140e+00
6.150e+00
6.160e+00
6.170e+00
6.180e+00
6.190e+00
6.200e+00
6.210e+00
6.220e+00
6.230e+00
6.240e+00
6.250e+00
6.260e+00
6.270e+00
6.280e+00
6.290e+00
6.300e+00
6.310e+00
6.320e+00
6.330e+00
6.340e+00
6.350e+00
6.360e+00
6.370e+00
6.380e+00
6.390e+00
6.400e+00
6.410e+00
6.420e+00
6.430e+00
6.440e+00
6.450e+00
6.460e+00
6.470e+00
6.480e+00
6.490e+00
6.500e+00
6.510e+00
6.520e+00
6.530e+00
6.540e+00
6.550e+00
6.560e+00
6.570e+00
6.580e+00
6.590e+00
6.600e+00

6.610e+00
6.620e+00
6.630e+00
6.640e+00
6.650e+00
6.660e+00
6.670e+00
6.680e+00
6.690e+00
6.700e+00
6.710e+00
6.720e+00
6.730e+00
6.740e+00
6.750e+00
6.760e+00
6.770e+00
6.780e+00
6.790e+00
6.800e+00
6.810e+00
6.820e+00
6.830e+00
6.840e+00
6.850e+00
6.860e+00
6.870e+00
6.880e+00
6.890e+00
6.900e+00
6.910e+00
6.920e+00
6.930e+00
6.940e+00
6.950e+00
6.960e+00
6.970e+00
6.980e+00
6.990e+00
7.000e+00
7.010e+00
7.020e+00
7.030e+00
7.040e+00
7.050e+00
7.060e+00
7.070e+00
7.080e+00
7.090e+00
7.100e+00
7.110e+00
7.120e+00
7.130e+00
7.140e+00

7.150e+00
7.160e+00
7.170e+00
7.180e+00
7.190e+00
7.200e+00
7.210e+00
7.220e+00
7.230e+00
7.240e+00
7.250e+00
7.260e+00
7.270e+00
7.280e+00
7.290e+00
7.300e+00
7.310e+00
7.320e+00
7.330e+00
7.340e+00
7.350e+00
7.360e+00
7.370e+00
7.380e+00
7.390e+00
7.400e+00
7.410e+00
7.420e+00
7.430e+00
7.440e+00
7.450e+00
7.460e+00
7.470e+00
7.480e+00
7.490e+00
7.500e+00
7.510e+00
7.520e+00
7.530e+00
7.540e+00
7.550e+00
7.560e+00
7.570e+00
7.580e+00
7.590e+00
7.600e+00
7.610e+00
7.620e+00
7.630e+00
7.640e+00
7.650e+00
7.660e+00
7.670e+00
7.680e+00

7.690e+00
7.700e+00
7.710e+00
7.720e+00
7.730e+00
7.740e+00
7.750e+00
7.760e+00
7.770e+00
7.780e+00
7.790e+00
7.800e+00
7.810e+00
7.820e+00
7.830e+00
7.840e+00
7.850e+00
7.860e+00
7.870e+00
7.880e+00
7.890e+00
7.900e+00
7.910e+00
7.920e+00
7.930e+00
7.940e+00
7.950e+00
7.960e+00
7.970e+00
7.980e+00
7.990e+00
8.000e+00
8.010e+00
8.020e+00
8.030e+00
8.040e+00
8.050e+00
8.060e+00
8.070e+00
8.080e+00
8.090e+00
8.100e+00
8.110e+00
8.120e+00
8.130e+00
8.140e+00
8.150e+00
8.160e+00
8.170e+00
8.180e+00
8.190e+00
8.200e+00
8.210e+00
8.220e+00

8.230e+00
8.240e+00
8.250e+00
8.260e+00
8.270e+00
8.280e+00
8.290e+00
8.300e+00
8.310e+00
8.320e+00
8.330e+00
8.340e+00
8.350e+00
8.360e+00
8.370e+00
8.380e+00
8.390e+00
8.400e+00
8.410e+00
8.420e+00
8.430e+00
8.440e+00
8.450e+00
8.460e+00
8.470e+00
8.480e+00
8.490e+00
8.500e+00
8.510e+00
8.520e+00
8.530e+00
8.540e+00
8.550e+00
8.560e+00
8.570e+00
8.580e+00
8.590e+00
8.600e+00
8.610e+00
8.620e+00
8.630e+00
8.640e+00
8.650e+00
8.660e+00
8.670e+00
8.680e+00
8.690e+00
8.700e+00
8.710e+00
8.720e+00
8.730e+00
8.740e+00
8.750e+00
8.760e+00

8.770e+00
8.780e+00
8.790e+00
8.800e+00
8.810e+00
8.820e+00
8.830e+00
8.840e+00
8.850e+00
8.860e+00
8.870e+00
8.880e+00
8.890e+00
8.900e+00
8.910e+00
8.920e+00
8.930e+00
8.940e+00
8.950e+00
8.960e+00
8.970e+00
8.980e+00
8.990e+00
9.000e+00
9.010e+00
9.020e+00
9.030e+00
9.040e+00
9.050e+00
9.060e+00
9.070e+00
9.080e+00
9.090e+00
9.100e+00
9.110e+00
9.120e+00
9.130e+00
9.140e+00
9.150e+00
9.160e+00
9.170e+00
9.180e+00
9.190e+00
9.200e+00
9.210e+00
9.220e+00
9.230e+00
9.240e+00
9.250e+00
9.260e+00
9.270e+00
9.280e+00
9.290e+00
9.300e+00

9.310e+00
9.320e+00
9.330e+00
9.340e+00
9.350e+00
9.360e+00
9.370e+00
9.380e+00
9.390e+00
9.400e+00
9.410e+00
9.420e+00
9.430e+00
9.440e+00
9.450e+00
9.460e+00
9.470e+00
9.480e+00
9.490e+00
9.500e+00
9.510e+00
9.520e+00
9.530e+00
9.540e+00
9.550e+00
9.560e+00
9.570e+00
9.580e+00
9.590e+00
9.600e+00
9.610e+00
9.620e+00
9.630e+00
9.640e+00
9.650e+00
9.660e+00
9.670e+00
9.680e+00
9.690e+00
9.700e+00
9.710e+00
9.720e+00
9.730e+00
9.740e+00
9.750e+00
9.760e+00
9.770e+00
9.780e+00
9.790e+00
9.800e+00
9.810e+00
9.820e+00
9.830e+00
9.840e+00

9.850e+00
9.860e+00
9.870e+00
9.880e+00
9.890e+00
9.900e+00
9.910e+00
9.920e+00
9.930e+00
9.940e+00
9.950e+00
9.960e+00
9.970e+00
9.980e+00
9.990e+00
1.000e+01
1.001e+01
1.002e+01
1.003e+01
1.004e+01
1.005e+01
1.006e+01
1.007e+01
1.008e+01
1.009e+01
1.010e+01
1.011e+01
1.012e+01
1.013e+01
1.014e+01
1.015e+01
1.016e+01
1.017e+01
1.018e+01
1.019e+01
1.020e+01
1.021e+01
1.022e+01
1.023e+01
1.024e+01
1.025e+01
1.026e+01
1.027e+01
1.028e+01
1.029e+01
1.030e+01
1.031e+01
1.032e+01
1.033e+01
1.034e+01
1.035e+01
1.036e+01
1.037e+01
1.038e+01

1.039e+01
1.040e+01
1.041e+01
1.042e+01
1.043e+01
1.044e+01
1.045e+01
1.046e+01
1.047e+01
1.048e+01
1.049e+01
1.050e+01
1.051e+01
1.052e+01
1.053e+01
1.054e+01
1.055e+01
1.056e+01
1.057e+01
1.058e+01
1.059e+01
1.060e+01
1.061e+01
1.062e+01
1.063e+01
1.064e+01
1.065e+01
1.066e+01
1.067e+01
1.068e+01
1.069e+01
1.070e+01
1.071e+01
1.072e+01
1.073e+01
1.074e+01
1.075e+01
1.076e+01
1.077e+01
1.078e+01
1.079e+01
1.080e+01
1.081e+01
1.082e+01
1.083e+01
1.084e+01
1.085e+01
1.086e+01
1.087e+01
1.088e+01
1.089e+01
1.090e+01
1.091e+01
1.092e+01

1.093e+01
1.094e+01
1.095e+01
1.096e+01
1.097e+01
1.098e+01
1.099e+01
1.100e+01
1.101e+01
1.102e+01
1.103e+01
1.104e+01
1.105e+01
1.106e+01
1.107e+01
1.108e+01
1.109e+01
1.110e+01
1.111e+01
1.112e+01
1.113e+01
1.114e+01
1.115e+01
1.116e+01
1.117e+01
1.118e+01
1.119e+01
1.120e+01
1.121e+01
1.122e+01
1.123e+01
1.124e+01
1.125e+01
1.126e+01
1.127e+01
1.128e+01
1.129e+01
1.130e+01
1.131e+01
1.132e+01
1.133e+01
1.134e+01
1.135e+01
1.136e+01
1.137e+01
1.138e+01
1.139e+01
1.140e+01
1.141e+01
1.142e+01
1.143e+01
1.144e+01
1.145e+01
1.146e+01

1.147e+01
1.148e+01
1.149e+01
1.150e+01
1.151e+01
1.152e+01
1.153e+01
1.154e+01
1.155e+01
1.156e+01
1.157e+01
1.158e+01
1.159e+01
1.160e+01
1.161e+01
1.162e+01
1.163e+01
1.164e+01
1.165e+01
1.166e+01
1.167e+01
1.168e+01
1.169e+01
1.170e+01
1.171e+01
1.172e+01
1.173e+01
1.174e+01
1.175e+01
1.176e+01
1.177e+01
1.178e+01
1.179e+01
1.180e+01
1.181e+01
1.182e+01
1.183e+01
1.184e+01
1.185e+01
1.186e+01
1.187e+01
1.188e+01
1.189e+01
1.190e+01
1.191e+01
1.192e+01
1.193e+01
1.194e+01
1.195e+01
1.196e+01
1.197e+01
1.198e+01
1.199e+01
1.200e+01

```
sp2  0.00000e+00     $ source energy bin probabilities
     1.30323E-06
     8.35863E-06
     2.73468E-05
     1.95711E-04
     4.57849E-04
     6.70357E-04
     7.46181E-04
     8.00568E-04
     7.22254E-04
     6.52735E-04
     5.99359E-04
     5.53753E-04
     5.18678E-04
     4.86924E-04
     4.46500E-04
     4.18932E-04
     3.95580E-04
     3.87566E-04
     3.58481E-04
     3.40913E-04
     3.20948E-04
     3.06333E-04
     2.97490E-04
     3.10103E-04
     2.88591E-04
     2.71111E-04
     2.50131E-04
     2.28133E-04
     2.15099E-04
     2.05190E-04
     1.87090E-04
     1.75054E-04
     1.65432E-04
     1.58877E-04
     1.51075E-04
     1.53846E-04
     1.38361E-04
     1.31368E-04
     1.25860E-04
     1.21157E-04
     1.17571E-04
     1.13414E-04
     1.09372E-04
     1.31770E-04
     1.02584E-04
     9.99494E-05
     1.02088E-04
     1.22857E-04
     9.18131E-05
     9.16273E-05
     9.59411E-05
     1.11238E-03
     6.15121E-05
```

6.34533E-05
5.78573E-05
5.81579E-05
5.66607E-05
5.46864E-05
5.38324E-05
5.27425E-05
5.14251E-05
5.18257E-05
4.98683E-05
5.62641E-05
4.84819E-05
4.78726E-05
4.77754E-05
4.65673E-05
4.51607E-05
4.78988E-05
4.60055E-05
4.25540E-05
4.25592E-05
4.15187E-05
4.07644E-05
4.50175E-05
4.01302E-05
3.91471E-05
6.97756E-04
3.24139E-05
3.21924E-05
3.45440E-05
3.07516E-05
3.72187E-05
3.33889E-05
3.27168E-05
3.95530E-05
3.60042E-05
3.05780E-05
3.13969E-05
2.93308E-05
3.02782E-05
2.76265E-05
4.27097E-05
3.25564E-05
2.80025E-05
2.71372E-05
2.76978E-05
3.12803E-05
2.61594E-05
2.59244E-05
3.18221E-05
2.80512E-05
3.71495E-05
2.77109E-05
2.48373E-05
2.70921E-05

2.50606E-05
2.77500E-05
2.42784E-05
2.42885E-05
2.38065E-05
2.43877E-05
7.35130E-05
2.32127E-05
2.37647E-05
8.18062E-04
3.24325E-05
1.92143E-05
1.84335E-05
2.14826E-05
1.87771E-05
1.87868E-05
2.14065E-05
1.83963E-05
1.97059E-05
2.51086E-05
4.37514E-05
1.97842E-05
1.73722E-05
1.90502E-05
1.70634E-05
5.17858E-05
1.71886E-05
1.95104E-05
1.80096E-05
1.66620E-05
2.74899E-05
1.77791E-05
1.61445E-05
2.02575E-05
1.64535E-05
1.73646E-05
1.62165E-05
1.66273E-05
1.57786E-05
1.59117E-05
1.55301E-05
1.57841E-05
2.03228E-05
1.66329E-05
2.39405E-05
2.61883E-05
1.61943E-05
1.61858E-05
1.58950E-05
1.67528E-05
1.47258E-05
1.52634E-05
1.70238E-05
1.17286E-04

2.09534E-05
3.35325E-05
1.69518E-05
4.18723E-05
2.16189E-05
1.44846E-05
2.11095E-05
2.02246E-05
1.72200E-05
2.09236E-05
1.53186E-05
3.16225E-05
2.12679E-05
2.28758E-05
1.44367E-05
1.45385E-05
2.28460E-05
3.14051E-05
1.54253E-05
1.88645E-05
1.44281E-05
2.49853E-05
1.46800E-05
1.97082E-05
2.25139E-05
1.61076E-05
1.32007E-05
1.41593E-05
1.47069E-05
1.39838E-05
1.50989E-05
1.62004E-05
2.64238E-05
1.43085E-05
9.80865E-04
1.19718E-05
3.32947E-05
1.41489E-05
1.90670E-05
1.82553E-05
1.60128E-05
3.21582E-05
3.73720E-05
2.69731E-05
1.17785E-05
1.28235E-05
3.59090E-05
1.21172E-05
5.92641E-05
1.66877E-05
1.80047E-05
1.24337E-05
1.83824E-05
1.15104E-05

3.27312E-05
1.15813E-05
1.93480E-05
1.33130E-05
1.19511E-05
2.69670E-05
1.39163E-05
6.15095E-03
3.18186E-05
9.33966E-06
1.12031E-05
5.28169E-06
5.37159E-06
2.54607E-05
3.33965E-06
3.17884E-06
3.58576E-05
1.06048E-05
3.04032E-06
4.24453E-06
6.71950E-06
5.54942E-06
3.17160E-06
7.55228E-06
8.93761E-06
9.16050E-06
2.37198E-05
1.48879E-05
2.92467E-06
3.69063E-06
3.99280E-06
3.50351E-05
2.77991E-06
1.73203E-05
1.62182E-05
4.94311E-06
9.08921E-06
1.42648E-05
1.72635E-05
1.14175E-05
6.48633E-06
1.00291E-05
6.14564E-06
7.62690E-06
1.61596E-05
2.77820E-06
1.11328E-05
2.50656E-05
8.08467E-06
1.15947E-05
5.44854E-06
6.32598E-06
5.30868E-05
7.72879E-06

4.72981E-06
2.74525E-06
7.39860E-06
8.62921E-06
2.63980E-06
1.05073E-05
1.13358E-05
2.58101E-06
2.52175E-06
5.93172E-06
1.15577E-05
2.30501E-05
1.84264E-05
9.82928E-06
4.22466E-06
4.25664E-05
2.40061E-06
2.10820E-04
3.08083E-05
2.12806E-06
1.99737E-05
3.00909E-06
2.17569E-06
2.65627E-06
2.01703E-06
6.55538E-06
7.28586E-06
2.06360E-05
3.60163E-05
3.90672E-06
3.21950E-05
2.55453E-05
3.89269E-05
5.33034E-06
1.75374E-05
3.47517E-06
5.47234E-06
1.22733E-04
2.06715E-06
5.05395E-06
4.93714E-06
9.84897E-06
3.41311E-05
2.48926E-06
5.37742E-06
1.42769E-06
6.04476E-06
1.83874E-06
1.76782E-06
2.40593E-06
4.72007E-06
4.78757E-06
4.38995E-06
1.99835E-06

2.06438E-06
4.66194E-06
1.18223E-05
4.43812E-06
6.08461E-06
1.64371E-06
7.65583E-06
4.45591E-06
1.17011E-05
1.62824E-06
2.94928E-05
1.02435E-05
2.02586E-06
3.79680E-06
2.13724E-05
2.94196E-06
2.89158E-06
1.66713E-06
3.83885E-06
3.07420E-05
7.73414E-06
1.51883E-06
4.45428E-06
1.60220E-05
5.04242E-06
1.85580E-06
2.01048E-06
2.02396E-05
3.83708E-06
4.00199E-06
1.78359E-04
1.66276E-06
7.06917E-06
3.78835E-05
1.24309E-06
3.07782E-05
2.98495E-05
1.47169E-05
5.05536E-06
9.96771E-06
1.13164E-05
3.58616E-06
1.20581E-06
1.94353E-05
1.77783E-06
8.98153E-06
1.31660E-06
8.71788E-06
1.32154E-06
2.84983E-06
7.57484E-06
1.42732E-05
1.09381E-06
1.48794E-06

9.93618E-06
1.17537E-06
2.95678E-06
2.57887E-06
1.02855E-06
6.83269E-05
1.04845E-06
8.46012E-06
4.93550E-06
7.43079E-06
9.09441E-06
1.10308E-06
1.51380E-06
1.70874E-06
3.38666E-06
2.79397E-06
1.52943E-06
1.02169E-06
8.18523E-06
1.54899E-05
5.70863E-06
4.21397E-05
3.57981E-06
4.49452E-06
2.65406E-06
7.28525E-06
9.69199E-07
3.77640E-06
2.21701E-05
9.16007E-06
1.23948E-06
3.01445E-05
3.11888E-06
1.02044E-06
4.54394E-06
2.63889E-06
2.71265E-05
1.41936E-06
8.99359E-07
6.70100E-06
1.70389E-06
1.63713E-06
1.99944E-06
5.00618E-06
5.54594E-06
9.66989E-07
1.12029E-06
9.58499E-07
8.47005E-07
1.59279E-05
1.33083E-06
7.85503E-07
1.57010E-05
2.35546E-06

8.47235E-07
1.23909E-06
9.57309E-07
1.03640E-06
6.04454E-06
1.33353E-06
2.40265E-06
1.07885E-06
1.01204E-06
3.62594E-06
1.27699E-05
4.85757E-06
2.23833E-06
3.72977E-05
4.85792E-06
1.60510E-06
1.74063E-06
1.21499E-06
3.31241E-06
1.32866E-06
5.79300E-06
1.74044E-05
8.77245E-07
1.69740E-05
6.13710E-06
9.76998E-07
1.62555E-06
1.31762E-05
5.19621E-06
9.42417E-07
2.48338E-05
1.91365E-06
1.37493E-06
9.57291E-07
2.06184E-06
6.34245E-06
1.41125E-06
3.11234E-06
1.13161E-05
1.03799E-06
8.91952E-07
2.58230E-05
1.50727E-05
2.26936E-06
1.07454E-05
2.83881E-06
7.96056E-07
8.73322E-07
1.92107E-06
3.08452E-06
8.59538E-06
7.63051E-06
1.46552E-06
1.06151E-06

9.77680E-07
8.64840E-07
8.07567E-07
4.51409E-06
1.10956E-06
8.02312E-06
1.05104E-06
8.42708E-07
1.84372E-04
6.30606E-05
7.12089E-06
6.29199E-07
1.28985E-04
1.16051E-05
5.02268E-07
2.02144E-06
1.71315E-05
5.98059E-07
6.90694E-07
1.30241E-06
6.47181E-07
9.65459E-07
5.90070E-06
1.88944E-06
7.91038E-07
1.53693E-05
1.26012E-06
1.89156E-06
7.45652E-06
4.20892E-06
7.39890E-06
6.38256E-07
5.42031E-07
5.92862E-07
5.46060E-07
7.95952E-06
6.65864E-07
5.90195E-07
7.64148E-07
2.05367E-05
6.62625E-07
4.94115E-06
2.36475E-06
7.44266E-07
1.11122E-06
1.33602E-06
8.84892E-07
8.00553E-07
5.82683E-07
6.54605E-07
1.52499E-06
6.70965E-07
2.29067E-06
6.31185E-07

8.00946E-07
7.15243E-07
6.04476E-06
7.22251E-07
7.37359E-07
6.99209E-07
1.34924E-06
6.80791E-07
3.71146E-06
6.38582E-07
1.08185E-06
7.75702E-07
6.02967E-05
5.90392E-07
1.13024E-06
5.66070E-07
5.79756E-07
8.97095E-07
5.55188E-07
2.26340E-05
6.20257E-07
1.34056E-05
3.45674E-06
5.64174E-07
2.82256E-06
6.44018E-07
5.06757E-07
6.21919E-07
5.89314E-07
5.02615E-07
6.14389E-07
1.56978E-05
1.91147E-04
5.42365E-07
1.98212E-05
7.89058E-07
3.19204E-06
1.45135E-06
6.40012E-06
7.75377E-07
7.56067E-07
5.00806E-07
5.72220E-07
5.59027E-07
4.98417E-07
4.95188E-07
4.50755E-07
1.35094E-06
4.48907E-07
4.42459E-07
5.30503E-07
3.99494E-05
4.74871E-07
1.21885E-05

4.48798E-07
4.30038E-07
7.35173E-06
4.28222E-07
5.23979E-07
5.35047E-07
8.29801E-07
6.48145E-07
1.27906E-05
6.87744E-07
4.29697E-07
1.51053E-06
2.15068E-06
5.42736E-07
6.04467E-07
3.13817E-05
8.10403E-07
7.27106E-06
7.38816E-04
1.62947E-05
6.50556E-07
5.37944E-07
3.13474E-07
3.39797E-07
3.22990E-07
4.78814E-06
2.87261E-07
1.71790E-06
3.23754E-07
2.57271E-07
2.90709E-07
2.73497E-07
2.81781E-06
1.66092E-05
4.62808E-07
4.93168E-07
4.89020E-07
2.52623E-07
3.94575E-06
6.93942E-07
2.86537E-06
2.34982E-06
3.03954E-07
3.20025E-07
4.08974E-05
1.57811E-06
9.40428E-06
2.77205E-07
2.29655E-07
1.05071E-05
2.10841E-07
1.71799E-06
2.98800E-07
2.66921E-07

2.32807E-07
5.49670E-06
2.69572E-07
5.14306E-07
2.58888E-07
1.81611E-07
2.56846E-07
6.17901E-06
2.48514E-07
2.74952E-07
2.45133E-07
4.04768E-07
2.31560E-07
2.04684E-07
2.84840E-04
1.68011E-04
2.58743E-07
7.63235E-06
1.82278E-07
2.11884E-07
1.89795E-07
1.78287E-07
1.79777E-07
1.88233E-07
2.98274E-06
1.55895E-07
1.35149E-07
3.76183E-06
1.69724E-07
1.19041E-07
1.64536E-07
2.30873E-07
2.43741E-07
1.69611E-07
3.73559E-07
4.82420E-07
9.85032E-07
1.15960E-07
1.93456E-07
4.05457E-07
7.12332E-07
2.33004E-07
2.87618E-07
3.61251E-07
4.23735E-06
3.52316E-07
2.65961E-07
2.26716E-07
5.86221E-06
3.89793E-07
8.35146E-05
1.36352E-07
1.70176E-07
1.17740E-07

1.30987E-07
1.61760E-07
1.58859E-07
1.72866E-07
7.44768E-07
1.46428E-07
1.45507E-07
1.37459E-07
1.70034E-07
1.02387E-06
6.31331E-06
1.99552E-07
2.18631E-07
1.85246E-07
1.66070E-07
1.74972E-07
1.35942E-07
1.30395E-07
2.15543E-05
1.17874E-07
1.19193E-07
7.78324E-08
1.28167E-07
3.03940E-07
1.31728E-07
4.34303E-07
7.34660E-06
1.13251E-07
3.59074E-07
1.24695E-07
1.48905E-07
1.81397E-07
1.01315E-07
9.72454E-08
1.58833E-07
2.53895E-07
1.40615E-06
2.00804E-07
1.47237E-07
2.05402E-07
3.84874E-04
1.50663E-07
8.07119E-08
6.13710E-08
9.63096E-08
6.66384E-08
7.40473E-08
6.60446E-08
7.62425E-08
1.01611E-07
1.38966E-07
9.48407E-08
3.39605E-07
8.64873E-08

9.37004E-07
1.11309E-07
1.08157E-07
5.49574E-08
1.36566E-07
1.24226E-07
8.21539E-08
8.31274E-08
3.61385E-05
3.05351E-05
1.01993E-07
1.21637E-07
1.24433E-07
6.86946E-08
1.01866E-05
1.00851E-07
8.21427E-08
6.24327E-05
8.49025E-08
8.57662E-08
1.19898E-07
1.29555E-07
1.08973E-07
8.49511E-08
3.02816E-04
4.73268E-08
3.35287E-07
3.02183E-08
4.54403E-08
4.52810E-08
5.20807E-08
3.45191E-08
1.00000E-08
3.99999E-08
3.42277E-08
6.77703E-08
2.59514E-08
7.12884E-08
1.07426E-06
7.01957E-08
5.61336E-08
3.26381E-08
3.54403E-08
2.58806E-08
6.82524E-08
3.74514E-08
3.02201E-08
3.33374E-08
3.50309E-08
1.52202E-08
1.02201E-08
2.00000E-08
2.27092E-08
4.54402E-08

2.57183E-08
3.13677E-08
3.73175E-08
1.25887E-07
3.10310E-08
3.39595E-08
5.00000E-09
3.11871E-07
3.89205E-08
1.72540E-08
2.85024E-08
3.76659E-08
2.02356E-08
1.77251E-08
2.53592E-08
2.03820E-08
3.05984E-08
1.20946E-08
2.00000E-08
3.86395E-08
2.52201E-08
3.06731E-08
8.60012E-08
1.48430E-07
4.13313E-08
1.14039E-08
1.52202E-08
3.92215E-07
1.01409E-07
1.69533E-08
3.56603E-08
2.59627E-08
1.06240E-08
2.07273E-08
9.99996E-09
1.20837E-08
3.04403E-08
1.52201E-08
7.07000E-06
3.78710E-07
2.56604E-08
1.67288E-08
5.36172E-07
6.09550E-08
6.01092E-07
4.88635E-08
6.99985E-08
6.16612E-08
9.98993E-05
3.00937E-08
3.00000E-08
2.50000E-08
2.52201E-08
3.50000E-08

2.50000E-08
1.52201E-08
4.19727E-08
1.63313E-08
1.63314E-08
9.99996E-09
0.00000E+00
2.06646E-08
1.52202E-08
2.02201E-08
1.49999E-08
0.00000E+00
1.04403E-08
9.99996E-09
2.04402E-08
5.22015E-09
1.52201E-08
1.56605E-08
5.22015E-09
4.99996E-09
2.04403E-08
1.02201E-08
1.50000E-08
5.00000E-09
3.10220E-07
2.36455E-06
8.06604E-08
8.74321E-08
1.49999E-08
5.00000E-09
3.02201E-08
1.52201E-08
3.52201E-08
4.99996E-09
5.00000E-09
5.22015E-09
1.22500E-06
1.52201E-08
1.49999E-08
2.02202E-08
5.00000E-09
2.49999E-08
1.00976E-08
1.50000E-08
0.00000E+00
4.99996E-09
1.50000E-08
1.56605E-08
1.49999E-08
2.02201E-08
9.99996E-09
2.00000E-08
1.99999E-08
1.02202E-08

1.49999E-08
4.99996E-09
1.02202E-08
5.22015E-09
1.99999E-08
1.50000E-08
7.27586E-08
5.22015E-09
2.02201E-08
1.00000E-08
1.52012E-08
1.52201E-08
4.35500E-06
5.00000E-09
1.00000E-08
1.02202E-08
0.00000E+00
1.02202E-08
1.50000E-08
5.00000E-09
5.22015E-09
5.22015E-09
1.52201E-08
0.00000E+00
9.99996E-09
0.00000E+00
5.00000E-09
1.02201E-08
1.54403E-08
2.56604E-08
5.00000E-09
0.00000E+00
1.02201E-08
5.00000E-09
1.52201E-08
4.99996E-09
4.99998E-09
2.04402E-08
1.00000E-08
2.02201E-08
1.00000E-08
1.00000E-08
2.02202E-08
2.55466E-08
1.55000E-07
1.02202E-08
1.02201E-08
4.99996E-09
1.50000E-08
4.99996E-09
1.00000E-08
1.52201E-08
5.00000E-09
5.00000E-09

5.18340E-07
1.02201E-08
5.00000E-09
1.56605E-08
5.00000E-09
3.52201E-08
1.00000E-08
3.06604E-08
1.50000E-07
2.04403E-08
0.00000E+00
5.00000E-09
2.52201E-08
5.00000E-09
9.99994E-09
5.00000E-09
2.54402E-08
2.54403E-08
5.00000E-09
5.00000E-09
1.00000E-08
1.52201E-08
5.00000E-09
2.24515E-08
2.52201E-08
1.52201E-08
9.99996E-09
9.99996E-09
1.00000E-08
1.00000E-08
1.02201E-08
0.00000E+00
4.99996E-09
7.53339E-08
5.22015E-09
9.99996E-09
0.00000E+00
3.06604E-08
5.22015E-09
5.00000E-09
1.02201E-08
0.00000E+00
1.02201E-08
5.00000E-09
5.22015E-09
2.00000E-08
1.00000E-08
1.54403E-08
1.02201E-08
3.00000E-08
9.99996E-09
5.00000E-09
1.52201E-08
5.22015E-09

1.50000E-08
1.50000E-08
1.00000E-08
1.02201E-08
9.99996E-09
1.02201E-08
5.00000E-09
0.00000E+00
0.00000E+00
2.56605E-08
1.50000E-08
5.00000E-09
1.02201E-08
1.50000E-08
2.00000E-08
0.00000E+00
1.54403E-08
1.00000E-08
0.00000E+00
1.00000E-08
1.00000E-08
1.99999E-08
5.00000E-09
0.00000E+00
5.00000E-09
5.00000E-09
1.00000E-08
5.00000E-09
5.00000E-09
1.52201E-08
1.02201E-08
5.00000E-09
5.00000E-09
1.00000E-08
2.49999E-08
8.15000E-07
4.99998E-09
1.00000E-08
1.52201E-08
5.00000E-09
5.00000E-09
0.00000E+00
0.00000E+00
5.22015E-09
0.00000E+00
2.00000E-08
1.00000E-08
0.00000E+00
1.02201E-08
2.02201E-08
1.99999E-08
5.00000E-09
1.50000E-08
1.50000E-08

0.00000E+00
5.00000E-09
4.99996E-09
2.95000E-07
1.50000E-08
1.50000E-08
1.02201E-08
5.22015E-09
5.00000E-09
0.00000E+00
2.52201E-08
0.00000E+00
5.00000E-09
5.22015E-09
0.00000E+00
0.00000E+00
0.00000E+00
1.00000E-08
1.00000E-08
5.22015E-09
0.00000E+00
0.00000E+00
0.00000E+00
1.00000E-08
4.99996E-09
5.00000E-09
1.02201E-08
0.00000E+00
5.00000E-09
0.00000E+00
0.00000E+00
0.00000E+00
1.50000E-08
1.02201E-08
1.00000E-08
1.02201E-08
5.00000E-09
1.00000E-08
9.99996E-09
2.00000E-08
1.50000E-08
1.52201E-08
0.00000E+00
0.00000E+00
1.50000E-08
1.50000E-08
0.00000E+00
0.00000E+00
0.00000E+00
0.00000E+00
5.00000E-09
0.00000E+00
1.00000E-08
1.00000E-08

0.00000E+00
1.00000E-08
5.00000E-09
5.00000E-09
0.00000E+00
0.00000E+00
1.00000E-08
1.50000E-08
1.00000E-08
1.00000E-08
1.52201E-08
5.00000E-09
1.02201E-08
1.50000E-08
0.00000E+00
5.00000E-09
5.00000E-09
1.00000E-08
0.00000E+00
0.00000E+00
5.00000E-09
5.00000E-09
0.00000E+00
0.00000E+00
1.02201E-08
1.00000E-08
5.00000E-09
0.00000E+00
1.00000E-08
0.00000E+00
5.00000E-09
0.00000E+00
0.00000E+00
1.00000E-08
0.00000E+00
0.00000E+00
0.00000E+00
0.00000E+00
0.00000E+00
0.00000E+00
0.00000E+00
0.00000E+00
5.00000E-09
5.00000E-09
1.00000E-08
1.50000E-08
5.00000E-09
0.00000E+00
0.00000E+00
0.00000E+00
5.00000E-09
0.00000E+00
4.99996E-09
5.22015E-09

```
     0.00000E+00
     1.00000E-08
     0.00000E+00
     5.00000E-09
     0.00000E+00
     0.00000E+00
     1.50000E-08
     0.00000E+00
     1.00000E-08
     1.00000E-08
     0.00000E+00
     0.00000E+00
     0.00000E+00
cut:p j 0.001                      $ Photon cut-off energy 1.0 keV
f8:p  1                            $ Pulse height tally defined for cell
1 (NaI detector)
e8    0.0 1.0e-5 0.01 1198i 12.0    $ Energy binning
ft8   geb  0.016410  0.076003  0.0  $ Tally treatment with GEB
nps   2.0e+8                       $ Number of histories
```

4. SiO2 + 150 ppm Gd / GEB / 5 cm seawater layer

```
c     SiO2 + 150 ppm Gd
c     Pulse 'OFF'
c     Using re-normalized f1 data
c     Particle weight determined from the total of f1 data
c
c
c   Problem Cells
c
1  1 -3.67      1  -2  -3    imp:p=1     $ crystal NaI
2  2 -1.22e-3  -4  #1        imp:p=1     $ Inner world (air)
3  0            4            imp:p=0     $ Outer world (void)

c
c   Problem Surfaces
c
1  pz  0.0
2  pz  7.62
3  cz  3.81
4  so  100.0

c
c   Problem data cards
c
mode p                      $ photon only problem
c
c  NaI
c
m1   11023.80c   1
     53127.80c   1
c
c  Air
c
m2    7014.80c   0.8
      8016.80c   0.2
c
c   Source definition -- energy is now a prob. dist.
c
sdef erg=d2  rad=d1  pos=0.0 0.0 -3.0  vec=0 0 1  dir=1  ext=0
     axs=0 0 1 wgt=0.0514784
si1   0  3.81    $ radial sampling range: 0 to 3.81
sp1  -21 1       $ uniform sampling over the disk area
si2 h 0.000e+00  $ source energy binning / histogram
      1.000e-02
      2.000e-02
      3.000e-02
      4.000e-02
      5.000e-02
      6.000e-02
      7.000e-02
      8.000e-02
      9.000e-02
      1.000e-01
      1.100e-01
```

1.200e-01
1.300e-01
1.400e-01
1.500e-01
1.600e-01
1.700e-01
1.800e-01
1.900e-01
2.000e-01
2.100e-01
2.200e-01
2.300e-01
2.400e-01
2.500e-01
2.600e-01
2.700e-01
2.800e-01
2.900e-01
3.000e-01
3.100e-01
3.200e-01
3.300e-01
3.400e-01
3.500e-01
3.600e-01
3.700e-01
3.800e-01
3.900e-01
4.000e-01
4.100e-01
4.200e-01
4.300e-01
4.400e-01
4.500e-01
4.600e-01
4.700e-01
4.800e-01
4.900e-01
5.000e-01
5.100e-01
5.200e-01
5.300e-01
5.400e-01
5.500e-01
5.600e-01
5.700e-01
5.800e-01
5.900e-01
6.000e-01
6.100e-01
6.200e-01
6.300e-01
6.400e-01
6.500e-01

```
6.600e-01
6.700e-01
6.800e-01
6.900e-01
7.000e-01
7.100e-01
7.200e-01
7.300e-01
7.400e-01
7.500e-01
7.600e-01
7.700e-01
7.800e-01
7.900e-01
8.000e-01
8.100e-01
8.200e-01
8.300e-01
8.400e-01
8.500e-01
8.600e-01
8.700e-01
8.800e-01
8.900e-01
9.000e-01
9.100e-01
9.200e-01
9.300e-01
9.400e-01
9.500e-01
9.600e-01
9.700e-01
9.800e-01
9.900e-01
1.000e+00
1.010e+00
1.020e+00
1.030e+00
1.040e+00
1.050e+00
1.060e+00
1.070e+00
1.080e+00
1.090e+00
1.100e+00
1.110e+00
1.120e+00
1.130e+00
1.140e+00
1.150e+00
1.160e+00
1.170e+00
1.180e+00
1.190e+00
```

1.200e+00
1.210e+00
1.220e+00
1.230e+00
1.240e+00
1.250e+00
1.260e+00
1.270e+00
1.280e+00
1.290e+00
1.300e+00
1.310e+00
1.320e+00
1.330e+00
1.340e+00
1.350e+00
1.360e+00
1.370e+00
1.380e+00
1.390e+00
1.400e+00
1.410e+00
1.420e+00
1.430e+00
1.440e+00
1.450e+00
1.460e+00
1.470e+00
1.480e+00
1.490e+00
1.500e+00
1.510e+00
1.520e+00
1.530e+00
1.540e+00
1.550e+00
1.560e+00
1.570e+00
1.580e+00
1.590e+00
1.600e+00
1.610e+00
1.620e+00
1.630e+00
1.640e+00
1.650e+00
1.660e+00
1.670e+00
1.680e+00
1.690e+00
1.700e+00
1.710e+00
1.720e+00
1.730e+00

1.740e+00
1.750e+00
1.760e+00
1.770e+00
1.780e+00
1.790e+00
1.800e+00
1.810e+00
1.820e+00
1.830e+00
1.840e+00
1.850e+00
1.860e+00
1.870e+00
1.880e+00
1.890e+00
1.900e+00
1.910e+00
1.920e+00
1.930e+00
1.940e+00
1.950e+00
1.960e+00
1.970e+00
1.980e+00
1.990e+00
2.000e+00
2.010e+00
2.020e+00
2.030e+00
2.040e+00
2.050e+00
2.060e+00
2.070e+00
2.080e+00
2.090e+00
2.100e+00
2.110e+00
2.120e+00
2.130e+00
2.140e+00
2.150e+00
2.160e+00
2.170e+00
2.180e+00
2.190e+00
2.200e+00
2.210e+00
2.220e+00
2.230e+00
2.240e+00
2.250e+00
2.260e+00
2.270e+00

2.280e+00
2.290e+00
2.300e+00
2.310e+00
2.320e+00
2.330e+00
2.340e+00
2.350e+00
2.360e+00
2.370e+00
2.380e+00
2.390e+00
2.400e+00
2.410e+00
2.420e+00
2.430e+00
2.440e+00
2.450e+00
2.460e+00
2.470e+00
2.480e+00
2.490e+00
2.500e+00
2.510e+00
2.520e+00
2.530e+00
2.540e+00
2.550e+00
2.560e+00
2.570e+00
2.580e+00
2.590e+00
2.600e+00
2.610e+00
2.620e+00
2.630e+00
2.640e+00
2.650e+00
2.660e+00
2.670e+00
2.680e+00
2.690e+00
2.700e+00
2.710e+00
2.720e+00
2.730e+00
2.740e+00
2.750e+00
2.760e+00
2.770e+00
2.780e+00
2.790e+00
2.800e+00
2.810e+00

2.820e+00
2.830e+00
2.840e+00
2.850e+00
2.860e+00
2.870e+00
2.880e+00
2.890e+00
2.900e+00
2.910e+00
2.920e+00
2.930e+00
2.940e+00
2.950e+00
2.960e+00
2.970e+00
2.980e+00
2.990e+00
3.000e+00
3.010e+00
3.020e+00
3.030e+00
3.040e+00
3.050e+00
3.060e+00
3.070e+00
3.080e+00
3.090e+00
3.100e+00
3.110e+00
3.120e+00
3.130e+00
3.140e+00
3.150e+00
3.160e+00
3.170e+00
3.180e+00
3.190e+00
3.200e+00
3.210e+00
3.220e+00
3.230e+00
3.240e+00
3.250e+00
3.260e+00
3.270e+00
3.280e+00
3.290e+00
3.300e+00
3.310e+00
3.320e+00
3.330e+00
3.340e+00
3.350e+00

3.360e+00
3.370e+00
3.380e+00
3.390e+00
3.400e+00
3.410e+00
3.420e+00
3.430e+00
3.440e+00
3.450e+00
3.460e+00
3.470e+00
3.480e+00
3.490e+00
3.500e+00
3.510e+00
3.520e+00
3.530e+00
3.540e+00
3.550e+00
3.560e+00
3.570e+00
3.580e+00
3.590e+00
3.600e+00
3.610e+00
3.620e+00
3.630e+00
3.640e+00
3.650e+00
3.660e+00
3.670e+00
3.680e+00
3.690e+00
3.700e+00
3.710e+00
3.720e+00
3.730e+00
3.740e+00
3.750e+00
3.760e+00
3.770e+00
3.780e+00
3.790e+00
3.800e+00
3.810e+00
3.820e+00
3.830e+00
3.840e+00
3.850e+00
3.860e+00
3.870e+00
3.880e+00
3.890e+00

3.900e+00
3.910e+00
3.920e+00
3.930e+00
3.940e+00
3.950e+00
3.960e+00
3.970e+00
3.980e+00
3.990e+00
4.000e+00
4.010e+00
4.020e+00
4.030e+00
4.040e+00
4.050e+00
4.060e+00
4.070e+00
4.080e+00
4.090e+00
4.100e+00
4.110e+00
4.120e+00
4.130e+00
4.140e+00
4.150e+00
4.160e+00
4.170e+00
4.180e+00
4.190e+00
4.200e+00
4.210e+00
4.220e+00
4.230e+00
4.240e+00
4.250e+00
4.260e+00
4.270e+00
4.280e+00
4.290e+00
4.300e+00
4.310e+00
4.320e+00
4.330e+00
4.340e+00
4.350e+00
4.360e+00
4.370e+00
4.380e+00
4.390e+00
4.400e+00
4.410e+00
4.420e+00
4.430e+00

4.440e+00
4.450e+00
4.460e+00
4.470e+00
4.480e+00
4.490e+00
4.500e+00
4.510e+00
4.520e+00
4.530e+00
4.540e+00
4.550e+00
4.560e+00
4.570e+00
4.580e+00
4.590e+00
4.600e+00
4.610e+00
4.620e+00
4.630e+00
4.640e+00
4.650e+00
4.660e+00
4.670e+00
4.680e+00
4.690e+00
4.700e+00
4.710e+00
4.720e+00
4.730e+00
4.740e+00
4.750e+00
4.760e+00
4.770e+00
4.780e+00
4.790e+00
4.800e+00
4.810e+00
4.820e+00
4.830e+00
4.840e+00
4.850e+00
4.860e+00
4.870e+00
4.880e+00
4.890e+00
4.900e+00
4.910e+00
4.920e+00
4.930e+00
4.940e+00
4.950e+00
4.960e+00
4.970e+00

4.980e+00
4.990e+00
5.000e+00
5.010e+00
5.020e+00
5.030e+00
5.040e+00
5.050e+00
5.060e+00
5.070e+00
5.080e+00
5.090e+00
5.100e+00
5.110e+00
5.120e+00
5.130e+00
5.140e+00
5.150e+00
5.160e+00
5.170e+00
5.180e+00
5.190e+00
5.200e+00
5.210e+00
5.220e+00
5.230e+00
5.240e+00
5.250e+00
5.260e+00
5.270e+00
5.280e+00
5.290e+00
5.300e+00
5.310e+00
5.320e+00
5.330e+00
5.340e+00
5.350e+00
5.360e+00
5.370e+00
5.380e+00
5.390e+00
5.400e+00
5.410e+00
5.420e+00
5.430e+00
5.440e+00
5.450e+00
5.460e+00
5.470e+00
5.480e+00
5.490e+00
5.500e+00
5.510e+00

5.520e+00
5.530e+00
5.540e+00
5.550e+00
5.560e+00
5.570e+00
5.580e+00
5.590e+00
5.600e+00
5.610e+00
5.620e+00
5.630e+00
5.640e+00
5.650e+00
5.660e+00
5.670e+00
5.680e+00
5.690e+00
5.700e+00
5.710e+00
5.720e+00
5.730e+00
5.740e+00
5.750e+00
5.760e+00
5.770e+00
5.780e+00
5.790e+00
5.800e+00
5.810e+00
5.820e+00
5.830e+00
5.840e+00
5.850e+00
5.860e+00
5.870e+00
5.880e+00
5.890e+00
5.900e+00
5.910e+00
5.920e+00
5.930e+00
5.940e+00
5.950e+00
5.960e+00
5.970e+00
5.980e+00
5.990e+00
6.000e+00
6.010e+00
6.020e+00
6.030e+00
6.040e+00
6.050e+00

6.060e+00
6.070e+00
6.080e+00
6.090e+00
6.100e+00
6.110e+00
6.120e+00
6.130e+00
6.140e+00
6.150e+00
6.160e+00
6.170e+00
6.180e+00
6.190e+00
6.200e+00
6.210e+00
6.220e+00
6.230e+00
6.240e+00
6.250e+00
6.260e+00
6.270e+00
6.280e+00
6.290e+00
6.300e+00
6.310e+00
6.320e+00
6.330e+00
6.340e+00
6.350e+00
6.360e+00
6.370e+00
6.380e+00
6.390e+00
6.400e+00
6.410e+00
6.420e+00
6.430e+00
6.440e+00
6.450e+00
6.460e+00
6.470e+00
6.480e+00
6.490e+00
6.500e+00
6.510e+00
6.520e+00
6.530e+00
6.540e+00
6.550e+00
6.560e+00
6.570e+00
6.580e+00
6.590e+00

6.600e+00
6.610e+00
6.620e+00
6.630e+00
6.640e+00
6.650e+00
6.660e+00
6.670e+00
6.680e+00
6.690e+00
6.700e+00
6.710e+00
6.720e+00
6.730e+00
6.740e+00
6.750e+00
6.760e+00
6.770e+00
6.780e+00
6.790e+00
6.800e+00
6.810e+00
6.820e+00
6.830e+00
6.840e+00
6.850e+00
6.860e+00
6.870e+00
6.880e+00
6.890e+00
6.900e+00
6.910e+00
6.920e+00
6.930e+00
6.940e+00
6.950e+00
6.960e+00
6.970e+00
6.980e+00
6.990e+00
7.000e+00
7.010e+00
7.020e+00
7.030e+00
7.040e+00
7.050e+00
7.060e+00
7.070e+00
7.080e+00
7.090e+00
7.100e+00
7.110e+00
7.120e+00
7.130e+00

7.140e+00
7.150e+00
7.160e+00
7.170e+00
7.180e+00
7.190e+00
7.200e+00
7.210e+00
7.220e+00
7.230e+00
7.240e+00
7.250e+00
7.260e+00
7.270e+00
7.280e+00
7.290e+00
7.300e+00
7.310e+00
7.320e+00
7.330e+00
7.340e+00
7.350e+00
7.360e+00
7.370e+00
7.380e+00
7.390e+00
7.400e+00
7.410e+00
7.420e+00
7.430e+00
7.440e+00
7.450e+00
7.460e+00
7.470e+00
7.480e+00
7.490e+00
7.500e+00
7.510e+00
7.520e+00
7.530e+00
7.540e+00
7.550e+00
7.560e+00
7.570e+00
7.580e+00
7.590e+00
7.600e+00
7.610e+00
7.620e+00
7.630e+00
7.640e+00
7.650e+00
7.660e+00
7.670e+00

7.680e+00
7.690e+00
7.700e+00
7.710e+00
7.720e+00
7.730e+00
7.740e+00
7.750e+00
7.760e+00
7.770e+00
7.780e+00
7.790e+00
7.800e+00
7.810e+00
7.820e+00
7.830e+00
7.840e+00
7.850e+00
7.860e+00
7.870e+00
7.880e+00
7.890e+00
7.900e+00
7.910e+00
7.920e+00
7.930e+00
7.940e+00
7.950e+00
7.960e+00
7.970e+00
7.980e+00
7.990e+00
8.000e+00
8.010e+00
8.020e+00
8.030e+00
8.040e+00
8.050e+00
8.060e+00
8.070e+00
8.080e+00
8.090e+00
8.100e+00
8.110e+00
8.120e+00
8.130e+00
8.140e+00
8.150e+00
8.160e+00
8.170e+00
8.180e+00
8.190e+00
8.200e+00
8.210e+00

8.220e+00
8.230e+00
8.240e+00
8.250e+00
8.260e+00
8.270e+00
8.280e+00
8.290e+00
8.300e+00
8.310e+00
8.320e+00
8.330e+00
8.340e+00
8.350e+00
8.360e+00
8.370e+00
8.380e+00
8.390e+00
8.400e+00
8.410e+00
8.420e+00
8.430e+00
8.440e+00
8.450e+00
8.460e+00
8.470e+00
8.480e+00
8.490e+00
8.500e+00
8.510e+00
8.520e+00
8.530e+00
8.540e+00
8.550e+00
8.560e+00
8.570e+00
8.580e+00
8.590e+00
8.600e+00
8.610e+00
8.620e+00
8.630e+00
8.640e+00
8.650e+00
8.660e+00
8.670e+00
8.680e+00
8.690e+00
8.700e+00
8.710e+00
8.720e+00
8.730e+00
8.740e+00
8.750e+00

8.760e+00
8.770e+00
8.780e+00
8.790e+00
8.800e+00
8.810e+00
8.820e+00
8.830e+00
8.840e+00
8.850e+00
8.860e+00
8.870e+00
8.880e+00
8.890e+00
8.900e+00
8.910e+00
8.920e+00
8.930e+00
8.940e+00
8.950e+00
8.960e+00
8.970e+00
8.980e+00
8.990e+00
9.000e+00
9.010e+00
9.020e+00
9.030e+00
9.040e+00
9.050e+00
9.060e+00
9.070e+00
9.080e+00
9.090e+00
9.100e+00
9.110e+00
9.120e+00
9.130e+00
9.140e+00
9.150e+00
9.160e+00
9.170e+00
9.180e+00
9.190e+00
9.200e+00
9.210e+00
9.220e+00
9.230e+00
9.240e+00
9.250e+00
9.260e+00
9.270e+00
9.280e+00
9.290e+00

9.300e+00
9.310e+00
9.320e+00
9.330e+00
9.340e+00
9.350e+00
9.360e+00
9.370e+00
9.380e+00
9.390e+00
9.400e+00
9.410e+00
9.420e+00
9.430e+00
9.440e+00
9.450e+00
9.460e+00
9.470e+00
9.480e+00
9.490e+00
9.500e+00
9.510e+00
9.520e+00
9.530e+00
9.540e+00
9.550e+00
9.560e+00
9.570e+00
9.580e+00
9.590e+00
9.600e+00
9.610e+00
9.620e+00
9.630e+00
9.640e+00
9.650e+00
9.660e+00
9.670e+00
9.680e+00
9.690e+00
9.700e+00
9.710e+00
9.720e+00
9.730e+00
9.740e+00
9.750e+00
9.760e+00
9.770e+00
9.780e+00
9.790e+00
9.800e+00
9.810e+00
9.820e+00
9.830e+00

9.840e+00
9.850e+00
9.860e+00
9.870e+00
9.880e+00
9.890e+00
9.900e+00
9.910e+00
9.920e+00
9.930e+00
9.940e+00
9.950e+00
9.960e+00
9.970e+00
9.980e+00
9.990e+00
1.000e+01
1.001e+01
1.002e+01
1.003e+01
1.004e+01
1.005e+01
1.006e+01
1.007e+01
1.008e+01
1.009e+01
1.010e+01
1.011e+01
1.012e+01
1.013e+01
1.014e+01
1.015e+01
1.016e+01
1.017e+01
1.018e+01
1.019e+01
1.020e+01
1.021e+01
1.022e+01
1.023e+01
1.024e+01
1.025e+01
1.026e+01
1.027e+01
1.028e+01
1.029e+01
1.030e+01
1.031e+01
1.032e+01
1.033e+01
1.034e+01
1.035e+01
1.036e+01
1.037e+01

1.038e+01
1.039e+01
1.040e+01
1.041e+01
1.042e+01
1.043e+01
1.044e+01
1.045e+01
1.046e+01
1.047e+01
1.048e+01
1.049e+01
1.050e+01
1.051e+01
1.052e+01
1.053e+01
1.054e+01
1.055e+01
1.056e+01
1.057e+01
1.058e+01
1.059e+01
1.060e+01
1.061e+01
1.062e+01
1.063e+01
1.064e+01
1.065e+01
1.066e+01
1.067e+01
1.068e+01
1.069e+01
1.070e+01
1.071e+01
1.072e+01
1.073e+01
1.074e+01
1.075e+01
1.076e+01
1.077e+01
1.078e+01
1.079e+01
1.080e+01
1.081e+01
1.082e+01
1.083e+01
1.084e+01
1.085e+01
1.086e+01
1.087e+01
1.088e+01
1.089e+01
1.090e+01
1.091e+01

1.092e+01
1.093e+01
1.094e+01
1.095e+01
1.096e+01
1.097e+01
1.098e+01
1.099e+01
1.100e+01
1.101e+01
1.102e+01
1.103e+01
1.104e+01
1.105e+01
1.106e+01
1.107e+01
1.108e+01
1.109e+01
1.110e+01
1.111e+01
1.112e+01
1.113e+01
1.114e+01
1.115e+01
1.116e+01
1.117e+01
1.118e+01
1.119e+01
1.120e+01
1.121e+01
1.122e+01
1.123e+01
1.124e+01
1.125e+01
1.126e+01
1.127e+01
1.128e+01
1.129e+01
1.130e+01
1.131e+01
1.132e+01
1.133e+01
1.134e+01
1.135e+01
1.136e+01
1.137e+01
1.138e+01
1.139e+01
1.140e+01
1.141e+01
1.142e+01
1.143e+01
1.144e+01
1.145e+01

1.146e+01
1.147e+01
1.148e+01
1.149e+01
1.150e+01
1.151e+01
1.152e+01
1.153e+01
1.154e+01
1.155e+01
1.156e+01
1.157e+01
1.158e+01
1.159e+01
1.160e+01
1.161e+01
1.162e+01
1.163e+01
1.164e+01
1.165e+01
1.166e+01
1.167e+01
1.168e+01
1.169e+01
1.170e+01
1.171e+01
1.172e+01
1.173e+01
1.174e+01
1.175e+01
1.176e+01
1.177e+01
1.178e+01
1.179e+01
1.180e+01
1.181e+01
1.182e+01
1.183e+01
1.184e+01
1.185e+01
1.186e+01
1.187e+01
1.188e+01
1.189e+01
1.190e+01
1.191e+01
1.192e+01
1.193e+01
1.194e+01
1.195e+01
1.196e+01
1.197e+01
1.198e+01
1.199e+01

```
      1.200e+01
sp2  0.00000e+00  $ source energy bin probabilities
     1.57483E-06
     1.17924E-05
     3.48567E-05
     2.64352E-04
     6.80427E-04
     1.02591E-03
     1.17503E-03
     1.27023E-03
     1.15640E-03
     1.04462E-03
     9.55108E-04
     8.86599E-04
     8.27309E-04
     7.66436E-04
     7.06579E-04
     6.58301E-04
     6.14361E-04
     5.90388E-04
     5.51200E-04
     5.25978E-04
     4.98940E-04
     4.77517E-04
     4.62516E-04
     4.69975E-04
     4.38475E-04
     4.12248E-04
     3.77737E-04
     3.52939E-04
     3.27737E-04
     3.10187E-04
     2.86325E-04
     2.69519E-04
     2.52237E-04
     2.40977E-04
     2.28205E-04
     2.31416E-04
     2.10916E-04
     2.02018E-04
     1.93488E-04
     1.86247E-04
     1.82411E-04
     1.73827E-04
     1.70391E-04
     1.89230E-04
     1.60259E-04
     1.56689E-04
     1.58082E-04
     1.75902E-04
     1.43247E-04
     1.42247E-04
     1.47502E-04
     1.32471E-03
```

1.05419E-04
1.05324E-04
9.95345E-05
9.94995E-05
9.77464E-05
9.32850E-05
9.34404E-05
9.04247E-05
8.95312E-05
8.88123E-05
8.73664E-05
9.25914E-05
8.45468E-05
8.34587E-05
8.50513E-05
8.07948E-05
7.89400E-05
8.21444E-05
8.03251E-05
7.69679E-05
7.60203E-05
7.49110E-05
7.31349E-05
7.61414E-05
7.29011E-05
7.28568E-05
7.23622E-04
6.51703E-05
6.33998E-05
6.72990E-05
6.36957E-05
6.98989E-05
6.39403E-05
6.39389E-05
7.23910E-05
6.59491E-05
6.06538E-05
6.21590E-05
6.15225E-05
6.54802E-05
6.68719E-05
8.39489E-05
7.76795E-05
7.77493E-05
7.83807E-05
8.16001E-05
8.35421E-05
7.64285E-05
7.19139E-05
7.66248E-05
6.79014E-05
7.51735E-05
6.54345E-05
5.94084E-05

6.05455E-05
5.79547E-05
6.04066E-05
5.50986E-05
5.30958E-05
5.55246E-05
5.33548E-05
1.03218E-04
5.29683E-05
5.21576E-05
8.39269E-04
6.50126E-05
5.24418E-05
5.23912E-05
5.60795E-05
5.54267E-05
5.51503E-05
5.76248E-05
5.33530E-05
5.42447E-05
5.88194E-05
7.24787E-05
5.28765E-05
4.72368E-05
4.86397E-05
4.56635E-05
7.98612E-05
4.48496E-05
4.71357E-05
4.61398E-05
4.31563E-05
5.40430E-05
4.53015E-05
4.27212E-05
4.93718E-05
4.52080E-05
4.63072E-05
4.62710E-05
4.80248E-05
4.69770E-05
4.74423E-05
4.54092E-05
4.65757E-05
5.08065E-05
4.54347E-05
5.21457E-05
5.39578E-05
4.16185E-05
4.06145E-05
4.11602E-05
4.27913E-05
3.95969E-05
4.03734E-05
4.08705E-05

1.41466E-04
4.46822E-05
5.65167E-05
4.27386E-05
6.76531E-05
4.60629E-05
4.08828E-05
4.59922E-05
4.36182E-05
4.01639E-05
4.46417E-05
3.96813E-05
5.59526E-05
4.49658E-05
4.58154E-05
3.79357E-05
3.83727E-05
4.64060E-05
5.42618E-05
3.80129E-05
4.16686E-05
3.81240E-05
4.76883E-05
3.68906E-05
4.27454E-05
4.52485E-05
3.99428E-05
3.66602E-05
3.70810E-05
3.73803E-05
3.66430E-05
3.78664E-05
3.91372E-05
4.94396E-05
3.60263E-05
9.94939E-04
3.52192E-05
5.54348E-05
3.65647E-05
4.11821E-05
3.92477E-05
3.86571E-05
5.37095E-05
5.95954E-05
4.91566E-05
3.25575E-05
3.31056E-05
5.84469E-05
3.33358E-05
7.17784E-05
3.85903E-05
3.97439E-05
3.35268E-05
3.94905E-05

3.21943E-05
5.30069E-05
3.27925E-05
4.07097E-05
3.47198E-05
3.23625E-05
4.81206E-05
3.52506E-05
6.10827E-03
5.25318E-05
3.03640E-05
3.23523E-05
2.56479E-05
2.66849E-05
4.51076E-05
2.35344E-05
2.32967E-05
5.68325E-05
3.05878E-05
2.27632E-05
2.43472E-05
2.69539E-05
2.50466E-05
2.30626E-05
2.80312E-05
2.83665E-05
2.93698E-05
4.36463E-05
3.34566E-05
2.32709E-05
2.32572E-05
2.40609E-05
5.38306E-05
2.26607E-05
3.64797E-05
3.60507E-05
2.38149E-05
2.82584E-05
3.34583E-05
3.68919E-05
3.10890E-05
2.56720E-05
2.98732E-05
2.44871E-05
2.59820E-05
3.47232E-05
2.05683E-05
3.00378E-05
4.34953E-05
2.68024E-05
2.97874E-05
2.30749E-05
2.41583E-05
7.17755E-05

2.61153E-05
2.25948E-05
2.08279E-05
2.58392E-05
2.67099E-05
2.05197E-05
2.80005E-05
3.04751E-05
2.11412E-05
2.05327E-05
2.26841E-05
2.88933E-05
4.06822E-05
3.56559E-05
2.62781E-05
2.11471E-05
6.08490E-05
1.99120E-05
2.24674E-04
4.67321E-05
1.86820E-05
3.59405E-05
1.95295E-05
1.85362E-05
1.94074E-05
1.88662E-05
2.34568E-05
2.25611E-05
3.70084E-05
5.22359E-05
1.94995E-05
4.72360E-05
4.13567E-05
5.41002E-05
2.14517E-05
3.32926E-05
1.84865E-05
1.96119E-05
1.37087E-04
1.71985E-05
2.05712E-05
1.99008E-05
2.37489E-05
4.97941E-05
1.73516E-05
2.04578E-05
1.56273E-05
2.01152E-05
1.67945E-05
1.59417E-05
1.59256E-05
1.95590E-05
1.90868E-05
1.84783E-05

1.69758E-05
1.64060E-05
1.83655E-05
2.59260E-05
1.82761E-05
1.96927E-05
1.53228E-05
2.25018E-05
1.73775E-05
2.51938E-05
1.56581E-05
4.16610E-05
2.32190E-05
1.63560E-05
1.67138E-05
3.42219E-05
1.55107E-05
1.46864E-05
1.47155E-05
1.69394E-05
4.34026E-05
2.04270E-05
1.39016E-05
1.76219E-05
2.84364E-05
1.68668E-05
1.46996E-05
1.48186E-05
3.22066E-05
1.62522E-05
1.63672E-05
1.57602E-04
1.35302E-05
1.96859E-05
4.93249E-05
1.27859E-05
4.28739E-05
4.12762E-05
2.70393E-05
1.73479E-05
2.11173E-05
2.37328E-05
1.52193E-05
1.29240E-05
2.76748E-05
1.33237E-05
2.00389E-05
1.26019E-05
2.01631E-05
1.23849E-05
1.39365E-05
1.87525E-05
2.52019E-05
1.21825E-05

1.22009E-05
2.08424E-05
1.26800E-05
1.40432E-05
1.34719E-05
1.17237E-05
7.86089E-05
1.14877E-05
1.89083E-05
1.54379E-05
1.72467E-05
1.96886E-05
1.08579E-05
1.15480E-05
1.14457E-05
1.32782E-05
1.32168E-05
1.16714E-05
1.07428E-05
1.66490E-05
2.53849E-05
1.51601E-05
5.16116E-05
1.29855E-05
1.36177E-05
1.18187E-05
1.63967E-05
1.08335E-05
1.28830E-05
3.13821E-05
1.84415E-05
1.09091E-05
3.87330E-05
1.24273E-05
1.01262E-05
1.36980E-05
1.13815E-05
3.68624E-05
9.63301E-06
9.80513E-06
1.43702E-05
1.05519E-05
9.93514E-06
1.07290E-05
1.40690E-05
1.36331E-05
8.41550E-06
8.73675E-06
8.94386E-06
9.30972E-06
2.46569E-05
9.64153E-06
8.82850E-06
2.37143E-05

1.08365E-05
8.45050E-06
8.92327E-06
8.90064E-06
7.59038E-06
1.33864E-05
9.31111E-06
9.55495E-06
8.29277E-06
8.00426E-06
1.02227E-05
1.95691E-05
1.11068E-05
9.12009E-06
4.43436E-05
1.17276E-05
8.14082E-06
8.47331E-06
7.56290E-06
1.04290E-05
7.90618E-06
1.27540E-05
2.32811E-05
7.06557E-06
2.26373E-05
1.23593E-05
6.57862E-06
8.38927E-06
1.92595E-05
1.20675E-05
7.50221E-06
3.05041E-05
7.40862E-06
7.44683E-06
6.98805E-06
7.89041E-06
1.20338E-05
7.20724E-06
8.59584E-06
1.65607E-05
6.62826E-06
6.39153E-06
3.12903E-05
2.01741E-05
7.83173E-06
1.58689E-05
8.13967E-06
6.18242E-06
6.41328E-06
7.54120E-06
7.95978E-06
1.39152E-05
1.29004E-05
6.62236E-06

6.45631E-06
5.79972E-06
5.17639E-06
5.44210E-06
9.48059E-06
5.63571E-06
1.25872E-05
6.26801E-06
5.56080E-06
1.55417E-04
6.68592E-05
1.14141E-05
5.25425E-06
1.32182E-04
1.55965E-05
5.09862E-06
6.59575E-06
2.14036E-05
4.79722E-06
4.29400E-06
5.36487E-06
5.07878E-06
5.10861E-06
1.02462E-05
6.07182E-06
4.73599E-06
1.71947E-05
5.30320E-06
5.63374E-06
1.15348E-05
7.62475E-06
1.10819E-05
4.80298E-06
4.08568E-06
4.54543E-06
4.23584E-06
1.12412E-05
4.11771E-06
3.77344E-06
3.88943E-06
2.44553E-05
4.37670E-06
8.26201E-06
5.41030E-06
4.20452E-06
4.49101E-06
4.29647E-06
3.91360E-06
4.02162E-06
3.90059E-06
3.84288E-06
4.67102E-06
3.77293E-06
5.70198E-06

3.79624E-06
3.68030E-06
3.61388E-06
8.81634E-06
3.37084E-06
3.93731E-06
3.61317E-06
4.26203E-06
3.92336E-06
6.61667E-06
3.66117E-06
3.78777E-06
3.93556E-06
6.20889E-05
3.81910E-06
3.72758E-06
3.42372E-06
3.37908E-06
3.39759E-06
3.35396E-06
2.52053E-05
3.08030E-06
1.55730E-05
5.68635E-06
3.16111E-06
5.00277E-06
3.03489E-06
2.94172E-06
2.91247E-06
2.77757E-06
2.86030E-06
2.78681E-06
1.81145E-05
1.92745E-04
3.02425E-06
2.19233E-05
2.93026E-06
5.45480E-06
3.60537E-06
8.51436E-06
3.02733E-06
2.59912E-06
2.55276E-06
2.81681E-06
2.59086E-06
2.76569E-06
2.55584E-06
2.41176E-06
3.10170E-06
2.32558E-06
2.45291E-06
2.18243E-06
4.13407E-05
2.67489E-06

1.41676E-05
2.18915E-06
2.24773E-06
9.03166E-06
2.09773E-06
2.36481E-06
2.49095E-06
2.47563E-06
2.45538E-06
1.44366E-05
2.47995E-06
2.22591E-06
3.45487E-06
4.21838E-06
2.12912E-06
2.23082E-06
3.22628E-05
2.24004E-06
8.74880E-06
7.31849E-04
1.77399E-05
2.25710E-06
2.26259E-06
1.80184E-06
1.86173E-06
1.88570E-06
6.60199E-06
1.64572E-06
3.16554E-06
2.03288E-06
1.55789E-06
1.75716E-06
1.87231E-06
4.16871E-06
1.76628E-05
1.82946E-06
1.80369E-06
1.75062E-06
1.62932E-06
5.44746E-06
1.92702E-06
3.86786E-06
3.72894E-06
1.94019E-06
1.80942E-06
3.67164E-05
3.75664E-06
1.16067E-05
2.24425E-06
2.24483E-06
1.20274E-05
2.08263E-06
3.53453E-06
2.00629E-06

2.26864E-06
1.87806E-06
6.92758E-06
2.08382E-06
1.95559E-06
1.48893E-06
1.35652E-06
1.06037E-06
7.12924E-06
1.02431E-06
8.82523E-07
8.40316E-07
9.75460E-07
1.05368E-06
1.24324E-06
2.83640E-04
1.67844E-04
1.88398E-06
9.56844E-06
2.60038E-06
2.93492E-06
3.05423E-06
2.92117E-06
3.04708E-06
2.94427E-06
5.71824E-06
3.40616E-06
3.36195E-06
6.29778E-06
2.69337E-06
2.36206E-06
2.31906E-06
1.79333E-06
1.79587E-06
1.10861E-06
1.16888E-06
6.08243E-07
1.27070E-06
4.79724E-07
4.21008E-07
7.97681E-07
1.19826E-06
6.38595E-07
6.67443E-07
9.47226E-07
4.60745E-06
6.17865E-07
5.70832E-07
6.65283E-07
6.17053E-06
7.26922E-07
8.22813E-05
4.21871E-07
3.35439E-07

2.53792E-07
2.65993E-07
2.92775E-07
2.36049E-07
2.52726E-07
7.62885E-07
2.18426E-07
2.02346E-07
1.41337E-07
1.89737E-07
8.82854E-07
6.32799E-06
2.46069E-07
2.05236E-07
1.98504E-07
1.64078E-07
1.81438E-07
2.55842E-07
1.45642E-07
1.83285E-05
1.78019E-07
2.34862E-07
1.10453E-07
2.36580E-07
3.80864E-07
2.66977E-07
5.29371E-07
7.61035E-06
1.75090E-07
4.56562E-07
2.50215E-07
2.74450E-07
1.82924E-07
2.31091E-07
2.31539E-07
3.32919E-07
3.90752E-07
1.60859E-06
4.40036E-07
3.77437E-07
2.96409E-07
3.81166E-04
2.99332E-07
1.72274E-07
1.23080E-07
2.29290E-07
1.73687E-07
1.61506E-07
1.64274E-07
9.31118E-08
1.18520E-07
1.48761E-07
1.35223E-07
3.22901E-07

1.29933E-07
1.05180E-06
1.26705E-07
1.22327E-07
9.02839E-08
9.81316E-08
1.47957E-07
9.56876E-08
5.80487E-08
3.62207E-05
3.01564E-05
8.23714E-08
1.26798E-07
1.62396E-07
1.25632E-07
1.02180E-05
1.19350E-07
7.76092E-08
6.17913E-05
4.78835E-07
6.24185E-07
7.03761E-07
1.03852E-06
8.63915E-07
1.14383E-06
3.00729E-04
1.46670E-06
2.01806E-06
2.36872E-06
2.64308E-06
2.88993E-06
2.92457E-06
3.44434E-06
3.50146E-06
3.74915E-06
3.12496E-06
3.34305E-06
2.66506E-06
2.22866E-06
2.15652E-06
8.74928E-08
5.28687E-08
3.94352E-08
4.07569E-08
4.08806E-08
7.49081E-08
6.39061E-08
3.02201E-08
4.18204E-08
1.49999E-08
2.55951E-08
1.02201E-08
2.12773E-08
5.22015E-09

2.54402E-08
4.06567E-08
4.05732E-08
4.11852E-08
1.10000E-07
2.72576E-08
1.85192E-08
1.52202E-08
2.06871E-07
3.91407E-08
2.72540E-08
1.50000E-08
4.32327E-08
2.02356E-08
1.50000E-08
1.52201E-08
1.52201E-08
4.03782E-08
1.02575E-08
2.00000E-08
3.26689E-08
1.52201E-08
2.19383E-08
1.11460E-07
1.54550E-07
5.86104E-08
9.73212E-08
6.22364E-08
4.50107E-07
2.13020E-07
1.04094E-07
1.67948E-07
1.02314E-07
1.67678E-07
1.86470E-07
1.08936E-07
2.32026E-07
1.66454E-07
1.84092E-07
5.94132E-06
6.09259E-07
1.71821E-07
1.55162E-07
5.71264E-07
1.47731E-07
6.12212E-07
4.41605E-08
3.99985E-08
4.50000E-08
9.89069E-05
4.00937E-08
5.50000E-08
2.50000E-08
4.02201E-08

2.50000E-08
3.00000E-08
1.52201E-08
2.67526E-08
2.13313E-08
2.63314E-08
1.50000E-08
0.00000E+00
2.56646E-08
5.00000E-09
1.52201E-08
9.99992E-09
0.00000E+00
5.22015E-09
9.99996E-09
2.54402E-08
5.22015E-09
1.52201E-08
5.22015E-09
5.22015E-09
4.99996E-09
2.58806E-08
4.99996E-09
1.50000E-08
5.00000E-09
2.95220E-07
2.33584E-06
8.04402E-08
1.07652E-07
1.99999E-08
5.00000E-09
3.02201E-08
9.99996E-09
4.00000E-08
9.99996E-09
1.00000E-08
5.22015E-09
1.20500E-06
2.02201E-08
2.02201E-08
1.52202E-08
5.22015E-09
2.49999E-08
1.00000E-08
1.52201E-08
0.00000E+00
4.99996E-09
1.50000E-08
0.00000E+00
1.49999E-08
9.99996E-09
9.99996E-09
1.50000E-08
2.52201E-08

1.02202E-08
1.49999E-08
4.99996E-09
5.22015E-09
5.22015E-09
1.49999E-08
1.50000E-08
7.25835E-08
5.22015E-09
2.51968E-08
1.52201E-08
2.02012E-08
2.02201E-08
4.31500E-06
5.00000E-09
1.00000E-08
1.02202E-08
0.00000E+00
1.52202E-08
1.50000E-08
0.00000E+00
0.00000E+00
5.22015E-09
1.52201E-08
0.00000E+00
9.99994E-09
0.00000E+00
5.00000E-09
1.02201E-08
1.54403E-08
2.56604E-08
5.00000E-09
0.00000E+00
5.22015E-09
1.02202E-08
1.52201E-08
4.99996E-09
0.00000E+00
2.04402E-08
1.00000E-08
2.02201E-08
1.52201E-08
1.00000E-08
2.04403E-08
2.54403E-08
1.35000E-07
1.02202E-08
1.02201E-08
1.02201E-08
2.02201E-08
4.99996E-09
5.00000E-09
1.52201E-08
5.00000E-09

0.00000E+00
5.28361E-07
1.02201E-08
5.00000E-09
1.56605E-08
1.00000E-08
1.52201E-08
5.00000E-09
2.02201E-08
8.50000E-08
1.02201E-08
5.00000E-09
5.00000E-09
2.52201E-08
5.00000E-09
1.49999E-08
5.00000E-09
3.06604E-08
2.54403E-08
1.00000E-08
5.00000E-09
5.00000E-09
5.00000E-09
5.00000E-09
1.74515E-08
1.02201E-08
1.52201E-08
4.99996E-09
9.99996E-09
5.00000E-09
1.00000E-08
1.02201E-08
0.00000E+00
9.99994E-09
6.02094E-08
0.00000E+00
9.99996E-09
0.00000E+00
2.06604E-08
0.00000E+00
5.00000E-09
2.02201E-08
0.00000E+00
2.02201E-08
1.50000E-08
5.22015E-09
1.50000E-08
5.00000E-09
1.04403E-08
1.00000E-08
3.52201E-08
9.99996E-09
5.00000E-09
1.02201E-08

1.02201E-08
1.50000E-08
9.99996E-09
1.00000E-08
1.02201E-08
9.99996E-09
5.00000E-09
5.00000E-09
5.00000E-09
5.00000E-09
2.04403E-08
2.50000E-08
5.00000E-09
1.02201E-08
1.50000E-08
2.00000E-08
0.00000E+00
1.02201E-08
1.00000E-08
0.00000E+00
0.00000E+00
1.00000E-08
2.99999E-08
0.00000E+00
0.00000E+00
5.00000E-09
5.00000E-09
1.00000E-08
5.00000E-09
0.00000E+00
2.02201E-08
1.02201E-08
1.02201E-08
0.00000E+00
1.00000E-08
2.49999E-08
7.10000E-07
4.99998E-09
1.00000E-08
5.00000E-09
1.50000E-08
5.00000E-09
0.00000E+00
0.00000E+00
1.04403E-08
0.00000E+00
1.50000E-08
2.00000E-08
0.00000E+00
1.02201E-08
1.52201E-08
1.99999E-08
5.00000E-09
9.99998E-09

1.50000E-08
0.00000E+00
1.00000E-08
4.99996E-09
2.85000E-07
1.00000E-08
1.50000E-08
1.02201E-08
5.22015E-09
5.00000E-09
0.00000E+00
2.52201E-08
0.00000E+00
5.00000E-09
5.22015E-09
0.00000E+00
0.00000E+00
0.00000E+00
1.52201E-08
1.00000E-08
5.22015E-09
1.00000E-08
0.00000E+00
0.00000E+00
1.00000E-08
4.99996E-09
5.00000E-09
1.02201E-08
0.00000E+00
1.02201E-08
0.00000E+00
0.00000E+00
0.00000E+00
1.50000E-08
1.02201E-08
1.00000E-08
1.02201E-08
5.00000E-09
1.00000E-08
9.99996E-09
2.50000E-08
2.50000E-08
1.52201E-08
0.00000E+00
0.00000E+00
1.50000E-08
1.50000E-08
0.00000E+00
0.00000E+00
0.00000E+00
0.00000E+00
5.00000E-09
0.00000E+00
1.00000E-08

1.00000E-08
0.00000E+00
1.00000E-08
0.00000E+00
5.00000E-09
0.00000E+00
0.00000E+00
1.50000E-08
1.00000E-08
1.50000E-08
5.00000E-09
1.52201E-08
5.00000E-09
1.02201E-08
9.99996E-09
0.00000E+00
5.00000E-09
1.00000E-08
1.00000E-08
0.00000E+00
0.00000E+00
5.00000E-09
5.00000E-09
0.00000E+00
0.00000E+00
1.02201E-08
5.00000E-09
5.00000E-09
0.00000E+00
5.00000E-09
0.00000E+00
5.00000E-09
5.00000E-09
0.00000E+00
1.00000E-08
0.00000E+00
0.00000E+00
0.00000E+00
5.00000E-09
0.00000E+00
0.00000E+00
0.00000E+00
0.00000E+00
5.00000E-09
5.00000E-09
2.50000E-08
1.00000E-08
5.00000E-09
0.00000E+00
0.00000E+00
0.00000E+00
5.00000E-09
0.00000E+00
4.99996E-09

```
     5.22015E-09
     0.00000E+00
     1.00000E-08
     5.00000E-09
     5.00000E-09
     0.00000E+00
     0.00000E+00
     1.50000E-08
     0.00000E+00
     5.00000E-09
     1.00000E-08
     0.00000E+00
     0.00000E+00
     5.00000E-09
cut:p j 0.001                       $ photon cut-off energy 1.0 keV
f8:p  1                             $ Pulse height tally defined for cell
1 (NaI detector)
e8    0.0 1.0e-5 0.01 1198i 12.0    $ Energy binning
ft8   geb  0.016410  0.076003  0.0  $ Tally treatment with GEB
nps   2.0e+8                        $ Number of histories
```

5. Pure SiO2 / 10 cm seawater layer

```
1   3  -7.9   -1  2                            IMP:N=1 IMP:P=1 $ neutron
generator
2   0         -2                               IMP:N=1 IMP:P=1 $ inner
generator
3   7  -3.67  -3                               IMP:N=1 IMP:P=0 $ NaI
4   8  -2.7    3 -4                            IMP:N=1 IMP:P=1 $ Al ring
6   8  -2.7   -6                               IMP:N=1 IMP:P=1 $ Al slab
7   5  -7.9  -20                               IMP:N=1 IMP:P=1 $ shadow
bar (Iron)
8  10 -11.35 -21                               IMP:N=1 IMP:P=1 $ shadow
bar (lead)
11 12  -1.43 -11:-12:-13:-14:-15:-16:-17:-18  IMP:N=1 IMP:P=1 $ Vessel
12 12  -1.43 -10                               imp:n=1 imp:p=1 $ Vessel
top
40  4  -1.225e-3  -101 #1 #2 #3 #4 #6 #7 #8 #11 #12 &
                                               IMP:N=1 IMP:P=1 $ Vessel
inside (air)
41  1  -1.025 -100 101                         IMP:N=1 IMP:P=1 $ Seawater
42  2  -2.196   -30                            imp:n=1 imp:p=1 $ Seabed
99  0       100 #42                          IMP:N=0 IMP:P=0  $ external
void

C -----------------------------------------------------------------------
-
C                    (((((      surfaces      )))))
C -----------------------------------------------------------------------
-
1    RCC   0  -5     0   0  100     0      3.81           $ neutron
generator
2    RCC   0  -4.6   0   0   99.2   0      3.41           $ neutron
generator
3    RCC -40   0   -19   0    0     7.62   3.81           $ 3"x3" NaI
cristal
4    RCC -40   0   -19   0    0     7.62   4.3            $ Al ring
C
6    RPP  -46     0  -13    13    -19.5  -19              $ Al slab
C
C    Vessel : polyester/kevlar=6/4 - half down part : 30kg
C
10   RPP -100   5  -16  96   29.5  30.5                   $ Top
11   RPP -100 -99  -16  96  -18.5  29.5                   $ left side
with Top
12   RPP    4   5  -16  96  -18.5  29.5                   $ right side
with Top
13   RPP -100 -46  -16  96  -19.5 -18.5                   $ left side of
the floor
14   RPP    0   5  -16  96  -19.5 -18.5                   $ right side of
the floor
15   RPP  -46   0  -16 -13  -19.5 -18.5                   $ front side of
the floor
16   RPP  -46   0   13  96  -19.5 -18.5                   $ back side of
the floor
```

```
17   RPP -100   5   95  96  -18.5  29.5                  $ front side
with Top
18   RPP -100   5  -16 -15  -18.5  29.5                  $ back side
with Top
C
20   1 TRC  -5     0  0  -19.99  0   0  1.75  3.92        $ Shadow Bar Fe
21   1 TRC -24.99  0  0  -10     0   0  3.92  5           $ Shadow Bar Pb
C
30   RPP -210  150  -150 200  -155  -29.5                $ seabed (SiO2
+ Gd)
100  RPP -210  150  -150 200  -29.5  150                    $ seawater
101  RPP -100.0001 5.0001 -16.0001 96.0001 -19.5001 30.5001 $ Vessel
inside

C -----------------------------------------------------------------------
-
C                  (((((      Transformation      )))))
C
C -----------------------------------------------------------------------
-
C    X' Y' Z' xx' yx' zx'  xy' yy' zy'  xz' yz' zz'
C
C  26 degrees rotation
C
*tr1 -1.8 0 1 26 90 64 90 0 90 116 90 26
C
C -----------------------------------------------------------------------
-
C
C -----------------------------------------------------------------------
-
C                 (((((      Source definition      )))))
C -----------------------------------------------------------------------
-
C
MODE N P
c
C Neutron source definition, E=14 MeV, pulsed neutron source
C
sdef  erg=14  pos=0. 0.1 0.1  vec=-0.77715 0 -0.62932 par=1 dir=d1 tme=d2
si1  -1.0  0.993  1.0
sp1   0.0  0.0    1.0
si2 h 0 1000
sp2 d 0 1
lca 8j 1 1
C -----------------------------------------------------------------------
-
C                       (((((      Tally      )))))
C -----------------------------------------------------------------------
-
C
f11:p (3.1 3.2 3.3)  $ Surface current tally, scoring incident photon
energies
e11 0.01 1198i 12.0  $ Energy binning
```

```
t11: 1000 10000 15000000 $ Time binning
c
C ------------------------------------------------------------------------
-
C                      (((((      Materials     )))))
C ------------------------------------------------------------------------
-
C
c =========================== Sea water
==================================
C
M1         8016.80c -0.858142 &
           8017.80c -0.000347 &
           1001.80c -0.108186 &
           1002.80c -0.000025 &
          17035.80c -0.014503 &
          17037.80c -0.004899 &
          11023.80c -0.010801 &
          12024.80c -0.001007 &
          12025.80c -0.000133 &
          12026.80c -0.000152 &
          16032.80c -0.000861 &
          16033.80c -0.000007 &
          16034.80c -0.000041 &
          19039.80c -0.000372 &
          19041.80c -0.000028 &
          20040.80c -0.000387 &
          20042.80c -0.000003 &
          20043.80c -0.000001 &
          20044.80c -0.000009 &
          20048.80c -0.000001 &
          35079.80c -0.000034 &
          35081.80c -0.000033 &
           6000.80c -0.000028
C
c
c ============================ SiO2 ========================
=============
C
m2   14028.80c   -0.4310058422103860 &
     14029.80c   -0.0219710136484690 &
     14030.80c   -0.0144915196404794 &
      8016.80c   -0.5312535486018640 &
      8017.80c   -0.0012780758988016
C
c
c ============================ Inox ========================
=============
C
M3        26054.80c -3.94109804245289E-02 &
          26056.80c -6.40977517311647E-01 &
          26057.80c -1.50755011378944E-02 &
          26058.80c -2.02600112593001E-03 &
          24050.80c -7.59652441069589E-03 &
```

```
          24052.80c -1.52167870331914E-01 &
          24053.80c -1.75848008017023E-02 &
          24054.80c -4.45080445568747E-03 &
          28058.80c -6.82087476171617E-02 &
          28060.80c -2.71743778762732E-02 &
          28061.80c -1.20121432630459E-03 &
          28062.80c -3.8875236702706E-03 &
          28064.80c -1.02813650998988E-03 &
          25055.80c -1.38E-02 &
          14028.80c -4.59367138104534E-03 &
          14029.80c -2.40907566547312E-04 &
          14030.80c -1.65421052407344E-04 &
          15031.80c -2.6E-04 &
           6000.80c -1.5E-04
c
C =========================== air WEIGHT CHEMICAL FORMULA
================
C
M4         7014.80c 0.8  &
           8016.80c 0.2
c
C =========================== Iron
=======================================
C
M5        26054.80c 0.058   &
          26056.80c 0.9172  &
          26057.80c 0.022   &
          26058.80c 0.0028
c
C =========================== NaI CHEMICAL FORMULA
=======================
C
M7        11023.80c  1 &
          53127.80c  1
c
C =========================== Aluminium
==================================
C
M8        13027.80c  1
c
C =========================== Lead
=======================================
C
M10      82204.80c -0.013781  82206.80c -0.239555  82207.80c -0.220743
         82208.80c -0.525921
C
C =========================== polyester/kevlar
===========================
C
C --> polyester/kevlar = 6/4
C
M12       1001.80c -0.056987   1002.80c -0.000013   6000.80c -0.635300
          7014.80c -0.051098   7015.80c -0.000202   8016.80c -0.256296
          8017.80c -0.000104
```

```
C
c -----------------------------------------------------------------------
-
nps 2.0e+8     $ Number of histories to be simulated
cut:n j 0      $ Neutron cut-off energy = 0.0 eV
cut:p j 0.001  $ Photon cut-off energy = 1.0 keV
```

6. SiO2 + 150 ppm Gd / 10 cm seawater layer

```
1   3  -7.9   -1  2                              IMP:N=1 IMP:P=1 $ neutron
generator
2   0         -2                                 IMP:N=1 IMP:P=1 $ inner
generator
3   7  -3.67  -3                                 IMP:N=1 IMP:P=0 $ NaI
4   8  -2.7    3 -4                              IMP:N=1 IMP:P=1 $ Al ring
6   8  -2.7   -6                                 IMP:N=1 IMP:P=1 $ Al slab
7   5  -7.9  -20                                 IMP:N=1 IMP:P=1 $ shadow
bar (Iron)
8  10 -11.35 -21                                 IMP:N=1 IMP:P=1 $ shadow
bar (lead)
11 12  -1.43 -11:-12:-13:-14:-15:-16:-17:-18  IMP:N=1 IMP:P=1 $ Vessel
12 12  -1.43 -10                                 imp:n=1 imp:p=1 $ Vessel
top
40  4  -1.225e-3  -101 #1 #2 #3 #4 #6 #7 #8 #11 #12 &
                                                 IMP:N=1 IMP:P=1 $ Vessel
inside (air)
41  1  -1.025 -100 101                           IMP:N=1 IMP:P=1 $ Seawater
42  2  -2.196238 -30                              imp:n=1 imp:p=1 $ Seabed
99  0       100 #42                            IMP:N=0 IMP:P=0  $ external
void

C --------------------------------------------------------------------------
-
C                     (((((      surfaces      )))))
C --------------------------------------------------------------------------
-
1    RCC   0  -5     0   0  100     0      3.81          $ neutron
generator
2    RCC   0  -4.6   0   0   99.2   0      3.41          $ neutron
generator
3    RCC -40   0   -19   0    0     7.62   3.81          $ 3"x3" NaI
cristal
4    RCC -40   0   -19   0    0     7.62   4.3           $ Al ring
C
6    RPP  -46     0  -13    13    -19.5  -19             $ Al slab
C
C    Vessel : polyester/kevlar=6/4 - half down part : 30kg
C
10   RPP -100   5  -16  96   29.5  30.5                  $ Top
11   RPP -100 -99  -16  96  -18.5  29.5                  $ left side
with Top
12   RPP    4   5  -16  96  -18.5  29.5                  $ right side
with Top
13   RPP -100 -46  -16  96  -19.5 -18.5                  $ left side of
the floor
14   RPP    0   5  -16  96  -19.5 -18.5                  $ right side of
the floor
15   RPP  -46   0  -16 -13  -19.5 -18.5                  $ front side of
the floor
16   RPP  -46   0   13  96  -19.5 -18.5                  $ back side of
the floor
```

```
17   RPP -100   5   95  96  -18.5  29.5                  $ front side
with Top
18   RPP -100   5  -16 -15  -18.5  29.5                  $ back side
with Top
C
20   1 TRC  -5      0  0  -19.99  0   0  1.75  3.92       $ Shadow Bar Fe
21   1 TRC -24.99  0  0  -10     0   0  3.92  5          $ Shadow Bar Pb
C
30   RPP -210  150  -150 200  -155  -29.5                $ seabed (SiO2
+ Gd)
100  RPP -210  150  -150 200  -29.5  150                    $ seawater
101  RPP -100.0001 5.0001 -16.0001 96.0001 -19.5001 30.5001 $ Vessel
inside

C ----------------------------------------------------------------------
-
C                  (((((      Transformation      )))))
C
C ----------------------------------------------------------------------
-
C    X' Y' Z' xx' yx' zx'  xy' yy' zy'  xz' yz' zz'
C
C  26 degrees rotation
C
*tr1 -1.8 0 1 26 90 64 90 0 90 116 90 26
C
C ----------------------------------------------------------------------
-
C
C ----------------------------------------------------------------------
-
C                 (((((      Source definition      )))))
C ----------------------------------------------------------------------
-
C
MODE N P
C
C Neutron source definition, E=14 MeV, pulsed neutron source
C
sdef  erg=14  pos=0. 0.1 0.1  vec=-0.77715 0 -0.62932 par=n dir=d1 tme=d2
si1  -1.0  0.993  1.0
sp1   0.0  0.0    1.0
si2 h 0 1000
sp2 d 0 1
lca 8j 1 1
C ----------------------------------------------------------------------
-
C                        (((((      Tally      )))))
C ----------------------------------------------------------------------
-
C
f11:p (3.1 3.2 3.3)  $ Surface current tally, scoring incident photon
energies
e11 0.01 1198i 12.0  $ Energy binning
```

```
t11: 1000 10000 15000000 $ Time binning
c
C ------------------------------------------------------------------
-
C                     (((((      Materials     )))))
C ------------------------------------------------------------------
-
C
c =========================== Sea water
=================================
C
M1         8016.80c -0.858142 &
           8017.80c -0.000347 &
           1001.80c -0.108186 &
           1002.80c -0.000025 &
          17035.80c -0.014503 &
          17037.80c -0.004899 &
          11023.80c -0.010801 &
          12024.80c -0.001007 &
          12025.80c -0.000133 &
          12026.80c -0.000152 &
          16032.80c -0.000861 &
          16033.80c -0.000007 &
          16034.80c -0.000041 &
          19039.80c -0.000372 &
          19041.80c -0.000028 &
          20040.80c -0.000387 &
          20042.80c -0.000003 &
          20043.80c -0.000001 &
          20044.80c -0.000009 &
          20048.80c -0.000001 &
          35079.80c -0.000034 &
          35081.80c -0.000033 &
           6000.80c -0.000028
C
c
c ============================ SiO2 + 150 ppm Gd=======================
=============
C
m2   14028.80c   -0.430941191334055&
     14029.80c   -0.021967717996421&
     14030.80c   -0.014489345912533&
      8016.80c   -0.531173860569574&
      8017.80c   -0.001277884187417&
     64152.80c   -3.000000E-07&
     64154.80c   -3.270000E-06&
     64155.80c   -2.220000E-05&
     64156.80c   -3.070500E-05&
     64157.80c   -2.347500E-05&
     64158.80c   -3.726000E-05&
     64160.80c   -3.279000E-05
C
c
```

```
c ============================ Inox ========================
=============
C
M3       26054.80c -3.94109804245289E-02 &
         26056.80c -6.40977517311647E-01 &
         26057.80c -1.50755011378944E-02 &
         26058.80c -2.02600112593001E-03 &
         24050.80c -7.59652441069589E-03 &
         24052.80c -1.52167870331914E-01 &
         24053.80c -1.75848008017023E-02 &
         24054.80c -4.45080445568747E-03 &
         28058.80c -6.82087476171617E-02 &
         28060.80c -2.71743778762732E-02 &
         28061.80c -1.20121432630459E-03 &
         28062.80c -3.8875236702706E-03 &
         28064.80c -1.02813650998988E-03 &
         25055.80c -1.38E-02 &
         14028.80c -4.59367138104534E-03 &
         14029.80c -2.40907566547312E-04 &
         14030.80c -1.65421052407344E-04 &
         15031.80c -2.6E-04 &
          6000.80c -1.5E-04
c
C ============================ air WEIGHT CHEMICAL FORMULA
================
C
M4        7014.80c 0.8  &
          8016.80c 0.2
c
C ============================ Iron
=======================================
C
M5       26054.80c 0.058   &
         26056.80c 0.9172  &
         26057.80c 0.022   &
         26058.80c 0.0028
c
C ============================ NaI CHEMICAL FORMULA
=======================
C
M7       11023.80c  1 &
         53127.80c  1
c
C ============================ Aluminium
==================================
C
M8       13027.80c  1
c
C ============================ Lead
=======================================
C
M10     82204.80c -0.013781  82206.80c -0.239555  82207.80c -0.220743
        82208.80c -0.525921
C
```

```
C ============================ polyester/kevlar
===========================
C
C --> polyester/kevlar = 6/4
C
M12      1001.80c -0.056987   1002.80c -0.000013   6000.80c -0.635300
         7014.80c -0.051098   7015.80c -0.000202   8016.80c -0.256296
         8017.80c -0.000104
C
c --------------------------------------------------------------------------
-
nps 2.0e+8     $ Number of histories to be simulated
cut:n j 0      $ Neutron cut-off energy = 0.0 eV
cut:p j 0.001  $ Photon cut-off energy = 1.0 keV
```

7. Pure SiO2 / GEB / 10 cm seawater layer

```
c     SiO2
c     Pulse 'OFF'
c     Using re-normalized f1 data
c     Particle weight determined from the total of f1 data
c
c
c   Problem Cells
c
1  1 -3.67       1  -2  -3    imp:p=1     $ crystal NaI
2  2 -1.22e-3  -4  #1         imp:p=1     $ Inner world (air)
3  0            4             imp:p=0     $ Outer world (void)

c
c   Problem Surfaces
c
1  pz  0.0
2  pz  7.62
3  cz  3.81
4  so  100.0

c
c   Problem data cards
c
mode p                   $ photon only problem
c
c  NaI
c
m1   11023.80c   1
     53127.80c   1
c
c  Air
c
m2    7014.80c   0.8
      8016.80c   0.2
c
c   Source definition -- energy is now a prob. dist.
c
sdef erg=d2  rad=d1  pos=0.0 0.0 -3.0  vec=0 0 1  dir=1  ext=0
     axs=0 0 1 wgt=0.0638207
si1   0  3.81    $ radial sampling range: 0 to 3.81
sp1  -21 1       $ uniform sampling over the disk area
si2 h 0.000e+00  $ source energy binning / histogram
      1.000e-02
      2.000e-02
      3.000e-02
      4.000e-02
      5.000e-02
      6.000e-02
      7.000e-02
      8.000e-02
      9.000e-02
      1.000e-01
      1.100e-01
```

```
1.200e-01
1.300e-01
1.400e-01
1.500e-01
1.600e-01
1.700e-01
1.800e-01
1.900e-01
2.000e-01
2.100e-01
2.200e-01
2.300e-01
2.400e-01
2.500e-01
2.600e-01
2.700e-01
2.800e-01
2.900e-01
3.000e-01
3.100e-01
3.200e-01
3.300e-01
3.400e-01
3.500e-01
3.600e-01
3.700e-01
3.800e-01
3.900e-01
4.000e-01
4.100e-01
4.200e-01
4.300e-01
4.400e-01
4.500e-01
4.600e-01
4.700e-01
4.800e-01
4.900e-01
5.000e-01
5.100e-01
5.200e-01
5.300e-01
5.400e-01
5.500e-01
5.600e-01
5.700e-01
5.800e-01
5.900e-01
6.000e-01
6.100e-01
6.200e-01
6.300e-01
6.400e-01
6.500e-01
```

6.600e-01
6.700e-01
6.800e-01
6.900e-01
7.000e-01
7.100e-01
7.200e-01
7.300e-01
7.400e-01
7.500e-01
7.600e-01
7.700e-01
7.800e-01
7.900e-01
8.000e-01
8.100e-01
8.200e-01
8.300e-01
8.400e-01
8.500e-01
8.600e-01
8.700e-01
8.800e-01
8.900e-01
9.000e-01
9.100e-01
9.200e-01
9.300e-01
9.400e-01
9.500e-01
9.600e-01
9.700e-01
9.800e-01
9.900e-01
1.000e+00
1.010e+00
1.020e+00
1.030e+00
1.040e+00
1.050e+00
1.060e+00
1.070e+00
1.080e+00
1.090e+00
1.100e+00
1.110e+00
1.120e+00
1.130e+00
1.140e+00
1.150e+00
1.160e+00
1.170e+00
1.180e+00
1.190e+00

1.200e+00
1.210e+00
1.220e+00
1.230e+00
1.240e+00
1.250e+00
1.260e+00
1.270e+00
1.280e+00
1.290e+00
1.300e+00
1.310e+00
1.320e+00
1.330e+00
1.340e+00
1.350e+00
1.360e+00
1.370e+00
1.380e+00
1.390e+00
1.400e+00
1.410e+00
1.420e+00
1.430e+00
1.440e+00
1.450e+00
1.460e+00
1.470e+00
1.480e+00
1.490e+00
1.500e+00
1.510e+00
1.520e+00
1.530e+00
1.540e+00
1.550e+00
1.560e+00
1.570e+00
1.580e+00
1.590e+00
1.600e+00
1.610e+00
1.620e+00
1.630e+00
1.640e+00
1.650e+00
1.660e+00
1.670e+00
1.680e+00
1.690e+00
1.700e+00
1.710e+00
1.720e+00
1.730e+00

1.740e+00
1.750e+00
1.760e+00
1.770e+00
1.780e+00
1.790e+00
1.800e+00
1.810e+00
1.820e+00
1.830e+00
1.840e+00
1.850e+00
1.860e+00
1.870e+00
1.880e+00
1.890e+00
1.900e+00
1.910e+00
1.920e+00
1.930e+00
1.940e+00
1.950e+00
1.960e+00
1.970e+00
1.980e+00
1.990e+00
2.000e+00
2.010e+00
2.020e+00
2.030e+00
2.040e+00
2.050e+00
2.060e+00
2.070e+00
2.080e+00
2.090e+00
2.100e+00
2.110e+00
2.120e+00
2.130e+00
2.140e+00
2.150e+00
2.160e+00
2.170e+00
2.180e+00
2.190e+00
2.200e+00
2.210e+00
2.220e+00
2.230e+00
2.240e+00
2.250e+00
2.260e+00
2.270e+00

2.280e+00
2.290e+00
2.300e+00
2.310e+00
2.320e+00
2.330e+00
2.340e+00
2.350e+00
2.360e+00
2.370e+00
2.380e+00
2.390e+00
2.400e+00
2.410e+00
2.420e+00
2.430e+00
2.440e+00
2.450e+00
2.460e+00
2.470e+00
2.480e+00
2.490e+00
2.500e+00
2.510e+00
2.520e+00
2.530e+00
2.540e+00
2.550e+00
2.560e+00
2.570e+00
2.580e+00
2.590e+00
2.600e+00
2.610e+00
2.620e+00
2.630e+00
2.640e+00
2.650e+00
2.660e+00
2.670e+00
2.680e+00
2.690e+00
2.700e+00
2.710e+00
2.720e+00
2.730e+00
2.740e+00
2.750e+00
2.760e+00
2.770e+00
2.780e+00
2.790e+00
2.800e+00
2.810e+00

2.820e+00
2.830e+00
2.840e+00
2.850e+00
2.860e+00
2.870e+00
2.880e+00
2.890e+00
2.900e+00
2.910e+00
2.920e+00
2.930e+00
2.940e+00
2.950e+00
2.960e+00
2.970e+00
2.980e+00
2.990e+00
3.000e+00
3.010e+00
3.020e+00
3.030e+00
3.040e+00
3.050e+00
3.060e+00
3.070e+00
3.080e+00
3.090e+00
3.100e+00
3.110e+00
3.120e+00
3.130e+00
3.140e+00
3.150e+00
3.160e+00
3.170e+00
3.180e+00
3.190e+00
3.200e+00
3.210e+00
3.220e+00
3.230e+00
3.240e+00
3.250e+00
3.260e+00
3.270e+00
3.280e+00
3.290e+00
3.300e+00
3.310e+00
3.320e+00
3.330e+00
3.340e+00
3.350e+00

3.360e+00
3.370e+00
3.380e+00
3.390e+00
3.400e+00
3.410e+00
3.420e+00
3.430e+00
3.440e+00
3.450e+00
3.460e+00
3.470e+00
3.480e+00
3.490e+00
3.500e+00
3.510e+00
3.520e+00
3.530e+00
3.540e+00
3.550e+00
3.560e+00
3.570e+00
3.580e+00
3.590e+00
3.600e+00
3.610e+00
3.620e+00
3.630e+00
3.640e+00
3.650e+00
3.660e+00
3.670e+00
3.680e+00
3.690e+00
3.700e+00
3.710e+00
3.720e+00
3.730e+00
3.740e+00
3.750e+00
3.760e+00
3.770e+00
3.780e+00
3.790e+00
3.800e+00
3.810e+00
3.820e+00
3.830e+00
3.840e+00
3.850e+00
3.860e+00
3.870e+00
3.880e+00
3.890e+00

3.900e+00
3.910e+00
3.920e+00
3.930e+00
3.940e+00
3.950e+00
3.960e+00
3.970e+00
3.980e+00
3.990e+00
4.000e+00
4.010e+00
4.020e+00
4.030e+00
4.040e+00
4.050e+00
4.060e+00
4.070e+00
4.080e+00
4.090e+00
4.100e+00
4.110e+00
4.120e+00
4.130e+00
4.140e+00
4.150e+00
4.160e+00
4.170e+00
4.180e+00
4.190e+00
4.200e+00
4.210e+00
4.220e+00
4.230e+00
4.240e+00
4.250e+00
4.260e+00
4.270e+00
4.280e+00
4.290e+00
4.300e+00
4.310e+00
4.320e+00
4.330e+00
4.340e+00
4.350e+00
4.360e+00
4.370e+00
4.380e+00
4.390e+00
4.400e+00
4.410e+00
4.420e+00
4.430e+00

4.440e+00
4.450e+00
4.460e+00
4.470e+00
4.480e+00
4.490e+00
4.500e+00
4.510e+00
4.520e+00
4.530e+00
4.540e+00
4.550e+00
4.560e+00
4.570e+00
4.580e+00
4.590e+00
4.600e+00
4.610e+00
4.620e+00
4.630e+00
4.640e+00
4.650e+00
4.660e+00
4.670e+00
4.680e+00
4.690e+00
4.700e+00
4.710e+00
4.720e+00
4.730e+00
4.740e+00
4.750e+00
4.760e+00
4.770e+00
4.780e+00
4.790e+00
4.800e+00
4.810e+00
4.820e+00
4.830e+00
4.840e+00
4.850e+00
4.860e+00
4.870e+00
4.880e+00
4.890e+00
4.900e+00
4.910e+00
4.920e+00
4.930e+00
4.940e+00
4.950e+00
4.960e+00
4.970e+00

4.980e+00
4.990e+00
5.000e+00
5.010e+00
5.020e+00
5.030e+00
5.040e+00
5.050e+00
5.060e+00
5.070e+00
5.080e+00
5.090e+00
5.100e+00
5.110e+00
5.120e+00
5.130e+00
5.140e+00
5.150e+00
5.160e+00
5.170e+00
5.180e+00
5.190e+00
5.200e+00
5.210e+00
5.220e+00
5.230e+00
5.240e+00
5.250e+00
5.260e+00
5.270e+00
5.280e+00
5.290e+00
5.300e+00
5.310e+00
5.320e+00
5.330e+00
5.340e+00
5.350e+00
5.360e+00
5.370e+00
5.380e+00
5.390e+00
5.400e+00
5.410e+00
5.420e+00
5.430e+00
5.440e+00
5.450e+00
5.460e+00
5.470e+00
5.480e+00
5.490e+00
5.500e+00
5.510e+00

5.520e+00
5.530e+00
5.540e+00
5.550e+00
5.560e+00
5.570e+00
5.580e+00
5.590e+00
5.600e+00
5.610e+00
5.620e+00
5.630e+00
5.640e+00
5.650e+00
5.660e+00
5.670e+00
5.680e+00
5.690e+00
5.700e+00
5.710e+00
5.720e+00
5.730e+00
5.740e+00
5.750e+00
5.760e+00
5.770e+00
5.780e+00
5.790e+00
5.800e+00
5.810e+00
5.820e+00
5.830e+00
5.840e+00
5.850e+00
5.860e+00
5.870e+00
5.880e+00
5.890e+00
5.900e+00
5.910e+00
5.920e+00
5.930e+00
5.940e+00
5.950e+00
5.960e+00
5.970e+00
5.980e+00
5.990e+00
6.000e+00
6.010e+00
6.020e+00
6.030e+00
6.040e+00
6.050e+00

6.060e+00
6.070e+00
6.080e+00
6.090e+00
6.100e+00
6.110e+00
6.120e+00
6.130e+00
6.140e+00
6.150e+00
6.160e+00
6.170e+00
6.180e+00
6.190e+00
6.200e+00
6.210e+00
6.220e+00
6.230e+00
6.240e+00
6.250e+00
6.260e+00
6.270e+00
6.280e+00
6.290e+00
6.300e+00
6.310e+00
6.320e+00
6.330e+00
6.340e+00
6.350e+00
6.360e+00
6.370e+00
6.380e+00
6.390e+00
6.400e+00
6.410e+00
6.420e+00
6.430e+00
6.440e+00
6.450e+00
6.460e+00
6.470e+00
6.480e+00
6.490e+00
6.500e+00
6.510e+00
6.520e+00
6.530e+00
6.540e+00
6.550e+00
6.560e+00
6.570e+00
6.580e+00
6.590e+00

6.600e+00
6.610e+00
6.620e+00
6.630e+00
6.640e+00
6.650e+00
6.660e+00
6.670e+00
6.680e+00
6.690e+00
6.700e+00
6.710e+00
6.720e+00
6.730e+00
6.740e+00
6.750e+00
6.760e+00
6.770e+00
6.780e+00
6.790e+00
6.800e+00
6.810e+00
6.820e+00
6.830e+00
6.840e+00
6.850e+00
6.860e+00
6.870e+00
6.880e+00
6.890e+00
6.900e+00
6.910e+00
6.920e+00
6.930e+00
6.940e+00
6.950e+00
6.960e+00
6.970e+00
6.980e+00
6.990e+00
7.000e+00
7.010e+00
7.020e+00
7.030e+00
7.040e+00
7.050e+00
7.060e+00
7.070e+00
7.080e+00
7.090e+00
7.100e+00
7.110e+00
7.120e+00
7.130e+00

7.140e+00
7.150e+00
7.160e+00
7.170e+00
7.180e+00
7.190e+00
7.200e+00
7.210e+00
7.220e+00
7.230e+00
7.240e+00
7.250e+00
7.260e+00
7.270e+00
7.280e+00
7.290e+00
7.300e+00
7.310e+00
7.320e+00
7.330e+00
7.340e+00
7.350e+00
7.360e+00
7.370e+00
7.380e+00
7.390e+00
7.400e+00
7.410e+00
7.420e+00
7.430e+00
7.440e+00
7.450e+00
7.460e+00
7.470e+00
7.480e+00
7.490e+00
7.500e+00
7.510e+00
7.520e+00
7.530e+00
7.540e+00
7.550e+00
7.560e+00
7.570e+00
7.580e+00
7.590e+00
7.600e+00
7.610e+00
7.620e+00
7.630e+00
7.640e+00
7.650e+00
7.660e+00
7.670e+00

7.680e+00
7.690e+00
7.700e+00
7.710e+00
7.720e+00
7.730e+00
7.740e+00
7.750e+00
7.760e+00
7.770e+00
7.780e+00
7.790e+00
7.800e+00
7.810e+00
7.820e+00
7.830e+00
7.840e+00
7.850e+00
7.860e+00
7.870e+00
7.880e+00
7.890e+00
7.900e+00
7.910e+00
7.920e+00
7.930e+00
7.940e+00
7.950e+00
7.960e+00
7.970e+00
7.980e+00
7.990e+00
8.000e+00
8.010e+00
8.020e+00
8.030e+00
8.040e+00
8.050e+00
8.060e+00
8.070e+00
8.080e+00
8.090e+00
8.100e+00
8.110e+00
8.120e+00
8.130e+00
8.140e+00
8.150e+00
8.160e+00
8.170e+00
8.180e+00
8.190e+00
8.200e+00
8.210e+00

8.220e+00
8.230e+00
8.240e+00
8.250e+00
8.260e+00
8.270e+00
8.280e+00
8.290e+00
8.300e+00
8.310e+00
8.320e+00
8.330e+00
8.340e+00
8.350e+00
8.360e+00
8.370e+00
8.380e+00
8.390e+00
8.400e+00
8.410e+00
8.420e+00
8.430e+00
8.440e+00
8.450e+00
8.460e+00
8.470e+00
8.480e+00
8.490e+00
8.500e+00
8.510e+00
8.520e+00
8.530e+00
8.540e+00
8.550e+00
8.560e+00
8.570e+00
8.580e+00
8.590e+00
8.600e+00
8.610e+00
8.620e+00
8.630e+00
8.640e+00
8.650e+00
8.660e+00
8.670e+00
8.680e+00
8.690e+00
8.700e+00
8.710e+00
8.720e+00
8.730e+00
8.740e+00
8.750e+00

8.760e+00
8.770e+00
8.780e+00
8.790e+00
8.800e+00
8.810e+00
8.820e+00
8.830e+00
8.840e+00
8.850e+00
8.860e+00
8.870e+00
8.880e+00
8.890e+00
8.900e+00
8.910e+00
8.920e+00
8.930e+00
8.940e+00
8.950e+00
8.960e+00
8.970e+00
8.980e+00
8.990e+00
9.000e+00
9.010e+00
9.020e+00
9.030e+00
9.040e+00
9.050e+00
9.060e+00
9.070e+00
9.080e+00
9.090e+00
9.100e+00
9.110e+00
9.120e+00
9.130e+00
9.140e+00
9.150e+00
9.160e+00
9.170e+00
9.180e+00
9.190e+00
9.200e+00
9.210e+00
9.220e+00
9.230e+00
9.240e+00
9.250e+00
9.260e+00
9.270e+00
9.280e+00
9.290e+00

9.300e+00
9.310e+00
9.320e+00
9.330e+00
9.340e+00
9.350e+00
9.360e+00
9.370e+00
9.380e+00
9.390e+00
9.400e+00
9.410e+00
9.420e+00
9.430e+00
9.440e+00
9.450e+00
9.460e+00
9.470e+00
9.480e+00
9.490e+00
9.500e+00
9.510e+00
9.520e+00
9.530e+00
9.540e+00
9.550e+00
9.560e+00
9.570e+00
9.580e+00
9.590e+00
9.600e+00
9.610e+00
9.620e+00
9.630e+00
9.640e+00
9.650e+00
9.660e+00
9.670e+00
9.680e+00
9.690e+00
9.700e+00
9.710e+00
9.720e+00
9.730e+00
9.740e+00
9.750e+00
9.760e+00
9.770e+00
9.780e+00
9.790e+00
9.800e+00
9.810e+00
9.820e+00
9.830e+00

9.840e+00
9.850e+00
9.860e+00
9.870e+00
9.880e+00
9.890e+00
9.900e+00
9.910e+00
9.920e+00
9.930e+00
9.940e+00
9.950e+00
9.960e+00
9.970e+00
9.980e+00
9.990e+00
1.000e+01
1.001e+01
1.002e+01
1.003e+01
1.004e+01
1.005e+01
1.006e+01
1.007e+01
1.008e+01
1.009e+01
1.010e+01
1.011e+01
1.012e+01
1.013e+01
1.014e+01
1.015e+01
1.016e+01
1.017e+01
1.018e+01
1.019e+01
1.020e+01
1.021e+01
1.022e+01
1.023e+01
1.024e+01
1.025e+01
1.026e+01
1.027e+01
1.028e+01
1.029e+01
1.030e+01
1.031e+01
1.032e+01
1.033e+01
1.034e+01
1.035e+01
1.036e+01
1.037e+01

1.038e+01
1.039e+01
1.040e+01
1.041e+01
1.042e+01
1.043e+01
1.044e+01
1.045e+01
1.046e+01
1.047e+01
1.048e+01
1.049e+01
1.050e+01
1.051e+01
1.052e+01
1.053e+01
1.054e+01
1.055e+01
1.056e+01
1.057e+01
1.058e+01
1.059e+01
1.060e+01
1.061e+01
1.062e+01
1.063e+01
1.064e+01
1.065e+01
1.066e+01
1.067e+01
1.068e+01
1.069e+01
1.070e+01
1.071e+01
1.072e+01
1.073e+01
1.074e+01
1.075e+01
1.076e+01
1.077e+01
1.078e+01
1.079e+01
1.080e+01
1.081e+01
1.082e+01
1.083e+01
1.084e+01
1.085e+01
1.086e+01
1.087e+01
1.088e+01
1.089e+01
1.090e+01
1.091e+01

1.092e+01
1.093e+01
1.094e+01
1.095e+01
1.096e+01
1.097e+01
1.098e+01
1.099e+01
1.100e+01
1.101e+01
1.102e+01
1.103e+01
1.104e+01
1.105e+01
1.106e+01
1.107e+01
1.108e+01
1.109e+01
1.110e+01
1.111e+01
1.112e+01
1.113e+01
1.114e+01
1.115e+01
1.116e+01
1.117e+01
1.118e+01
1.119e+01
1.120e+01
1.121e+01
1.122e+01
1.123e+01
1.124e+01
1.125e+01
1.126e+01
1.127e+01
1.128e+01
1.129e+01
1.130e+01
1.131e+01
1.132e+01
1.133e+01
1.134e+01
1.135e+01
1.136e+01
1.137e+01
1.138e+01
1.139e+01
1.140e+01
1.141e+01
1.142e+01
1.143e+01
1.144e+01
1.145e+01

1.146e+01
1.147e+01
1.148e+01
1.149e+01
1.150e+01
1.151e+01
1.152e+01
1.153e+01
1.154e+01
1.155e+01
1.156e+01
1.157e+01
1.158e+01
1.159e+01
1.160e+01
1.161e+01
1.162e+01
1.163e+01
1.164e+01
1.165e+01
1.166e+01
1.167e+01
1.168e+01
1.169e+01
1.170e+01
1.171e+01
1.172e+01
1.173e+01
1.174e+01
1.175e+01
1.176e+01
1.177e+01
1.178e+01
1.179e+01
1.180e+01
1.181e+01
1.182e+01
1.183e+01
1.184e+01
1.185e+01
1.186e+01
1.187e+01
1.188e+01
1.189e+01
1.190e+01
1.191e+01
1.192e+01
1.193e+01
1.194e+01
1.195e+01
1.196e+01
1.197e+01
1.198e+01
1.199e+01

```
      1.200e+01
sp2  0.00000e+00  $ source energy bin probabilities
     1.96197E-06
     1.41801E-05
     4.16849E-05
     3.08240E-04
     7.88179E-04
     1.16006E-03
     1.28737E-03
     1.37849E-03
     1.23091E-03
     1.12163E-03
     1.02437E-03
     9.50239E-04
     8.99029E-04
     8.40999E-04
     7.73475E-04
     7.23781E-04
     6.85395E-04
     6.69185E-04
     6.18914E-04
     5.90066E-04
     5.51483E-04
     5.32665E-04
     5.19882E-04
     5.33717E-04
     4.98786E-04
     4.67041E-04
     4.29100E-04
     3.96669E-04
     3.74930E-04
     3.59461E-04
     3.33708E-04
     3.14449E-04
     3.00028E-04
     2.84882E-04
     2.73403E-04
     2.73510E-04
     2.52890E-04
     2.43429E-04
     2.31306E-04
     2.26546E-04
     2.18575E-04
     2.11949E-04
     2.06104E-04
     2.45860E-04
     1.95042E-04
     1.90972E-04
     1.94119E-04
     2.29546E-04
     1.78539E-04
     1.73742E-04
     1.85446E-04
     1.92074E-03
```

1.19769E-04
1.22915E-04
1.13391E-04
1.13338E-04
1.11317E-04
1.08166E-04
1.07433E-04
1.04706E-04
1.03799E-04
1.05353E-04
9.99214E-05
1.11976E-04
9.63779E-05
9.65487E-05
9.92652E-05
9.19344E-05
9.04809E-05
9.27277E-05
9.34958E-05
8.80126E-05
8.81868E-05
8.69076E-05
8.46708E-05
8.99077E-05
8.30853E-05
8.12531E-05
1.27425E-03
6.62133E-05
6.48075E-05
6.84098E-05
6.32540E-05
7.17949E-05
6.70260E-05
6.67850E-05
8.11892E-05
7.20528E-05
6.20715E-05
6.22544E-05
6.19490E-05
6.21283E-05
5.79033E-05
8.51846E-05
6.68728E-05
6.00377E-05
5.80606E-05
5.76798E-05
6.09817E-05
5.52647E-05
5.41622E-05
5.93919E-05
5.78181E-05
7.58959E-05
5.88579E-05
5.27291E-05

5.90645E-05
5.30814E-05
5.95373E-05
5.18988E-05
5.14341E-05
5.11498E-05
5.32825E-05
1.44235E-04
5.00463E-05
4.90558E-05
1.52731E-03
6.59453E-05
3.88153E-05
4.03549E-05
4.52498E-05
3.85193E-05
3.99588E-05
4.41499E-05
3.87552E-05
4.07180E-05
4.79246E-05
5.33948E-05
3.98129E-05
3.78248E-05
3.84477E-05
3.73281E-05
1.03538E-04
3.75373E-05
4.05995E-05
3.79166E-05
3.56823E-05
5.67167E-05
3.81839E-05
3.54842E-05
4.01176E-05
3.62010E-05
3.93144E-05
3.59155E-05
3.63380E-05
3.50211E-05
3.54406E-05
3.45313E-05
3.50024E-05
4.40636E-05
3.54083E-05
5.02584E-05
5.36512E-05
3.51156E-05
3.50722E-05
3.42268E-05
3.54906E-05
3.32615E-05
3.41046E-05
3.66292E-05

2.28771E-04
3.83834E-05
6.14565E-05
3.72373E-05
8.56006E-05
4.60580E-05
3.26609E-05
4.44589E-05
4.37555E-05
3.73999E-05
4.47951E-05
3.29715E-05
5.76390E-05
4.49060E-05
4.81926E-05
3.22470E-05
3.33124E-05
3.55052E-05
6.55576E-05
3.24768E-05
4.05345E-05
3.17793E-05
5.33319E-05
3.10425E-05
4.21488E-05
4.87819E-05
3.27528E-05
3.07936E-05
3.23394E-05
3.28790E-05
3.12605E-05
3.27495E-05
3.43615E-05
5.63295E-05
3.19193E-05
1.89172E-03
2.76922E-05
6.73688E-05
3.08582E-05
4.11783E-05
3.92834E-05
3.44243E-05
6.35953E-05
7.50973E-05
5.71797E-05
2.64189E-05
2.75108E-05
7.32520E-05
2.69505E-05
6.43628E-05
3.17839E-05
3.89729E-05
2.75275E-05
3.47553E-05

2.62218E-05
6.46961E-05
2.67733E-05
4.15444E-05
2.97223E-05
2.63086E-05
5.49287E-05
2.96791E-05
1.18464E-02
5.58483E-05
1.79745E-05
2.07497E-05
1.02371E-05
8.03256E-06
4.20095E-05
6.15505E-06
5.99731E-06
7.09723E-05
1.97520E-05
5.89930E-06
8.21298E-06
1.31512E-05
1.10105E-05
5.92281E-06
1.49078E-05
1.60364E-05
1.86327E-05
4.35457E-05
1.95338E-05
5.75494E-06
6.05977E-06
6.96483E-06
6.79961E-05
5.41341E-06
3.28767E-05
3.22719E-05
8.18760E-06
1.75824E-05
2.75584E-05
3.26332E-05
2.30725E-05
1.26897E-05
1.97242E-05
8.42458E-06
1.44537E-05
2.35534E-05
5.16848E-06
1.34762E-05
4.72582E-05
1.53202E-05
2.23619E-05
9.83004E-06
1.15908E-05
1.05648E-04

1.52087E-05
8.94933E-06
5.05941E-06
1.33855E-05
1.43428E-05
4.95405E-06
1.57422E-05
2.06105E-05
5.26773E-06
4.55691E-06
6.53533E-06
2.31932E-05
4.62475E-05
3.65370E-05
1.16769E-05
5.69523E-06
8.42855E-05
4.32878E-06
4.13969E-04
5.91861E-05
3.89717E-06
3.90017E-05
4.77619E-06
3.99121E-06
4.46425E-06
3.59201E-06
1.26663E-05
1.30881E-05
2.16741E-05
6.99554E-05
7.01187E-06
6.27763E-05
4.96913E-05
7.54124E-05
9.97161E-06
1.90515E-05
6.20629E-06
5.60013E-06
2.42147E-04
3.40765E-06
9.57001E-06
9.60999E-06
1.61026E-05
6.82711E-05
3.87891E-06
1.09941E-05
3.07339E-06
1.15362E-05
3.16818E-06
2.93778E-06
3.85003E-06
9.17865E-06
8.80257E-06
7.91383E-06

3.17648E-06
3.45842E-06
9.18245E-06
2.19364E-05
5.35594E-06
1.12595E-05
3.02374E-06
1.56967E-05
5.46676E-06
2.23239E-05
3.19201E-06
5.69930E-05
1.90128E-05
3.13447E-06
6.96081E-06
4.26930E-05
5.62078E-06
3.90894E-06
3.31061E-06
5.23397E-06
5.97680E-05
1.31688E-05
2.63319E-06
8.48986E-06
1.65748E-05
9.83422E-06
3.43739E-06
3.03143E-06
3.93427E-05
7.52406E-06
8.02749E-06
1.18479E-04
3.43096E-06
1.42665E-05
7.18568E-05
2.48995E-06
6.18430E-05
4.84509E-05
2.81920E-05
9.63202E-06
1.96805E-05
2.20083E-05
6.28682E-06
2.45706E-06
2.34100E-05
2.86668E-06
1.01342E-05
2.16860E-06
1.61941E-05
2.36057E-06
5.72940E-06
1.57863E-05
2.94855E-05
2.19112E-06

2.30727E-06
2.03632E-05
2.18519E-06
4.20123E-06
5.50319E-06
2.30512E-06
1.34196E-04
2.01523E-06
8.85066E-06
6.13122E-06
1.13966E-05
1.18042E-05
2.15937E-06
2.73382E-06
2.52307E-06
6.54232E-06
3.22999E-06
2.53724E-06
2.23397E-06
6.43282E-06
3.02049E-05
1.08841E-05
8.49246E-05
7.21431E-06
8.50167E-06
3.29865E-06
1.55100E-05
2.08400E-06
7.90660E-06
4.39907E-05
1.82902E-05
2.22273E-06
5.92616E-05
6.03492E-06
1.85356E-06
8.23418E-06
5.72656E-06
3.89654E-05
2.82035E-06
1.83422E-06
1.24853E-05
3.07397E-06
3.20579E-06
3.58208E-06
1.00287E-05
5.39907E-06
2.01482E-06
1.87945E-06
1.98523E-06
1.90862E-06
1.79371E-05
1.97053E-06
1.91115E-06
3.12696E-05

4.66116E-06
1.76614E-06
2.07892E-06
1.64908E-06
1.78888E-06
1.19092E-05
2.76815E-06
3.13259E-06
1.99167E-06
1.74228E-06
4.20090E-06
2.47439E-05
7.09526E-06
2.96504E-06
7.48532E-05
9.21988E-06
2.52140E-06
3.56522E-06
2.19151E-06
5.58148E-06
2.00119E-06
1.23790E-05
3.33731E-05
1.89258E-06
3.24696E-05
1.20280E-05
1.59711E-06
2.75085E-06
2.61240E-05
9.15846E-06
1.74798E-06
4.89894E-05
2.68462E-06
2.79113E-06
1.81383E-06
3.76097E-06
6.91586E-06
2.03891E-06
5.48577E-06
1.13253E-05
1.81239E-06
1.72871E-06
5.14000E-05
1.57214E-05
4.01356E-06
2.08737E-05
3.56990E-06
1.63198E-06
1.64878E-06
3.55792E-06
3.38983E-06
1.73654E-05
1.53945E-05
1.97750E-06

1.96673E-06
1.78973E-06
1.43838E-06
1.62015E-06
8.31340E-06
1.83655E-06
8.64883E-06
1.76331E-06
1.54126E-06
1.24159E-04
1.24527E-04
1.33919E-05
1.39151E-06
2.55991E-04
2.35449E-05
1.34161E-06
4.49068E-06
3.33635E-05
1.34429E-06
1.26406E-06
1.87282E-06
1.39709E-06
1.69027E-06
1.19403E-05
4.05906E-06
1.39483E-06
1.48592E-05
1.78728E-06
3.64395E-06
8.02074E-06
8.50557E-06
1.57013E-05
1.22519E-06
1.14216E-06
1.21531E-06
1.25458E-06
1.56189E-05
1.21532E-06
1.28646E-06
1.33562E-06
3.95578E-05
1.35542E-06
8.92720E-06
2.46808E-06
1.47573E-06
1.77182E-06
2.15086E-06
1.49879E-06
1.61134E-06
1.48796E-06
1.39593E-06
3.34017E-06
1.35362E-06
4.82036E-06

1.36735E-06
1.52045E-06
1.49070E-06
6.81018E-06
1.39476E-06
1.40713E-06
1.56148E-06
2.16080E-06
1.49339E-06
7.66690E-06
1.48160E-06
1.62233E-06
1.41673E-06
1.21672E-04
1.36813E-06
1.77409E-06
1.35504E-06
1.25960E-06
1.40291E-06
1.08279E-06
4.25461E-05
1.32001E-06
2.67056E-05
6.55147E-06
1.34161E-06
5.51929E-06
1.32182E-06
1.28887E-06
1.21202E-06
1.31042E-06
1.22941E-06
1.09459E-06
3.28621E-05
3.83657E-04
1.06681E-06
3.79450E-05
1.16448E-06
6.61604E-06
1.82459E-06
1.32061E-05
1.24776E-06
1.13376E-06
1.15812E-06
1.07390E-06
9.11132E-07
9.97551E-07
1.02493E-06
8.70287E-07
1.86029E-06
9.84757E-07
1.11552E-06
1.08223E-06
8.12856E-05
9.79759E-07

1.09933E-05
9.73826E-07
9.65536E-07
1.47781E-05
9.42338E-07
1.03591E-06
9.84622E-07
1.22905E-06
1.16826E-06
1.07585E-05
1.05096E-06
8.47726E-07
1.61979E-06
4.36545E-06
1.26834E-06
1.23326E-06
6.19137E-05
1.15367E-06
7.93928E-06
1.49408E-03
1.63310E-05
8.97922E-07
7.53451E-07
5.68544E-07
6.36750E-07
5.48349E-07
9.29189E-06
5.84373E-07
1.98456E-06
6.44568E-07
5.88530E-07
5.88413E-07
6.53794E-07
5.75485E-06
3.30911E-05
7.27775E-07
6.18122E-07
7.27436E-07
5.29535E-07
4.12057E-06
1.05132E-06
5.61431E-06
4.92989E-06
4.93508E-07
5.73198E-07
3.82580E-05
1.67975E-06
1.94135E-05
4.45678E-07
6.10526E-07
2.07868E-05
4.66609E-07
1.68148E-06
6.12263E-07

6.24847E-07
5.65411E-07
1.09049E-05
5.78364E-07
6.97045E-07
5.12636E-07
5.39982E-07
5.12304E-07
1.19009E-05
6.14895E-07
5.97238E-07
5.70138E-07
6.25215E-07
5.48461E-07
5.06974E-07
5.72428E-04
3.44256E-04
3.76073E-07
1.44714E-05
3.44479E-07
3.56017E-07
2.93010E-07
4.04045E-07
3.44267E-07
4.09093E-07
2.91966E-06
3.17178E-07
3.05152E-07
2.69782E-06
3.16214E-07
3.72752E-07
3.00445E-07
4.06772E-07
3.33364E-07
3.44160E-07
4.81025E-07
5.21072E-07
1.02064E-06
2.87193E-07
3.61980E-07
5.41205E-07
5.23848E-07
3.07401E-07
5.24483E-07
4.82512E-07
3.93860E-06
4.23130E-07
4.08066E-07
4.63265E-07
1.13378E-05
5.82685E-07
1.68013E-04
2.21630E-07
2.63926E-07

3.32695E-07
2.75758E-07
2.28037E-07
2.89449E-07
2.55348E-07
6.23749E-07
2.22233E-07
2.45878E-07
3.09162E-07
3.16582E-07
9.00964E-07
6.06685E-06
3.27783E-07
2.75003E-07
3.20083E-07
3.08483E-07
3.15296E-07
2.58800E-07
2.10704E-07
1.49212E-05
3.16771E-07
3.04811E-07
2.70705E-07
2.72278E-07
3.62554E-07
2.51420E-07
4.66427E-07
6.33574E-06
2.41250E-07
4.26198E-07
2.63460E-07
3.22536E-07
3.26280E-07
2.63341E-07
2.22691E-07
2.88545E-07
3.86578E-07
2.54636E-06
2.82932E-07
3.25889E-07
3.34721E-07
7.79630E-04
1.83520E-07
1.43098E-07
1.78913E-07
1.52633E-07
1.15098E-07
1.47735E-07
1.55271E-07
1.68943E-07
1.93890E-07
1.51831E-07
1.95743E-07
3.77681E-07

1.62772E-07
1.78655E-06
2.07967E-07
2.26101E-07
1.90063E-07
2.32211E-07
1.74173E-07
1.55956E-07
1.74420E-07
2.91614E-05
2.46444E-05
1.89179E-07
1.73391E-07
1.51995E-07
1.88796E-07
1.01173E-05
1.88511E-07
1.52301E-07
5.92900E-05
1.88220E-07
1.36317E-07
2.10834E-07
1.63374E-07
2.86038E-07
2.25470E-07
6.16863E-04
5.44531E-08
2.93544E-07
6.16408E-08
5.67192E-08
7.85130E-08
8.11151E-08
3.38388E-08
8.12122E-08
8.89571E-08
8.13342E-08
4.87447E-08
2.30129E-08
7.66131E-08
6.81967E-07
1.22963E-07
8.02914E-08
3.82133E-08
2.50000E-08
5.86292E-08
1.23770E-07
2.50956E-08
6.66284E-08
3.52440E-08
4.86187E-08
1.66842E-08
2.30427E-08
4.43797E-08
3.76200E-08

5.69291E-08
2.86296E-08
2.92506E-08
3.55445E-08
1.46435E-07
1.52201E-08
8.78678E-08
1.18869E-08
2.20209E-07
5.02001E-08
4.99811E-08
4.74262E-08
3.29743E-08
3.24063E-08
4.23708E-08
2.78223E-08
2.57271E-08
3.69649E-08
3.34450E-08
3.10747E-08
3.02975E-08
5.13435E-08
4.07855E-08
7.25376E-08
1.03204E-07
5.03001E-08
1.87712E-08
5.74214E-09
3.10242E-07
1.03320E-07
4.32742E-08
3.12486E-08
1.50000E-08
1.76348E-08
2.41603E-08
5.46176E-08
1.58658E-08
2.52201E-08
2.02201E-08
4.86500E-06
3.38816E-07
2.52201E-08
1.35192E-07
4.69550E-07
1.20000E-07
5.34879E-07
7.09373E-08
1.13383E-07
1.43142E-07
2.07217E-04
5.57596E-08
5.50000E-08
6.52201E-08
4.19289E-08

3.74147E-08
6.11696E-08
1.52202E-08
1.52201E-08
1.11112E-08
1.63314E-08
9.99996E-09
0.00000E+00
1.02202E-08
1.00000E-08
1.00417E-08
1.49999E-08
0.00000E+00
0.00000E+00
9.99996E-09
1.49999E-08
5.22015E-09
1.52201E-08
5.00000E-09
0.00000E+00
9.99996E-09
1.02504E-08
4.99996E-09
5.00000E-09
0.00000E+00
2.95000E-07
1.89672E-06
6.02201E-08
3.63825E-08
1.49999E-08
5.00000E-09
2.02201E-08
4.99996E-09
3.52201E-08
4.99996E-09
1.00000E-08
5.00000E-09
1.03500E-06
9.99992E-09
9.99992E-09
1.00000E-08
5.00000E-09
1.99999E-08
1.00000E-08
9.99996E-09
0.00000E+00
4.99996E-09
0.00000E+00
0.00000E+00
9.99992E-09
4.99996E-09
4.99996E-09
3.00000E-08
9.99996E-09

0.00000E+00
9.99996E-09
4.99996E-09
2.04403E-08
0.00000E+00
4.99996E-09
5.00000E-09
6.73633E-08
5.22015E-09
1.52201E-08
2.00000E-08
5.00000E-09
9.99996E-09
3.35000E-06
5.00000E-09
9.99996E-09
1.02202E-08
0.00000E+00
5.00000E-09
2.00000E-08
0.00000E+00
1.02202E-08
0.00000E+00
9.99996E-09
5.00000E-09
4.99996E-09
0.00000E+00
5.00000E-09
4.99996E-09
4.99996E-09
2.02201E-08
5.00000E-09
5.00000E-09
1.02201E-08
0.00000E+00
4.99996E-09
4.99996E-09
0.00000E+00
0.00000E+00
0.00000E+00
1.04055E-08
1.02202E-08
0.00000E+00
5.00000E-09
5.22015E-09
7.00000E-08
1.04403E-08
0.00000E+00
4.99996E-09
1.50000E-08
4.99996E-09
0.00000E+00
2.04403E-08
0.00000E+00

1.00000E-08
3.79590E-07
0.00000E+00
0.00000E+00
0.00000E+00
5.00000E-09
1.00000E-08
5.00000E-09
1.50000E-08
8.00000E-08
5.00000E-09
1.02201E-08
1.52201E-08
9.99996E-09
0.00000E+00
1.52201E-08
0.00000E+00
1.52201E-08
9.99996E-09
5.00000E-09
1.00000E-08
5.00000E-09
1.00000E-08
0.00000E+00
2.74515E-08
1.01652E-08
1.52201E-08
1.50000E-08
1.50000E-08
1.00000E-08
5.00000E-09
4.99996E-09
0.00000E+00
4.99996E-09
5.03143E-08
0.00000E+00
4.99996E-09
1.00000E-08
4.99996E-09
0.00000E+00
5.00000E-09
1.52201E-08
1.50000E-08
0.00000E+00
0.00000E+00
1.02201E-08
1.00000E-08
0.00000E+00
0.00000E+00
1.50000E-08
2.00000E-08
9.99996E-09
5.00000E-09
1.52201E-08

0.00000E+00
9.99996E-09
4.99996E-09
5.00000E-09
0.00000E+00
9.99996E-09
0.00000E+00
5.00000E-09
0.00000E+00
0.00000E+00
5.00000E-09
1.00000E-08
5.00000E-09
0.00000E+00
2.00000E-08
1.50000E-08
0.00000E+00
4.99996E-09
5.00000E-09
1.00000E-08
0.00000E+00
5.00000E-09
2.99999E-08
5.00000E-09
0.00000E+00
0.00000E+00
5.00000E-09
5.00000E-09
5.00000E-09
5.00000E-09
4.99996E-09
5.00000E-09
5.00000E-09
0.00000E+00
1.00000E-08
2.00000E-08
5.50000E-07
0.00000E+00
1.00000E-08
1.52201E-08
1.50000E-08
5.00000E-09
0.00000E+00
0.00000E+00
0.00000E+00
5.00000E-09
1.50000E-08
5.00000E-09
5.00000E-09
0.00000E+00
2.00000E-08
9.99992E-09
5.00000E-09
0.00000E+00

1.00000E-08
0.00000E+00
5.00000E-09
4.99996E-09
2.45000E-07
5.00000E-09
1.00000E-08
1.50000E-08
5.22015E-09
5.00000E-09
1.00000E-08
2.50000E-08
0.00000E+00
5.00000E-09
0.00000E+00
0.00000E+00
1.00000E-08
0.00000E+00
1.02201E-08
1.50000E-08
0.00000E+00
0.00000E+00
0.00000E+00
0.00000E+00
1.00000E-08
4.99996E-09
0.00000E+00
0.00000E+00
1.00000E-08
1.00000E-08
5.22015E-09
0.00000E+00
5.00000E-09
1.50000E-08
0.00000E+00
5.00000E-09
0.00000E+00
0.00000E+00
5.00000E-09
9.99996E-09
1.50000E-08
5.00000E-09
4.99996E-09
0.00000E+00
0.00000E+00
5.00000E-09
1.50000E-08
0.00000E+00
0.00000E+00
0.00000E+00
5.00000E-09
0.00000E+00
1.00000E-08
1.00000E-08

1.00000E-08
5.00000E-09
0.00000E+00
0.00000E+00
5.00000E-09
5.00000E-09
0.00000E+00
5.00000E-09
1.00000E-08
1.00000E-08
5.00000E-09
1.00000E-08
5.00000E-09
5.00000E-09
9.99996E-09
0.00000E+00
5.00000E-09
5.00000E-09
5.00000E-09
0.00000E+00
0.00000E+00
5.00000E-09
1.00000E-08
0.00000E+00
0.00000E+00
1.50000E-08
0.00000E+00
5.00000E-09
0.00000E+00
1.00000E-08
1.00000E-08
0.00000E+00
5.00000E-09
5.00000E-09
1.00000E-08
0.00000E+00
0.00000E+00
0.00000E+00
0.00000E+00
0.00000E+00
0.00000E+00
5.00000E-09
0.00000E+00
0.00000E+00
0.00000E+00
1.50000E-08
1.50000E-08
5.00000E-09
5.00000E-09
0.00000E+00
5.22015E-09
5.00000E-09
0.00000E+00
0.00000E+00

```
     0.00000E+00
     0.00000E+00
     1.00000E-08
     5.00000E-09
     0.00000E+00
     0.00000E+00
     0.00000E+00
     5.00000E-09
     0.00000E+00
     1.00000E-08
     5.00000E-09
     0.00000E+00
     0.00000E+00
     0.00000E+00
cut:p j 0.001                        $ Photon cut-off energy 1.0 keV
f8:p  1                              $ Pulse height tally defined for cell
1 (NaI detector)
e8    0.0 1.0e-5 0.01 1198i 12.0     $ Energy binning
ft8   geb  0.016410  0.076003  0.0  $ Tally treatment with GEB
nps   2.0e+8                         $ Number of histories
```

8. SiO2 + 150 ppm Gd / GEB / 10 cm seawater layer

```
c     SiO2 + 150 ppm Gd
c     Pulse 'OFF'
c     Using re-normalized f1 data
c     Particle weight determined from the total of f1 data
c
c
c   Problem Cells
c
1  1 -3.67      1  -2  -3    imp:p=1     $ crystal NaI
2  2 -1.22e-3  -4  #1        imp:p=1     $ Inner world (air)
3  0            4            imp:p=0     $ Outer world (void)

c
c   Problem Surfaces
c
1  pz  0.0
2  pz  7.62
3  cz  3.81
4  so  100.0

c
c   Problem data cards
c
mode p                      $ photon only problem
m1   11023.80c   1
     53127.80c   1
m2    7014.80c   0.8
      8016.80c   0.2
c
c   Source definition -- energy is now a prob. dist.
c
sdef erg=d2  rad=d1  pos=0.0 0.0 -3.0  vec=0 0 1  dir=1  ext=0
     axs=0 0 1 wgt=0.0761525
si1   0  3.81    $ radial sampling range: 0 to 3.81
sp1  -21 1       $ uniform sampling over the disk area
si2 h 0.000e+00  $ source energy binning / histogram
      1.000e-02
      2.000e-02
      3.000e-02
      4.000e-02
      5.000e-02
      6.000e-02
      7.000e-02
      8.000e-02
      9.000e-02
      1.000e-01
      1.100e-01
      1.200e-01
      1.300e-01
      1.400e-01
      1.500e-01
      1.600e-01
      1.700e-01
```

1.800e-01
1.900e-01
2.000e-01
2.100e-01
2.200e-01
2.300e-01
2.400e-01
2.500e-01
2.600e-01
2.700e-01
2.800e-01
2.900e-01
3.000e-01
3.100e-01
3.200e-01
3.300e-01
3.400e-01
3.500e-01
3.600e-01
3.700e-01
3.800e-01
3.900e-01
4.000e-01
4.100e-01
4.200e-01
4.300e-01
4.400e-01
4.500e-01
4.600e-01
4.700e-01
4.800e-01
4.900e-01
5.000e-01
5.100e-01
5.200e-01
5.300e-01
5.400e-01
5.500e-01
5.600e-01
5.700e-01
5.800e-01
5.900e-01
6.000e-01
6.100e-01
6.200e-01
6.300e-01
6.400e-01
6.500e-01
6.600e-01
6.700e-01
6.800e-01
6.900e-01
7.000e-01
7.100e-01

```
7.200e-01
7.300e-01
7.400e-01
7.500e-01
7.600e-01
7.700e-01
7.800e-01
7.900e-01
8.000e-01
8.100e-01
8.200e-01
8.300e-01
8.400e-01
8.500e-01
8.600e-01
8.700e-01
8.800e-01
8.900e-01
9.000e-01
9.100e-01
9.200e-01
9.300e-01
9.400e-01
9.500e-01
9.600e-01
9.700e-01
9.800e-01
9.900e-01
1.000e+00
1.010e+00
1.020e+00
1.030e+00
1.040e+00
1.050e+00
1.060e+00
1.070e+00
1.080e+00
1.090e+00
1.100e+00
1.110e+00
1.120e+00
1.130e+00
1.140e+00
1.150e+00
1.160e+00
1.170e+00
1.180e+00
1.190e+00
1.200e+00
1.210e+00
1.220e+00
1.230e+00
1.240e+00
1.250e+00
```

1.260e+00
1.270e+00
1.280e+00
1.290e+00
1.300e+00
1.310e+00
1.320e+00
1.330e+00
1.340e+00
1.350e+00
1.360e+00
1.370e+00
1.380e+00
1.390e+00
1.400e+00
1.410e+00
1.420e+00
1.430e+00
1.440e+00
1.450e+00
1.460e+00
1.470e+00
1.480e+00
1.490e+00
1.500e+00
1.510e+00
1.520e+00
1.530e+00
1.540e+00
1.550e+00
1.560e+00
1.570e+00
1.580e+00
1.590e+00
1.600e+00
1.610e+00
1.620e+00
1.630e+00
1.640e+00
1.650e+00
1.660e+00
1.670e+00
1.680e+00
1.690e+00
1.700e+00
1.710e+00
1.720e+00
1.730e+00
1.740e+00
1.750e+00
1.760e+00
1.770e+00
1.780e+00
1.790e+00

1.800e+00
1.810e+00
1.820e+00
1.830e+00
1.840e+00
1.850e+00
1.860e+00
1.870e+00
1.880e+00
1.890e+00
1.900e+00
1.910e+00
1.920e+00
1.930e+00
1.940e+00
1.950e+00
1.960e+00
1.970e+00
1.980e+00
1.990e+00
2.000e+00
2.010e+00
2.020e+00
2.030e+00
2.040e+00
2.050e+00
2.060e+00
2.070e+00
2.080e+00
2.090e+00
2.100e+00
2.110e+00
2.120e+00
2.130e+00
2.140e+00
2.150e+00
2.160e+00
2.170e+00
2.180e+00
2.190e+00
2.200e+00
2.210e+00
2.220e+00
2.230e+00
2.240e+00
2.250e+00
2.260e+00
2.270e+00
2.280e+00
2.290e+00
2.300e+00
2.310e+00
2.320e+00
2.330e+00

2.340e+00
2.350e+00
2.360e+00
2.370e+00
2.380e+00
2.390e+00
2.400e+00
2.410e+00
2.420e+00
2.430e+00
2.440e+00
2.450e+00
2.460e+00
2.470e+00
2.480e+00
2.490e+00
2.500e+00
2.510e+00
2.520e+00
2.530e+00
2.540e+00
2.550e+00
2.560e+00
2.570e+00
2.580e+00
2.590e+00
2.600e+00
2.610e+00
2.620e+00
2.630e+00
2.640e+00
2.650e+00
2.660e+00
2.670e+00
2.680e+00
2.690e+00
2.700e+00
2.710e+00
2.720e+00
2.730e+00
2.740e+00
2.750e+00
2.760e+00
2.770e+00
2.780e+00
2.790e+00
2.800e+00
2.810e+00
2.820e+00
2.830e+00
2.840e+00
2.850e+00
2.860e+00
2.870e+00

2.880e+00
2.890e+00
2.900e+00
2.910e+00
2.920e+00
2.930e+00
2.940e+00
2.950e+00
2.960e+00
2.970e+00
2.980e+00
2.990e+00
3.000e+00
3.010e+00
3.020e+00
3.030e+00
3.040e+00
3.050e+00
3.060e+00
3.070e+00
3.080e+00
3.090e+00
3.100e+00
3.110e+00
3.120e+00
3.130e+00
3.140e+00
3.150e+00
3.160e+00
3.170e+00
3.180e+00
3.190e+00
3.200e+00
3.210e+00
3.220e+00
3.230e+00
3.240e+00
3.250e+00
3.260e+00
3.270e+00
3.280e+00
3.290e+00
3.300e+00
3.310e+00
3.320e+00
3.330e+00
3.340e+00
3.350e+00
3.360e+00
3.370e+00
3.380e+00
3.390e+00
3.400e+00
3.410e+00

3.420e+00
3.430e+00
3.440e+00
3.450e+00
3.460e+00
3.470e+00
3.480e+00
3.490e+00
3.500e+00
3.510e+00
3.520e+00
3.530e+00
3.540e+00
3.550e+00
3.560e+00
3.570e+00
3.580e+00
3.590e+00
3.600e+00
3.610e+00
3.620e+00
3.630e+00
3.640e+00
3.650e+00
3.660e+00
3.670e+00
3.680e+00
3.690e+00
3.700e+00
3.710e+00
3.720e+00
3.730e+00
3.740e+00
3.750e+00
3.760e+00
3.770e+00
3.780e+00
3.790e+00
3.800e+00
3.810e+00
3.820e+00
3.830e+00
3.840e+00
3.850e+00
3.860e+00
3.870e+00
3.880e+00
3.890e+00
3.900e+00
3.910e+00
3.920e+00
3.930e+00
3.940e+00
3.950e+00

3.960e+00
3.970e+00
3.980e+00
3.990e+00
4.000e+00
4.010e+00
4.020e+00
4.030e+00
4.040e+00
4.050e+00
4.060e+00
4.070e+00
4.080e+00
4.090e+00
4.100e+00
4.110e+00
4.120e+00
4.130e+00
4.140e+00
4.150e+00
4.160e+00
4.170e+00
4.180e+00
4.190e+00
4.200e+00
4.210e+00
4.220e+00
4.230e+00
4.240e+00
4.250e+00
4.260e+00
4.270e+00
4.280e+00
4.290e+00
4.300e+00
4.310e+00
4.320e+00
4.330e+00
4.340e+00
4.350e+00
4.360e+00
4.370e+00
4.380e+00
4.390e+00
4.400e+00
4.410e+00
4.420e+00
4.430e+00
4.440e+00
4.450e+00
4.460e+00
4.470e+00
4.480e+00
4.490e+00

4.500e+00
4.510e+00
4.520e+00
4.530e+00
4.540e+00
4.550e+00
4.560e+00
4.570e+00
4.580e+00
4.590e+00
4.600e+00
4.610e+00
4.620e+00
4.630e+00
4.640e+00
4.650e+00
4.660e+00
4.670e+00
4.680e+00
4.690e+00
4.700e+00
4.710e+00
4.720e+00
4.730e+00
4.740e+00
4.750e+00
4.760e+00
4.770e+00
4.780e+00
4.790e+00
4.800e+00
4.810e+00
4.820e+00
4.830e+00
4.840e+00
4.850e+00
4.860e+00
4.870e+00
4.880e+00
4.890e+00
4.900e+00
4.910e+00
4.920e+00
4.930e+00
4.940e+00
4.950e+00
4.960e+00
4.970e+00
4.980e+00
4.990e+00
5.000e+00
5.010e+00
5.020e+00
5.030e+00

5.040e+00
5.050e+00
5.060e+00
5.070e+00
5.080e+00
5.090e+00
5.100e+00
5.110e+00
5.120e+00
5.130e+00
5.140e+00
5.150e+00
5.160e+00
5.170e+00
5.180e+00
5.190e+00
5.200e+00
5.210e+00
5.220e+00
5.230e+00
5.240e+00
5.250e+00
5.260e+00
5.270e+00
5.280e+00
5.290e+00
5.300e+00
5.310e+00
5.320e+00
5.330e+00
5.340e+00
5.350e+00
5.360e+00
5.370e+00
5.380e+00
5.390e+00
5.400e+00
5.410e+00
5.420e+00
5.430e+00
5.440e+00
5.450e+00
5.460e+00
5.470e+00
5.480e+00
5.490e+00
5.500e+00
5.510e+00
5.520e+00
5.530e+00
5.540e+00
5.550e+00
5.560e+00
5.570e+00

5.580e+00
5.590e+00
5.600e+00
5.610e+00
5.620e+00
5.630e+00
5.640e+00
5.650e+00
5.660e+00
5.670e+00
5.680e+00
5.690e+00
5.700e+00
5.710e+00
5.720e+00
5.730e+00
5.740e+00
5.750e+00
5.760e+00
5.770e+00
5.780e+00
5.790e+00
5.800e+00
5.810e+00
5.820e+00
5.830e+00
5.840e+00
5.850e+00
5.860e+00
5.870e+00
5.880e+00
5.890e+00
5.900e+00
5.910e+00
5.920e+00
5.930e+00
5.940e+00
5.950e+00
5.960e+00
5.970e+00
5.980e+00
5.990e+00
6.000e+00
6.010e+00
6.020e+00
6.030e+00
6.040e+00
6.050e+00
6.060e+00
6.070e+00
6.080e+00
6.090e+00
6.100e+00
6.110e+00

6.120e+00
6.130e+00
6.140e+00
6.150e+00
6.160e+00
6.170e+00
6.180e+00
6.190e+00
6.200e+00
6.210e+00
6.220e+00
6.230e+00
6.240e+00
6.250e+00
6.260e+00
6.270e+00
6.280e+00
6.290e+00
6.300e+00
6.310e+00
6.320e+00
6.330e+00
6.340e+00
6.350e+00
6.360e+00
6.370e+00
6.380e+00
6.390e+00
6.400e+00
6.410e+00
6.420e+00
6.430e+00
6.440e+00
6.450e+00
6.460e+00
6.470e+00
6.480e+00
6.490e+00
6.500e+00
6.510e+00
6.520e+00
6.530e+00
6.540e+00
6.550e+00
6.560e+00
6.570e+00
6.580e+00
6.590e+00
6.600e+00
6.610e+00
6.620e+00
6.630e+00
6.640e+00
6.650e+00

6.660e+00
6.670e+00
6.680e+00
6.690e+00
6.700e+00
6.710e+00
6.720e+00
6.730e+00
6.740e+00
6.750e+00
6.760e+00
6.770e+00
6.780e+00
6.790e+00
6.800e+00
6.810e+00
6.820e+00
6.830e+00
6.840e+00
6.850e+00
6.860e+00
6.870e+00
6.880e+00
6.890e+00
6.900e+00
6.910e+00
6.920e+00
6.930e+00
6.940e+00
6.950e+00
6.960e+00
6.970e+00
6.980e+00
6.990e+00
7.000e+00
7.010e+00
7.020e+00
7.030e+00
7.040e+00
7.050e+00
7.060e+00
7.070e+00
7.080e+00
7.090e+00
7.100e+00
7.110e+00
7.120e+00
7.130e+00
7.140e+00
7.150e+00
7.160e+00
7.170e+00
7.180e+00
7.190e+00

7.200e+00
7.210e+00
7.220e+00
7.230e+00
7.240e+00
7.250e+00
7.260e+00
7.270e+00
7.280e+00
7.290e+00
7.300e+00
7.310e+00
7.320e+00
7.330e+00
7.340e+00
7.350e+00
7.360e+00
7.370e+00
7.380e+00
7.390e+00
7.400e+00
7.410e+00
7.420e+00
7.430e+00
7.440e+00
7.450e+00
7.460e+00
7.470e+00
7.480e+00
7.490e+00
7.500e+00
7.510e+00
7.520e+00
7.530e+00
7.540e+00
7.550e+00
7.560e+00
7.570e+00
7.580e+00
7.590e+00
7.600e+00
7.610e+00
7.620e+00
7.630e+00
7.640e+00
7.650e+00
7.660e+00
7.670e+00
7.680e+00
7.690e+00
7.700e+00
7.710e+00
7.720e+00
7.730e+00

7.740e+00
7.750e+00
7.760e+00
7.770e+00
7.780e+00
7.790e+00
7.800e+00
7.810e+00
7.820e+00
7.830e+00
7.840e+00
7.850e+00
7.860e+00
7.870e+00
7.880e+00
7.890e+00
7.900e+00
7.910e+00
7.920e+00
7.930e+00
7.940e+00
7.950e+00
7.960e+00
7.970e+00
7.980e+00
7.990e+00
8.000e+00
8.010e+00
8.020e+00
8.030e+00
8.040e+00
8.050e+00
8.060e+00
8.070e+00
8.080e+00
8.090e+00
8.100e+00
8.110e+00
8.120e+00
8.130e+00
8.140e+00
8.150e+00
8.160e+00
8.170e+00
8.180e+00
8.190e+00
8.200e+00
8.210e+00
8.220e+00
8.230e+00
8.240e+00
8.250e+00
8.260e+00
8.270e+00

8.280e+00
8.290e+00
8.300e+00
8.310e+00
8.320e+00
8.330e+00
8.340e+00
8.350e+00
8.360e+00
8.370e+00
8.380e+00
8.390e+00
8.400e+00
8.410e+00
8.420e+00
8.430e+00
8.440e+00
8.450e+00
8.460e+00
8.470e+00
8.480e+00
8.490e+00
8.500e+00
8.510e+00
8.520e+00
8.530e+00
8.540e+00
8.550e+00
8.560e+00
8.570e+00
8.580e+00
8.590e+00
8.600e+00
8.610e+00
8.620e+00
8.630e+00
8.640e+00
8.650e+00
8.660e+00
8.670e+00
8.680e+00
8.690e+00
8.700e+00
8.710e+00
8.720e+00
8.730e+00
8.740e+00
8.750e+00
8.760e+00
8.770e+00
8.780e+00
8.790e+00
8.800e+00
8.810e+00

8.820e+00
8.830e+00
8.840e+00
8.850e+00
8.860e+00
8.870e+00
8.880e+00
8.890e+00
8.900e+00
8.910e+00
8.920e+00
8.930e+00
8.940e+00
8.950e+00
8.960e+00
8.970e+00
8.980e+00
8.990e+00
9.000e+00
9.010e+00
9.020e+00
9.030e+00
9.040e+00
9.050e+00
9.060e+00
9.070e+00
9.080e+00
9.090e+00
9.100e+00
9.110e+00
9.120e+00
9.130e+00
9.140e+00
9.150e+00
9.160e+00
9.170e+00
9.180e+00
9.190e+00
9.200e+00
9.210e+00
9.220e+00
9.230e+00
9.240e+00
9.250e+00
9.260e+00
9.270e+00
9.280e+00
9.290e+00
9.300e+00
9.310e+00
9.320e+00
9.330e+00
9.340e+00
9.350e+00

9.360e+00
9.370e+00
9.380e+00
9.390e+00
9.400e+00
9.410e+00
9.420e+00
9.430e+00
9.440e+00
9.450e+00
9.460e+00
9.470e+00
9.480e+00
9.490e+00
9.500e+00
9.510e+00
9.520e+00
9.530e+00
9.540e+00
9.550e+00
9.560e+00
9.570e+00
9.580e+00
9.590e+00
9.600e+00
9.610e+00
9.620e+00
9.630e+00
9.640e+00
9.650e+00
9.660e+00
9.670e+00
9.680e+00
9.690e+00
9.700e+00
9.710e+00
9.720e+00
9.730e+00
9.740e+00
9.750e+00
9.760e+00
9.770e+00
9.780e+00
9.790e+00
9.800e+00
9.810e+00
9.820e+00
9.830e+00
9.840e+00
9.850e+00
9.860e+00
9.870e+00
9.880e+00
9.890e+00

9.900e+00
9.910e+00
9.920e+00
9.930e+00
9.940e+00
9.950e+00
9.960e+00
9.970e+00
9.980e+00
9.990e+00
1.000e+01
1.001e+01
1.002e+01
1.003e+01
1.004e+01
1.005e+01
1.006e+01
1.007e+01
1.008e+01
1.009e+01
1.010e+01
1.011e+01
1.012e+01
1.013e+01
1.014e+01
1.015e+01
1.016e+01
1.017e+01
1.018e+01
1.019e+01
1.020e+01
1.021e+01
1.022e+01
1.023e+01
1.024e+01
1.025e+01
1.026e+01
1.027e+01
1.028e+01
1.029e+01
1.030e+01
1.031e+01
1.032e+01
1.033e+01
1.034e+01
1.035e+01
1.036e+01
1.037e+01
1.038e+01
1.039e+01
1.040e+01
1.041e+01
1.042e+01
1.043e+01

1.044e+01
1.045e+01
1.046e+01
1.047e+01
1.048e+01
1.049e+01
1.050e+01
1.051e+01
1.052e+01
1.053e+01
1.054e+01
1.055e+01
1.056e+01
1.057e+01
1.058e+01
1.059e+01
1.060e+01
1.061e+01
1.062e+01
1.063e+01
1.064e+01
1.065e+01
1.066e+01
1.067e+01
1.068e+01
1.069e+01
1.070e+01
1.071e+01
1.072e+01
1.073e+01
1.074e+01
1.075e+01
1.076e+01
1.077e+01
1.078e+01
1.079e+01
1.080e+01
1.081e+01
1.082e+01
1.083e+01
1.084e+01
1.085e+01
1.086e+01
1.087e+01
1.088e+01
1.089e+01
1.090e+01
1.091e+01
1.092e+01
1.093e+01
1.094e+01
1.095e+01
1.096e+01
1.097e+01

1.098e+01
1.099e+01
1.100e+01
1.101e+01
1.102e+01
1.103e+01
1.104e+01
1.105e+01
1.106e+01
1.107e+01
1.108e+01
1.109e+01
1.110e+01
1.111e+01
1.112e+01
1.113e+01
1.114e+01
1.115e+01
1.116e+01
1.117e+01
1.118e+01
1.119e+01
1.120e+01
1.121e+01
1.122e+01
1.123e+01
1.124e+01
1.125e+01
1.126e+01
1.127e+01
1.128e+01
1.129e+01
1.130e+01
1.131e+01
1.132e+01
1.133e+01
1.134e+01
1.135e+01
1.136e+01
1.137e+01
1.138e+01
1.139e+01
1.140e+01
1.141e+01
1.142e+01
1.143e+01
1.144e+01
1.145e+01
1.146e+01
1.147e+01
1.148e+01
1.149e+01
1.150e+01
1.151e+01

```
      1.152e+01
      1.153e+01
      1.154e+01
      1.155e+01
      1.156e+01
      1.157e+01
      1.158e+01
      1.159e+01
      1.160e+01
      1.161e+01
      1.162e+01
      1.163e+01
      1.164e+01
      1.165e+01
      1.166e+01
      1.167e+01
      1.168e+01
      1.169e+01
      1.170e+01
      1.171e+01
      1.172e+01
      1.173e+01
      1.174e+01
      1.175e+01
      1.176e+01
      1.177e+01
      1.178e+01
      1.179e+01
      1.180e+01
      1.181e+01
      1.182e+01
      1.183e+01
      1.184e+01
      1.185e+01
      1.186e+01
      1.187e+01
      1.188e+01
      1.189e+01
      1.190e+01
      1.191e+01
      1.192e+01
      1.193e+01
      1.194e+01
      1.195e+01
      1.196e+01
      1.197e+01
      1.198e+01
      1.199e+01
      1.200e+01
sp2  0.00000e+00  $ source energy bin probabilities
     2.27190E-06
     1.68498E-05
     4.71696E-05
     3.79147E-04
```

1.00311E-03
1.49061E-03
1.65536E-03
1.77453E-03
1.58607E-03
1.43296E-03
1.30989E-03
1.21218E-03
1.13789E-03
1.05423E-03
9.74134E-04
9.03320E-04
8.46625E-04
8.24894E-04
7.61940E-04
7.24622E-04
6.78806E-04
6.52499E-04
6.39895E-04
6.45114E-04
6.03266E-04
5.63007E-04
5.18942E-04
4.81772E-04
4.54810E-04
4.36669E-04
4.05890E-04
3.81990E-04
3.65172E-04
3.48183E-04
3.33781E-04
3.32451E-04
3.07903E-04
2.95582E-04
2.83934E-04
2.75988E-04
2.68328E-04
2.58938E-04
2.51305E-04
2.91451E-04
2.40615E-04
2.35299E-04
2.36518E-04
2.71495E-04
2.17894E-04
2.13939E-04
2.23802E-04
2.05974E-03
1.54588E-04
1.57486E-04
1.45864E-04
1.44398E-04
1.42277E-04
1.38940E-04

1.37740E-04
1.34449E-04
1.32670E-04
1.34945E-04
1.27959E-04
1.41110E-04
1.25193E-04
1.24339E-04
1.26945E-04
1.20781E-04
1.17617E-04
1.18948E-04
1.19602E-04
1.15634E-04
1.14350E-04
1.11945E-04
1.10278E-04
1.15620E-04
1.09050E-04
1.06432E-04
1.29689E-03
9.13366E-05
8.90506E-05
9.28952E-05
8.80243E-05
9.55516E-05
8.96407E-05
9.10258E-05
1.05493E-04
9.40622E-05
8.51119E-05
8.47733E-05
8.51918E-05
8.90066E-05
8.51589E-05
1.15150E-04
9.77545E-05
9.11574E-05
9.14406E-05
9.32904E-05
9.57814E-05
8.93363E-05
8.60594E-05
8.97588E-05
8.56620E-05
1.02448E-04
8.36471E-05
7.77099E-05
8.25064E-05
7.70374E-05
8.12201E-05
7.46656E-05
7.35611E-05
7.29758E-05

7.35505E-05
1.64069E-04
7.08880E-05
6.95988E-05
1.54252E-03
8.93079E-05
6.24951E-05
6.52191E-05
7.02269E-05
6.31794E-05
6.48328E-05
6.83813E-05
6.29667E-05
6.38245E-05
7.12227E-05
7.29639E-05
6.23454E-05
5.76094E-05
5.86681E-05
5.82000E-05
1.21748E-04
5.66689E-05
6.09944E-05
5.79466E-05
5.44584E-05
7.52089E-05
5.70063E-05
5.34774E-05
5.93965E-05
5.45027E-05
5.88699E-05
5.69793E-05
5.76791E-05
5.58323E-05
5.54953E-05
5.54305E-05
5.54513E-05
6.37259E-05
5.54578E-05
6.92830E-05
7.17675E-05
5.35567E-05
5.27541E-05
5.17491E-05
5.33307E-05
5.01056E-05
5.10216E-05
5.37365E-05
2.45179E-04
5.44202E-05
7.75402E-05
5.34613E-05
1.02367E-04
6.22826E-05

4.96177E-05
6.11270E-05
6.08756E-05
5.37583E-05
6.11720E-05
5.01940E-05
7.38742E-05
6.02803E-05
6.36990E-05
4.77985E-05
4.98950E-05
5.25109E-05
8.17638E-05
4.84116E-05
5.70547E-05
4.75814E-05
6.90881E-05
4.71405E-05
5.89696E-05
6.46005E-05
4.88690E-05
4.74777E-05
4.83946E-05
4.90550E-05
4.65300E-05
4.75641E-05
4.95994E-05
7.16233E-05
4.80207E-05
1.90426E-03
4.32008E-05
8.21548E-05
4.52042E-05
5.62093E-05
5.31842E-05
4.96703E-05
7.83857E-05
8.94209E-05
7.20766E-05
4.13682E-05
4.31875E-05
8.93042E-05
4.27650E-05
7.43249E-05
4.65418E-05
5.34933E-05
4.27154E-05
5.01752E-05
4.06488E-05
8.01762E-05
4.18908E-05
5.70234E-05
4.43163E-05
4.17291E-05

6.86370E-05
4.44321E-05
1.18109E-02
7.00275E-05
3.28007E-05
3.48799E-05
2.39764E-05
2.22475E-05
5.61298E-05
2.02073E-05
2.01221E-05
8.40784E-05
3.39523E-05
1.99491E-05
2.17915E-05
2.75111E-05
2.49306E-05
1.94705E-05
2.79792E-05
2.95373E-05
3.18360E-05
5.70579E-05
3.21565E-05
1.88623E-05
1.94063E-05
2.02646E-05
8.11068E-05
1.84569E-05
4.57928E-05
4.52005E-05
2.15295E-05
2.97176E-05
3.97769E-05
4.63649E-05
3.58557E-05
2.57519E-05
3.23364E-05
2.07210E-05
2.74286E-05
3.65337E-05
1.73616E-05
2.63885E-05
5.98520E-05
2.76831E-05
3.45269E-05
2.19279E-05
2.40739E-05
1.17476E-04
2.76794E-05
2.12884E-05
1.80436E-05
2.59600E-05
2.61893E-05
1.79173E-05

2.74469E-05
3.30678E-05
1.71212E-05
1.66463E-05
1.74469E-05
3.52259E-05
5.80146E-05
4.77068E-05
2.30482E-05
1.76986E-05
9.53538E-05
1.47856E-05
4.24476E-04
7.02204E-05
1.54641E-05
5.02940E-05
1.59974E-05
1.47256E-05
1.58470E-05
1.52291E-05
2.31082E-05
2.35819E-05
3.23377E-05
7.98178E-05
1.73684E-05
7.29837E-05
5.98928E-05
8.57843E-05
2.06469E-05
2.94541E-05
1.68271E-05
1.58788E-05
2.53206E-04
1.40452E-05
1.94204E-05
1.89953E-05
2.56484E-05
7.84730E-05
1.47400E-05
2.10961E-05
1.37521E-05
2.15238E-05
1.33046E-05
1.28901E-05
1.34852E-05
1.88807E-05
1.82448E-05
1.71571E-05
1.35094E-05
1.29110E-05
1.91957E-05
3.12374E-05
1.47151E-05
2.10831E-05

1.20801E-05
2.47292E-05
1.46653E-05
3.17793E-05
1.23108E-05
6.55862E-05
2.72309E-05
1.28816E-05
1.65537E-05
5.16201E-05
1.47255E-05
1.25602E-05
1.18960E-05
1.40898E-05
7.00581E-05
2.17048E-05
1.15231E-05
1.75566E-05
2.52878E-05
1.76489E-05
1.19339E-05
1.18926E-05
4.80704E-05
1.55164E-05
1.57691E-05
1.05129E-04
1.24085E-05
2.26943E-05
8.02639E-05
1.06277E-05
6.91966E-05
5.67010E-05
3.64907E-05
1.78484E-05
2.73295E-05
2.98113E-05
1.49941E-05
1.03249E-05
2.95422E-05
1.06652E-05
1.76857E-05
9.73517E-06
2.43095E-05
1.06982E-05
1.31603E-05
2.30550E-05
3.64659E-05
9.97773E-06
1.00723E-05
2.76530E-05
9.68136E-06
1.17199E-05
1.33795E-05
9.56157E-06

1.40184E-04
9.03424E-06
1.64624E-05
1.29102E-05
1.81090E-05
1.91876E-05
9.11649E-06
1.03386E-05
9.37913E-06
1.31512E-05
9.94986E-06
8.88038E-06
8.86875E-06
1.19167E-05
3.66415E-05
1.74095E-05
9.05034E-05
1.36481E-05
1.46062E-05
1.00488E-05
2.12917E-05
8.28761E-06
1.43119E-05
5.10038E-05
2.51133E-05
8.50060E-06
6.65457E-05
1.23095E-05
8.70891E-06
1.48578E-05
1.16776E-05
4.50498E-05
8.62251E-06
7.44738E-06
1.83578E-05
9.14191E-06
9.48297E-06
9.47174E-06
1.61566E-05
1.12854E-05
7.73421E-06
7.49885E-06
7.56276E-06
7.99490E-06
2.33771E-05
7.53940E-06
7.53711E-06
3.68666E-05
9.99016E-06
7.14938E-06
6.76371E-06
6.62691E-06
6.89920E-06
1.70478E-05

7.87544E-06
8.75996E-06
6.42804E-06
6.28409E-06
8.81331E-06
2.90987E-05
1.17299E-05
7.89131E-06
7.94829E-05
1.38756E-05
6.95721E-06
8.13341E-06
6.71829E-06
1.03268E-05
6.56114E-06
1.71971E-05
3.74901E-05
6.30278E-06
3.70101E-05
1.67105E-05
6.05397E-06
6.61040E-06
3.05246E-05
1.37712E-05
6.17438E-06
5.31948E-05
7.00616E-06
6.93785E-06
5.77836E-06
8.18193E-06
1.07338E-05
5.80813E-06
9.64868E-06
1.52192E-05
5.48776E-06
5.33098E-06
5.48512E-05
1.92159E-05
8.01216E-06
2.48533E-05
6.85509E-06
5.27415E-06
5.13967E-06
7.47471E-06
7.08354E-06
2.05676E-05
1.86564E-05
5.41409E-06
5.20959E-06
4.72767E-06
4.98647E-06
5.37717E-06
1.16770E-05
5.59605E-06

1.19409E-05
4.88920E-06
5.21362E-06
1.04246E-04
1.27704E-04
1.66309E-05
4.57732E-06
2.58698E-04
2.64104E-05
4.46604E-06
7.59699E-06
3.66764E-05
4.46435E-06
4.23225E-06
5.12187E-06
4.15148E-06
4.48004E-06
1.46510E-05
6.98559E-06
4.26377E-06
1.66602E-05
4.39433E-06
6.56539E-06
1.08838E-05
1.09881E-05
1.78571E-05
3.76415E-06
3.58156E-06
3.69412E-06
3.50143E-06
1.80892E-05
3.65378E-06
3.68933E-06
3.53773E-06
4.29283E-05
3.89592E-06
1.11387E-05
4.72231E-06
3.37114E-06
3.90629E-06
4.32652E-06
3.44834E-06
3.84262E-06
3.87053E-06
3.79440E-06
5.79189E-06
3.54407E-06
7.00705E-06
3.52546E-06
3.70322E-06
3.52104E-06
8.84563E-06
3.48268E-06
3.70184E-06

3.75195E-06
3.96209E-06
3.44360E-06
9.41764E-06
3.42977E-06
3.61937E-06
3.42734E-06
1.23084E-04
3.23802E-06
3.91716E-06
3.05834E-06
3.26428E-06
3.16909E-06
3.18180E-06
4.38558E-05
3.15615E-06
2.85989E-05
8.64838E-06
3.28031E-06
7.09111E-06
2.98878E-06
3.02355E-06
3.01772E-06
2.98594E-06
2.73525E-06
2.57233E-06
3.37481E-05
3.84389E-04
2.53225E-06
3.92009E-05
2.83911E-06
8.01020E-06
3.40424E-06
1.49764E-05
2.70179E-06
2.54524E-06
2.49861E-06
2.57116E-06
2.28510E-06
2.39915E-06
2.42631E-06
2.47444E-06
3.32360E-06
2.37020E-06
2.55198E-06
2.29428E-06
8.24815E-05
2.29668E-06
1.20725E-05
2.21445E-06
2.11530E-06
1.59968E-05
2.13773E-06
2.35323E-06

2.34416E-06
2.58349E-06
2.46480E-06
1.20388E-05
2.28868E-06
2.21705E-06
2.76666E-06
5.44939E-06
2.33067E-06
2.25172E-06
6.28572E-05
2.40187E-06
8.88518E-06
1.48811E-03
1.73724E-05
1.92156E-06
1.60656E-06
1.65108E-06
1.77428E-06
1.57346E-06
1.04881E-05
1.40313E-06
3.06501E-06
1.57290E-06
1.72424E-06
1.65736E-06
1.60230E-06
6.93581E-06
3.41327E-05
1.65539E-06
1.45600E-06
1.74390E-06
1.53038E-06
4.73325E-06
1.72270E-06
6.35919E-06
5.49957E-06
1.64597E-06
1.75644E-06
3.43912E-05
2.97777E-06
2.10272E-05
2.11417E-06
1.90228E-06
2.22970E-05
1.86190E-06
2.96544E-06
1.97085E-06
2.01948E-06
1.86286E-06
1.19071E-05
1.53036E-06
1.66331E-06
1.63496E-06

1.33693E-06
1.13205E-06
1.26052E-05
1.13979E-06
8.55675E-07
1.12319E-06
9.64558E-07
1.15892E-06
1.15678E-06
5.71814E-04
3.43481E-04
1.71900E-06
1.56412E-05
2.01886E-06
2.21904E-06
2.12901E-06
2.22530E-06
2.29857E-06
2.45842E-06
4.95177E-06
2.34640E-06
2.46032E-06
4.62685E-06
2.07768E-06
1.92779E-06
1.77086E-06
1.68857E-06
1.41080E-06
1.00027E-06
8.77770E-07
7.28201E-07
1.16424E-06
4.76485E-07
5.40756E-07
7.29321E-07
7.95641E-07
6.02098E-07
7.61883E-07
8.25576E-07
4.28685E-06
7.48257E-07
6.94049E-07
7.33851E-07
1.17484E-05
7.75567E-07
1.67433E-04
4.48415E-07
4.01313E-07
4.74111E-07
3.64592E-07
2.64836E-07
3.84729E-07
3.33804E-07
6.19488E-07

2.52588E-07
2.13572E-07
3.42374E-07
3.44155E-07
8.18513E-07
6.20610E-06
3.71981E-07
3.30675E-07
2.88509E-07
3.09858E-07
2.98160E-07
3.11821E-07
2.05127E-07
1.27975E-05
2.85996E-07
3.10845E-07
3.53803E-07
2.93500E-07
4.13400E-07
3.07402E-07
6.14618E-07
6.35632E-06
3.17609E-07
5.24764E-07
3.95765E-07
3.79960E-07
3.84000E-07
3.63650E-07
3.60072E-07
3.78333E-07
4.47884E-07
2.59668E-06
4.02860E-07
5.28072E-07
4.73629E-07
7.77517E-04
3.26556E-07
2.24687E-07
2.48439E-07
3.00047E-07
1.48505E-07
1.90556E-07
2.17341E-07
1.63342E-07
2.74146E-07
1.68832E-07
2.00196E-07
4.69251E-07
1.73588E-07
1.85672E-06
1.91409E-07
2.05848E-07
2.08294E-07
2.33555E-07

1.75082E-07
1.45887E-07
1.42870E-07
2.92197E-05
2.47916E-05
2.36770E-07
1.72662E-07
1.75031E-07
2.05721E-07
1.01668E-05
1.47880E-07
1.66215E-07
5.94565E-05
4.16023E-07
5.81728E-07
5.49419E-07
7.66059E-07
7.31296E-07
1.03896E-06
6.16223E-04
1.12198E-06
1.61144E-06
1.65855E-06
1.90723E-06
1.83057E-06
2.21895E-06
2.22540E-06
2.70806E-06
2.84087E-06
2.72981E-06
2.00179E-06
1.75644E-06
1.48792E-06
1.44159E-06
1.03569E-07
9.67483E-08
3.28510E-08
3.00000E-08
4.84090E-08
9.85305E-08
4.15117E-08
8.11212E-08
5.44535E-08
3.05221E-08
6.43666E-09
2.88052E-08
3.89996E-08
3.09803E-08
5.55854E-08
3.91180E-08
3.42506E-08
3.85257E-08
1.31177E-07
2.28760E-08

6.42335E-08
2.59369E-08
2.10209E-07
4.40737E-08
3.94707E-08
2.39075E-08
4.87211E-08
3.74063E-08
2.79981E-08
3.82181E-08
4.57271E-08
3.56661E-08
2.77517E-08
2.84674E-08
5.11066E-08
2.18992E-08
4.25037E-08
7.75376E-08
1.00908E-07
6.49251E-08
3.87643E-08
8.36343E-08
3.39288E-07
1.90181E-07
1.31831E-07
1.21471E-07
6.68722E-08
1.82321E-07
1.32649E-07
1.24357E-07
2.61819E-07
1.45376E-07
8.68511E-08
4.12717E-06
5.32249E-07
1.78661E-07
2.42407E-07
5.30295E-07
1.70137E-07
5.42083E-07
8.09373E-08
1.21668E-07
1.15000E-07
2.05979E-04
6.07596E-08
7.00000E-08
6.00000E-08
3.19289E-08
3.31470E-08
5.61696E-08
1.52202E-08
9.99992E-09
1.11112E-08
1.63314E-08

9.99996E-09
0.00000E+00
1.02202E-08
5.00000E-09
1.00417E-08
9.99992E-09
0.00000E+00
0.00000E+00
9.99996E-09
2.54402E-08
5.22015E-09
1.52201E-08
5.22015E-09
0.00000E+00
4.99996E-09
1.02504E-08
1.52201E-08
5.00000E-09
0.00000E+00
3.10000E-07
1.91338E-06
6.02201E-08
3.63825E-08
1.99999E-08
5.00000E-09
2.02201E-08
4.99996E-09
3.00000E-08
4.99996E-09
5.00000E-09
1.02202E-08
1.05000E-06
1.52201E-08
9.99992E-09
5.00000E-09
0.00000E+00
1.99999E-08
1.00000E-08
4.99996E-09
0.00000E+00
4.99996E-09
0.00000E+00
0.00000E+00
9.99992E-09
4.99996E-09
4.99996E-09
3.50000E-08
1.50000E-08
0.00000E+00
9.99996E-09
4.99996E-09
1.54403E-08
0.00000E+00
4.99996E-09

5.00000E-09
6.25835E-08
0.00000E+00
1.52201E-08
1.50000E-08
5.00000E-09
9.99996E-09
3.41500E-06
5.00000E-09
9.99996E-09
1.02202E-08
0.00000E+00
1.02201E-08
2.00000E-08
0.00000E+00
5.00000E-09
0.00000E+00
9.99996E-09
0.00000E+00
9.99996E-09
0.00000E+00
5.00000E-09
4.99996E-09
9.99996E-09
1.52201E-08
5.00000E-09
5.00000E-09
1.02201E-08
5.22015E-09
4.99996E-09
4.99996E-09
0.00000E+00
0.00000E+00
0.00000E+00
1.04055E-08
1.02202E-08
0.00000E+00
5.00000E-09
5.22015E-09
8.50000E-08
1.04403E-08
0.00000E+00
4.99996E-09
1.00000E-08
4.99996E-09
0.00000E+00
2.04403E-08
0.00000E+00
1.00000E-08
3.79508E-07
0.00000E+00
0.00000E+00
0.00000E+00
5.00000E-09

5.00000E-09
5.00000E-09
2.00000E-08
1.05000E-07
5.00000E-09
1.52201E-08
1.00000E-08
4.99996E-09
0.00000E+00
1.02201E-08
0.00000E+00
1.54403E-08
9.99996E-09
1.00000E-08
5.00000E-09
1.00000E-08
1.00000E-08
0.00000E+00
2.74515E-08
1.01652E-08
1.52201E-08
1.50000E-08
9.99996E-09
5.00000E-09
5.00000E-09
4.99996E-09
0.00000E+00
4.99996E-09
5.03143E-08
0.00000E+00
1.50000E-08
1.00000E-08
4.99996E-09
0.00000E+00
5.00000E-09
1.52201E-08
1.50000E-08
1.00000E-08
0.00000E+00
1.52201E-08
1.00000E-08
5.00000E-09
5.22015E-09
1.00000E-08
1.50000E-08
9.99996E-09
5.00000E-09
1.52201E-08
0.00000E+00
2.00000E-08
4.99996E-09
5.00000E-09
0.00000E+00
9.99996E-09

5.00000E-09
5.00000E-09
0.00000E+00
0.00000E+00
1.02201E-08
1.00000E-08
5.00000E-09
0.00000E+00
2.00000E-08
1.50000E-08
0.00000E+00
1.02201E-08
1.00000E-08
1.00000E-08
0.00000E+00
1.00000E-08
3.49999E-08
0.00000E+00
0.00000E+00
0.00000E+00
5.00000E-09
0.00000E+00
5.00000E-09
5.00000E-09
4.99996E-09
5.00000E-09
1.02201E-08
0.00000E+00
1.00000E-08
2.00000E-08
4.55000E-07
0.00000E+00
1.00000E-08
5.00000E-09
1.50000E-08
5.00000E-09
0.00000E+00
0.00000E+00
5.22015E-09
5.00000E-09
1.50000E-08
0.00000E+00
5.00000E-09
0.00000E+00
1.50000E-08
1.49999E-08
5.00000E-09
0.00000E+00
5.00000E-09
0.00000E+00
5.00000E-09
4.99996E-09
2.45000E-07
5.00000E-09

1.50000E-08
9.99996E-09
5.22015E-09
5.00000E-09
1.00000E-08
2.50000E-08
0.00000E+00
5.00000E-09
0.00000E+00
0.00000E+00
1.50000E-08
0.00000E+00
1.52201E-08
1.50000E-08
0.00000E+00
5.00000E-09
0.00000E+00
0.00000E+00
1.00000E-08
4.99996E-09
0.00000E+00
0.00000E+00
1.00000E-08
1.00000E-08
0.00000E+00
0.00000E+00
5.00000E-09
1.50000E-08
5.22015E-09
5.00000E-09
0.00000E+00
1.00000E-08
5.00000E-09
9.99996E-09
1.50000E-08
5.00000E-09
4.99996E-09
0.00000E+00
0.00000E+00
1.50000E-08
1.50000E-08
0.00000E+00
0.00000E+00
0.00000E+00
5.00000E-09
0.00000E+00
1.00000E-08
1.00000E-08
1.00000E-08
5.00000E-09
0.00000E+00
5.00000E-09
5.00000E-09
5.00000E-09

0.00000E+00
1.00000E-08
1.00000E-08
1.00000E-08
5.00000E-09
1.00000E-08
5.00000E-09
5.00000E-09
9.99996E-09
5.00000E-09
5.00000E-09
5.00000E-09
1.00000E-08
0.00000E+00
0.00000E+00
5.00000E-09
1.50000E-08
0.00000E+00
0.00000E+00
1.50000E-08
0.00000E+00
5.00000E-09
0.00000E+00
5.00000E-09
1.00000E-08
0.00000E+00
1.00000E-08
1.00000E-08
1.00000E-08
0.00000E+00
0.00000E+00
0.00000E+00
0.00000E+00
0.00000E+00
0.00000E+00
0.00000E+00
0.00000E+00
0.00000E+00
0.00000E+00
1.50000E-08
1.50000E-08
5.00000E-09
5.00000E-09
0.00000E+00
5.22015E-09
5.00000E-09
0.00000E+00
0.00000E+00
0.00000E+00
0.00000E+00
1.00000E-08
1.00000E-08
0.00000E+00
0.00000E+00

```
     0.00000E+00
     5.00000E-09
     0.00000E+00
     5.00000E-09
     5.00000E-09
     0.00000E+00
     0.00000E+00
     0.00000E+00
cut:p j 0.001                       $ Photon cut-off energy 1.0 keV
f8:p  1                             $ Pulse height tally defined for cell
1 (NaI detector)
e8    0.0 1.0e-5 0.01 1198i 12.0    $ Energy binning
ft8   geb  0.016410  0.076003  0.0  $ Tally treatment with GEB
nps   2.0e+8                        $ Number of histories
1
```
